# Supplementary material for: A double-blind, randomized controlled trial to examine the effect of Moringa oleifera leaf powder supplementation on the immune status and anthropometric parameters of adult HIV patients on antiretroviral therapy in a resource-limited setting
Source: PLoS One. 2021 Dec 31;16(12):e0261935. doi: 10.1371/journal.pone.0261935 (PMC8722362; doi:10.1371/journal.pone.0261935)
Supplement: S1 File — (PDF) [file pone.0261935.s002.pdf]

ASPIRATA
AUDITING • TESTING • CERTIFICATION
A MICROmega Group Company

sanas Testing Laboratory
T0760

Aspirata - Food & Beverage Laboratory
15 Lower Hope Road
Rosebank
7700
Cape Town

Tele: (021) 658 2770
Email: Michael.Waldner@aspirata.co.za

Certificate Of Analysis

NO: IUCA006/E-2017-01496
Customer: Aspirata KZN
Address: 359 King George V Avenue Glenwood Kwazulu Natal
Durban 4001
Contact: Jasomay Pillay
Order No: INTERNAL TRANSFER

Sample Description: Moringa Powder
No of Samples: 1
Sample Condition: Sealed Container
Date Received: 13-Apr-2017
Date Completed: 10-May-2017
Sample Identification: NI-17-00122

SAMPLE ID	ANALYTE	UNIT	METHOD	DETECTION LIMIT	RESULT
E-2017-01496	Arsenic*	mg/1000g	Subc	0.01	<0.01
E-2017-01496	Calcium*	mg/1000g	Subc		17918.2
E-2017-01496	Cadmium	mg/1000g	M10	0.01	<0.01
E-2017-01496	Copper	mg/1000g	M10	0.01	4.1
E-2017-01496	Iron	mg/1000g	M10	0.01	377.8
E-2017-01496	Potassium	mg/1000g	M10		48792.6
E-2017-01496	Lead	mg/1000g	M10	0.04	<0.04
E-2017-01496	Mercury (DMA)	mg/1000g	M34	0.03	<0.03
E-2017-01496	Zinc	mg/1000g	M10	0.14	28.8

This certificate shall not be reproduced, except in full, without the written approval of ASPIRATA. The certificate applies only to the sample(s) analysed or tested, and is subject to our standard terms and conditions, of which a copy is available on request. Please note that the necessary precautions with respect to the confidentiality of the information scanned and the distribution thereof is controlled and restricted access is maintained. This as part of the requirements of our quality system based on ISO 17025 standard.

The reported uncertainty is an expanded uncertainty calculated using a coverage factor of 2 which gives a level of confidence of approximately 95%.

Remarks relevant to the specific analysis: * Method is not SANAS accredited and is not included in the SANAS Schedule of accreditation for this laboratory

Michael Waldner - Technical Signatory

Lebo Maphage - Technical Signatory

DATE
2017/05/10

This COA has been electronically signed with an Advanced Electronic Signature (AES) as per the Electronic Communications and Transactions Act (No 25 of 2002). While the document remains in electronic format with a valid AES, it is considered to be the original document. If this document is printed, the printed version will be considered a copy.
