# Supplementary material for: A double-blind, randomized controlled trial to examine the effect of Moringa oleifera leaf powder supplementation on the immune status and anthropometric parameters of adult HIV patients on antiretroviral therapy in a resource-limited setting
Source: PLoS One. 2021 Dec 31;16(12):e0261935. doi: 10.1371/journal.pone.0261935 (PMC8722362; doi:10.1371/journal.pone.0261935)
Supplement: S2 File — (PDF) [file pone.0261935.s003.pdf]

ASPIRATA
AUDITING • TESTING • CERTIFICATION
A MICROmega Group Company

sanas Testing Laboratory

Aspirata - Durban
359 King George V Avenue
Glenwood, 4001
Durban, South Africa
VAT NO:49302 64538

Tel: (+27) 31 242 2300
Fax: (+27) 31 261 2509
Email: Jasomay.Pillay@aspirata.co.za

Nutritional

Certificate Of Analysis

Report NO:	NI-2017-00057	Sample Description:	Powder		
Customer:	Health Outcomes Research Unit(UKZN)				
Address:	Department of Public Health Medicine University of KwaZulu-Natal 2nd Glenwood Durban 4041	No of Samples	1	Sample Condition:	Room Temperature
		Date Received:	07-Apr-2017	Date Completed:	12-May-2017
		Sample Identification:	Moringa Powder		
Contact:	Indres Moodley				
Order No:	S460546				

		**Lab No**	NI-17-00122
		**Sample ID**	Moringa powder
**Analysis**	**Unit**	**Method**	
Carbohydrates	g/100g	Calculated	22
Energy	KJ /100g	Calculated	981
Glycaemic Carbohydrates	g/100g	MM-FB4	21
Moisture	g/100g	MM-FB1	5.3
Ash	g/100g	MM-FB1	9.3
Total Fat	g/100g	MM-FB3	3.9
Total Saturated Fat	g/100g	MM-FB3	1.4
Total monounsaturated Fat	g/100g	MM-FB3	2.1
Total Polyunsaturated Fat	g/100g	MM-FB3	0.4
Total Transfat in Product	g/100g	MM-FB3	<0.001
Total Transfat of Total Fat	% of Total Fat	MM-FB3	<0.01
Sodium(Na)	mg/100g	MM-FB5	24
Protein (Nx6.25)	g/100g	MM-FB6	28.2
Total Sugar Measured	g/100g	MM-FB2	7.6
Sucrose	g/100g	MM-FB2	7.6
Fructose	g/100g	MM-FB2	<0.007
Glucose	g/100g	MM-FB2	<0.02
Lactose	g/100g	MM-FB2	<0.03
Maltose	g/100g	MM-FB2	<0.03
Total Dietary Fibre	g/100g	MM-FB4	31.7

This report relates only to the samples actually supplied to and tested at Aspirata. The laboratory does not accept responsibility from any matters arising from the further use of these results. This certificate shall not be reproduced, except in full, without the written permission of the laboratory manager. No reference may be made to the Aspirata or any of its operating units or employees in advertisements or for sale or publicity purposes without the Aspirata's approval. All work is undertaken according to the Aspirata General Contract Conditions for Routine Testing. Samples are discarded after 30 days from issue date of certificate. Analytical records are discarded after 3 years.

Remarks: * Method is not SANAS accredited and is not included in the SANAS Schedule of accreditation for this laboratory.

J Pillay - Laboratory Supervisor

A copy of the original of this certificate is available from the Aspirata on request. This certificate is issued without any alteration or erasure.

Date Printed 12-May-2017

ASPIRATA
AUDITING • TESTING • CERTIFICATION
A MICROmega Group Company

Tel: (+27) 31 242 2300
Fax: (+27) 31 261 2509
Email: Jasomay.Pillay@aspirata.co.za

Aspirata - Durban
359 King George V Avenue
Glenwood, 4001
Durban, South Africa
VAT NO:49302 64538

sanas Testing Laboratory

Nutritional

Certificate Of Analysis

Report NO:	NI-2017-00057	Sample Description:	Powder		
Customer:	Health Outcomes Research Unit(UKZN)				
Address:	Department of Public Health Medicine University of KwaZulu-Natal 2nd Glenwood Durban 4041	No of Samples	1	Sample Condition:	Room Temperature
		Date Received:	07-Apr-2017	Date Completed:	12-May-2017
		Sample Identification:	Moringa Powder		
Contact:	Indres Moodley				
Order No:	S460546				

Comments *

The Energy value indicated is based on the glycaemic carbohydrates by assay.

The Energy value indicated on this report excludes the potential energy contribution from Total Dietary Fibre.

Lab method MM-FB4 for Total Dietary Fibre refers to Method: AOAC 985.29

The Total Dietary Fibre method used is ideally suited for test samples of plant origin. Although this method is recommended by the AOAC on all foodstuffs it may lead to inflated results on samples other than that of plant cell wall material.

Total Fat is the sum of Triglycerides.

Sodium Analysis via ICP.

This report relates only to the samples actually supplied to and tested at Aspirata. The laboratory does not accept responsibility from any matters arising from the further use of these results. This certificate shall not be reproduced, except in full, without the written permission of the laboratory manager. No reference may be made to the Aspirata or any of its operating units or employees in advertisements or for sale or publicity purposes without the Aspirata's approval. All work is undertaken according to the Aspirata General Contract Conditions for Routine Testing. Samples are discarded after 30 days from issue date of certificate. Analytical records are discarded after 3 years.

Remarks: * Method is not SANAS accredited and is not included in the SANAS Schedule of accreditation for this laboratory.

J Pillay - Laboratory Supervisor

A copy of the original of this certificate is available from the Aspirata on request. This certificate is issued without any alteration or erasure.

Date Printed 12-May-2017
