# Supplementary material for: A double-blind, randomized controlled trial to examine the effect of Moringa oleifera leaf powder supplementation on the immune status and anthropometric parameters of adult HIV patients on antiretroviral therapy in a resource-limited setting
Source: PLoS One. 2021 Dec 31;16(12):e0261935. doi: 10.1371/journal.pone.0261935 (PMC8722362; doi:10.1371/journal.pone.0261935)
Supplement: S3 File — (DOCX) [file pone.0261935.s004.docx]

| **Data Set Name** | WORK.ADMOLCRT | **Observations** | 1239 |
| --- | --- | --- | --- |
| **Member Type** | DATA | **Variables** | 21 |
| **Engine** | V9 | **Indexes** | 0 |
| **Created** | 15/08/2021 21:57:04 | **Observation Length** | 168 |
| **Last Modified** | 15/08/2021 21:57:04 | **Deleted Observations** | 0 |
| **Protection** |  | **Compressed** | NO |
| **Data Set Type** |  | **Sorted** | NO |
| **Label** |  |  |  |
| **Data Representation** | WINDOWS_64 |  |  |
| **Encoding** | wlatin1 Western (Windows) |  |  |

| **Engine/Host Dependent Information** | |
| --- | --- |
| **Data Set Page Size** | 65536 |
| **Number of Data Set Pages** | 4 |
| **First Data Page** | 1 |
| **Max Obs per Page** | 389 |
| **Obs in First Data Page** | 371 |
| **Number of Data Set Repairs** | 0 |
| **ExtendObsCounter** | YES |
| **Filename** | C:\Users\marot\AppData\Local\Temp\SAS Temporary Files\_TD4540_DESKTOP-LKEFK7L_\admolcrt.sas7bdat |
| **Release Created** | 9.0401M6 |
| **Host Created** | X64_10PRO |
| **Owner Name** | DESKTOP-LKEFK7L\marot |
| **File Size** | 320KB |
| **File Size (bytes)** | 327680 |

| **Alphabetic List of Variables and Attributes** | | | | | |
| --- | --- | --- | --- | --- | --- |
| **#** | **Variable** | **Type** | **Len** | **Format** | **Label** |
| **4** | AGE | Num | 8 | BEST. | AGE |
| **5** | AGEGR1 | Num | 8 | BEST. | AGEGR1 |
| **2** | ARM | Num | 8 | BEST. | ARM |
| **20** | BMI | Num | 8 | BEST. |  |
| **16** | CD4 | Num | 8 | COMMA15. |  |
| **9** | EDC | Num | 8 | BEST. | EDC |
| **11** | FAMMEM | Num | 8 | BEST. | FAMMEM |
| **12** | INC | Num | 8 | COMMA15. | INC |
| **13** | INGR1 | Num | 8 | BEST. | INGR1 |
| **8** | MARSTS | Num | 8 | BEST. | MARSTS |
| **21** | MUAC | Num | 8 | BEST. |  |
| **10** | OCC | Num | 8 | BEST. | OCC |
| **7** | RACE | Num | 8 | BEST. | RACE |
| **6** | RELG | Num | 8 | BEST. | RELG |
| **3** | SEX | Num | 8 | BEST. | SEX |
| **1** | SUBJID | Num | 8 | BEST. | SUBJID |
| **18** | VISIT | Num | 8 |  |  |
| **17** | VISITGR | Char | 8 |  |  |
| **14** | VL_0 | Num | 8 | BEST. | VL_0 |
| **15** | VL_6 | Num | 8 | BEST. | VL_6 |
| **19** | WGT | Num | 8 | BEST. |  |

| **Data Set Name** | DATAPATH.ADMOLCRT | **Observations** | 1239 |
| --- | --- | --- | --- |
| **Member Type** | DATA | **Variables** | 23 |
| **Engine** | V9 | **Indexes** | 0 |
| **Created** | 15/08/2021 21:57:05 | **Observation Length** | 184 |
| **Last Modified** | 15/08/2021 21:57:05 | **Deleted Observations** | 0 |
| **Protection** |  | **Compressed** | NO |
| **Data Set Type** |  | **Sorted** | NO |
| **Label** |  |  |  |
| **Data Representation** | WINDOWS_64 |  |  |
| **Encoding** | wlatin1 Western (Windows) |  |  |

| **Engine/Host Dependent Information** | |
| --- | --- |
| **Data Set Page Size** | 65536 |
| **Number of Data Set Pages** | 4 |
| **First Data Page** | 1 |
| **Max Obs per Page** | 355 |
| **Obs in First Data Page** | 336 |
| **Number of Data Set Repairs** | 0 |
| **ExtendObsCounter** | YES |
| **Filename** | C:\Users\marot\OneDrive - CAPRISA\Marothi [Statistician]\001Studies\Researchers OR Scientists\Sinaye\MOL RCT\Dataset\admolcrt.sas7bdat |
| **Release Created** | 9.0401M6 |
| **Host Created** | X64_10PRO |
| **Owner Name** | DESKTOP-LKEFK7L\marot |
| **File Size** | 320KB |
| **File Size (bytes)** | 327680 |

| **Alphabetic List of Variables and Attributes** | | | | | |
| --- | --- | --- | --- | --- | --- |
| **#** | **Variable** | **Type** | **Len** | **Format** | **Label** |
| **4** | AGE | Num | 8 | BEST. | Age |
| **5** | AGEGR1 | Num | 8 | AGEGR. | Age Group |
| **2** | ARM | Num | 8 | ARM. | Planned Arm |
| **21** | BMI | Num | 8 | BEST. | Body Mass Index |
| **19** | CD4 | Num | 8 | BEST8. | CD4 Count |
| **9** | EDC | Num | 8 | EDUC. | Education |
| **11** | FAMMEM | Num | 8 | FAMSZ. | Number of Family Members |
| **12** | INC | Num | 8 | COMMA15. | Annual Income |
| **13** | INGR1 | Num | 8 | INCSZ. | Annual Income Group |
| **8** | MARSTS | Num | 8 | MRTSTS. | Marital Status |
| **22** | MUAC | Num | 8 | BEST. | Mid-upper-arm circumference |
| **10** | OCC | Num | 8 | OCCP. | Occupation |
| **7** | RACE | Num | 8 | ENTH. | Race |
| **6** | RELG | Num | 8 | RLGN. | Religion |
| **3** | SEX | Num | 8 | GND. | Sex |
| **1** | SUBJID | Num | 8 | BEST. | Unique Subject Identifier |
| **17** | Time | Num | 8 |  | Time |
| **16** | VISIT | Num | 8 |  | Visit |
| **18** | VISITGR | Char | 8 |  | Visit Code |
| **23** | VL | Num | 8 |  |  |
| **14** | VL_0 | Num | 8 | VLD. | Viral Load at Baseline |
| **15** | VL_6 | Num | 8 | VLD. | Viral Load at Month 6 |
| **20** | WGT | Num | 8 | BEST. | Weight |

| **Moments** | | | |
| --- | --- | --- | --- |
| **N** | 88 | **Sum Weights** | 88 |
| **Mean** | 3.73443182 | **Sum Observations** | 328.63 |
| **Std Deviation** | 1.57304585 | **Variance** | 2.47447324 |
| **Skewness** | 0.50201074 | **Kurtosis** | 0.61308427 |
| **Uncorrected SS** | 1442.5255 | **Corrected SS** | 215.279172 |
| **Coeff Variation** | 42.1227625 | **Std Error Mean** | 0.16768725 |

| **Basic Statistical Measures** | | | |
| --- | --- | --- | --- |
| **Location** | | **Variability** | |
| **Mean** | 3.734432 | **Std Deviation** | 1.57305 |
| **Median** | 3.780000 | **Variance** | 2.47447 |
| **Mode** | 2.540000 | **Range** | 8.02000 |
|  |  | **Interquartile Range** | 2.20000 |

| ***Note: The mode displayed is the smallest of 2 modes with a count of 3.*** |
| --- |

| **Tests for Location: Mu0=0** | | | | |
| --- | --- | --- | --- | --- |
| **Test** | **Statistic** | | **p Value** | |
| **Student's t** | **t** | 22.27022 | **Pr > \|t\|** | <.0001 |
| **Sign** | **M** | 44 | **Pr >= \|M\|** | <.0001 |
| **Signed Rank** | **S** | 1958 | **Pr >= \|S\|** | <.0001 |

| **Tests for Normality** | | | | |
| --- | --- | --- | --- | --- |
| **Test** | **Statistic** | | **p Value** | |
| **Shapiro-Wilk** | **W** | 0.971791 | **Pr < W** | 0.0517 |
| **Kolmogorov-Smirnov** | **D** | 0.091072 | **Pr > D** | 0.0719 |
| **Cramer-von Mises** | **W-Sq** | 0.06309 | **Pr > W-Sq** | >0.2500 |
| **Anderson-Darling** | **A-Sq** | 0.485032 | **Pr > A-Sq** | 0.2288 |

| **Quantiles (Definition 5)** | |
| --- | --- |
| **Level** | **Quantile** |
| **100% Max** | 9.04 |
| **99%** | 9.04 |
| **95%** | 6.58 |
| **90%** | 5.82 |
| **75% Q3** | 4.75 |
| **50% Median** | 3.78 |
| **25% Q1** | 2.55 |
| **10%** | 1.62 |
| **5%** | 1.15 |
| **1%** | 1.02 |
| **0% Min** | 1.02 |

| **Extreme Observations** | | | |
| --- | --- | --- | --- |
| **Lowest** | | **Highest** | |
| **Value** | **Obs** | **Value** | **Obs** |
| 1.02 | 518 | 6.58 | 427 |
| 1.02 | 77 | 6.76 | 133 |
| 1.09 | 63 | 6.87 | 469 |
| 1.12 | 119 | 7.46 | 217 |
| 1.15 | 336 | 9.04 | 546 |

| **Moments** | | | |
| --- | --- | --- | --- |
| **N** | 89 | **Sum Weights** | 89 |
| **Mean** | 4.25752809 | **Sum Observations** | 378.92 |
| **Std Deviation** | 1.53758982 | **Variance** | 2.36418246 |
| **Skewness** | 0.70691446 | **Kurtosis** | 0.93771659 |
| **Uncorrected SS** | 1821.3106 | **Corrected SS** | 208.048056 |
| **Coeff Variation** | 36.1146136 | **Std Error Mean** | 0.1629842 |

| **Basic Statistical Measures** | | | |
| --- | --- | --- | --- |
| **Location** | | **Variability** | |
| **Mean** | 4.257528 | **Std Deviation** | 1.53759 |
| **Median** | 3.900000 | **Variance** | 2.36418 |
| **Mode** | 2.270000 | **Range** | 8.35000 |
|  |  | **Interquartile Range** | 2.20000 |

| ***Note: The mode displayed is the smallest of 2 modes with a count of 3.*** |
| --- |

| **Tests for Location: Mu0=0** | | | | |
| --- | --- | --- | --- | --- |
| **Test** | **Statistic** | | **p Value** | |
| **Student's t** | **t** | 26.12234 | **Pr > \|t\|** | <.0001 |
| **Sign** | **M** | 44.5 | **Pr >= \|M\|** | <.0001 |
| **Signed Rank** | **S** | 2002.5 | **Pr >= \|S\|** | <.0001 |

| **Tests for Normality** | | | | |
| --- | --- | --- | --- | --- |
| **Test** | **Statistic** | | **p Value** | |
| **Shapiro-Wilk** | **W** | 0.963315 | **Pr < W** | 0.0129 |
| **Kolmogorov-Smirnov** | **D** | 0.103731 | **Pr > D** | 0.0190 |
| **Cramer-von Mises** | **W-Sq** | 0.097091 | **Pr > W-Sq** | 0.1245 |
| **Anderson-Darling** | **A-Sq** | 0.659362 | **Pr > A-Sq** | 0.0859 |

| **Quantiles (Definition 5)** | |
| --- | --- |
| **Level** | **Quantile** |
| **100% Max** | 9.82 |
| **99%** | 9.82 |
| **95%** | 6.93 |
| **90%** | 5.98 |
| **75% Q3** | 5.33 |
| **50% Median** | 3.90 |
| **25% Q1** | 3.13 |
| **10%** | 2.39 |
| **5%** | 2.13 |
| **1%** | 1.47 |
| **0% Min** | 1.47 |

| **Extreme Observations** | | | |
| --- | --- | --- | --- |
| **Lowest** | | **Highest** | |
| **Value** | **Obs** | **Value** | **Obs** |
| 1.47 | 889 | 6.93 | 931 |
| 1.64 | 721 | 7.20 | 1211 |
| 1.98 | 854 | 7.45 | 952 |
| 2.01 | 714 | 8.17 | 1113 |
| 2.13 | 1120 | 9.82 | 945 |

| **Box-Cox Transformation Information for CD4 Count** | | | | |
| --- | --- | --- | --- | --- |
| **Lambda** |  | **R-Square** | **Log Like** |  |
| -2.0 |  | 0.05 | -236.763 |  |
| -1.9 |  | 0.05 | -224.431 |  |
| -1.8 |  | 0.05 | -212.493 |  |
| -1.7 |  | 0.05 | -200.965 |  |
| -1.6 |  | 0.05 | -189.865 |  |
| -1.5 |  | 0.05 | -179.208 |  |
| -1.4 |  | 0.04 | -169.011 |  |
| -1.3 |  | 0.04 | -159.288 |  |
| -1.2 |  | 0.04 | -150.052 |  |
| -1.1 |  | 0.04 | -141.317 |  |
| -1.0 |  | 0.04 | -133.092 |  |
| -0.9 |  | 0.04 | -125.388 |  |
| -0.8 |  | 0.04 | -118.212 |  |
| -0.7 |  | 0.04 | -111.570 |  |
| -0.6 |  | 0.04 | -105.465 |  |
| -0.5 |  | 0.04 | -99.899 |  |
| -0.4 |  | 0.04 | -94.874 |  |
| -0.3 |  | 0.04 | -90.387 |  |
| -0.2 |  | 0.04 | -86.435 |  |
| -0.1 |  | 0.04 | -83.015 |  |
| 0.0 |  | 0.04 | -80.119 |  |
| 0.1 |  | 0.04 | -77.741 |  |
| 0.2 |  | 0.03 | -75.873 |  |
| 0.3 |  | 0.03 | -74.507 | * |
| 0.4 |  | 0.03 | -73.633 | * |
| 0.5 | + | 0.03 | -73.242 | < |
| 0.6 |  | 0.03 | -73.324 | * |
| 0.7 |  | 0.03 | -73.868 | * |
| 0.8 |  | 0.03 | -74.865 | * |
| 0.9 |  | 0.03 | -76.305 |  |
| 1.0 |  | 0.03 | -78.177 |  |
| 1.1 |  | 0.03 | -80.473 |  |
| 1.2 |  | 0.03 | -83.182 |  |
| 1.3 |  | 0.03 | -86.295 |  |
| 1.4 |  | 0.02 | -89.802 |  |
| 1.5 |  | 0.02 | -93.696 |  |
| 1.6 |  | 0.02 | -97.967 |  |
| 1.7 |  | 0.02 | -102.606 |  |
| 1.8 |  | 0.02 | -107.606 |  |
| 1.9 |  | 0.02 | -112.957 |  |
| 2.0 |  | 0.02 | -118.651 |  |
| **< - Best Lambda * - 95% Confidence Interval + - Convenient Lambda** | | | | |

| **Model Statement Specification Details** | | | | |
| --- | --- | --- | --- | --- |
| **Type** | **DF** | **Variable** | **Description** | **Value** |
|  |  |  |  |  |
| **Dep** | 1 | BoxCox(CD4) | Lambda Used | 0.5 |
|  |  |  | Lambda | 0.5 |
|  |  |  | Log Likelihood | -73.2423 |
|  |  |  | Conv. Lambda | 0.5 |
|  |  |  | Conv. Lambda LL | -73.2423 |
|  |  |  | CI Limit | -75.1630 |
|  |  |  | Alpha | 0.05 |
|  |  |  | Label | CD4 Count |
|  |  |  |  |  |
| **Ind** | 1 | Class.ARMCOG | Label | Planned Arm COG |
|  |  |  |  |  |
| **Ind** | 0 | Class.ARMMOG | Options | Reference Level |
|  |  |  | Label | Planned Arm MOG |

| **Moments** | | | |
| --- | --- | --- | --- |
| **N** | 88 | **Sum Weights** | 88 |
| **Mean** | 1.88713035 | **Sum Observations** | 166.067471 |
| **Std Deviation** | 0.41852278 | **Variance** | 0.17516131 |
| **Skewness** | -0.1412925 | **Kurtosis** | -0.1018785 |
| **Uncorrected SS** | 328.63 | **Corrected SS** | 15.2390343 |
| **Coeff Variation** | 22.1777353 | **Std Error Mean** | 0.04461468 |

| **Basic Statistical Measures** | | | |
| --- | --- | --- | --- |
| **Location** | | **Variability** | |
| **Mean** | 1.887130 | **Std Deviation** | 0.41852 |
| **Median** | 1.944222 | **Variance** | 0.17516 |
| **Mode** | 1.593738 | **Range** | 1.99671 |
|  |  | **Interquartile Range** | 0.58258 |

| ***Note: The mode displayed is the smallest of 2 modes with a count of 3.*** |
| --- |

| **Tests for Location: Mu0=0** | | | | |
| --- | --- | --- | --- | --- |
| **Test** | **Statistic** | | **p Value** | |
| **Student's t** | **t** | 42.29842 | **Pr > \|t\|** | <.0001 |
| **Sign** | **M** | 44 | **Pr >= \|M\|** | <.0001 |
| **Signed Rank** | **S** | 1958 | **Pr >= \|S\|** | <.0001 |

| **Tests for Normality** | | | | |
| --- | --- | --- | --- | --- |
| **Test** | **Statistic** | | **p Value** | |
| **Shapiro-Wilk** | **W** | 0.981068 | **Pr < W** | 0.2266 |
| **Kolmogorov-Smirnov** | **D** | 0.089951 | **Pr > D** | 0.0789 |
| **Cramer-von Mises** | **W-Sq** | 0.107197 | **Pr > W-Sq** | 0.0914 |
| **Anderson-Darling** | **A-Sq** | 0.610361 | **Pr > A-Sq** | 0.1109 |

| **Quantiles (Definition 5)** | |
| --- | --- |
| **Level** | **Quantile** |
| **100% Max** | 3.00666 |
| **99%** | 3.00666 |
| **95%** | 2.56515 |
| **90%** | 2.41247 |
| **75% Q3** | 2.17945 |
| **50% Median** | 1.94422 |
| **25% Q1** | 1.59687 |
| **10%** | 1.27279 |
| **5%** | 1.07238 |
| **1%** | 1.00995 |
| **0% Min** | 1.00995 |

| **Extreme Observations** | | | |
| --- | --- | --- | --- |
| **Lowest** | | **Highest** | |
| **Value** | **Obs** | **Value** | **Obs** |
| 1.00995 | 518 | 2.56515 | 427 |
| 1.00995 | 77 | 2.60000 | 133 |
| 1.04403 | 63 | 2.62107 | 469 |
| 1.05830 | 119 | 2.73130 | 217 |
| 1.07238 | 336 | 3.00666 | 546 |

| **Moments** | | | |
| --- | --- | --- | --- |
| **N** | 89 | **Sum Weights** | 89 |
| **Mean** | 2.03035075 | **Sum Observations** | 180.701217 |
| **Std Deviation** | 0.36978417 | **Variance** | 0.13674033 |
| **Skewness** | 0.18664305 | **Kurtosis** | -0.0707657 |
| **Uncorrected SS** | 378.92 | **Corrected SS** | 12.0331494 |
| **Coeff Variation** | 18.2128222 | **Std Error Mean** | 0.03919704 |

| **Basic Statistical Measures** | | | |
| --- | --- | --- | --- |
| **Location** | | **Variability** | |
| **Mean** | 2.030351 | **Std Deviation** | 0.36978 |
| **Median** | 1.974842 | **Variance** | 0.13674 |
| **Mode** | 1.506652 | **Range** | 1.92125 |
|  |  | **Interquartile Range** | 0.53950 |

| ***Note: The mode displayed is the smallest of 2 modes with a count of 3.*** |
| --- |

| **Tests for Location: Mu0=0** | | | | |
| --- | --- | --- | --- | --- |
| **Test** | **Statistic** | | **p Value** | |
| **Student's t** | **t** | 51.79857 | **Pr > \|t\|** | <.0001 |
| **Sign** | **M** | 44.5 | **Pr >= \|M\|** | <.0001 |
| **Signed Rank** | **S** | 2002.5 | **Pr >= \|S\|** | <.0001 |

| **Tests for Normality** | | | | |
| --- | --- | --- | --- | --- |
| **Test** | **Statistic** | | **p Value** | |
| **Shapiro-Wilk** | **W** | 0.987855 | **Pr < W** | 0.5821 |
| **Kolmogorov-Smirnov** | **D** | 0.071116 | **Pr > D** | >0.1500 |
| **Cramer-von Mises** | **W-Sq** | 0.061273 | **Pr > W-Sq** | >0.2500 |
| **Anderson-Darling** | **A-Sq** | 0.372115 | **Pr > A-Sq** | >0.2500 |

| **Quantiles (Definition 5)** | |
| --- | --- |
| **Level** | **Quantile** |
| **100% Max** | 3.13369 |
| **99%** | 3.13369 |
| **95%** | 2.63249 |
| **90%** | 2.44540 |
| **75% Q3** | 2.30868 |
| **50% Median** | 1.97484 |
| **25% Q1** | 1.76918 |
| **10%** | 1.54596 |
| **5%** | 1.45945 |
| **1%** | 1.21244 |
| **0% Min** | 1.21244 |

| **Extreme Observations** | | | |
| --- | --- | --- | --- |
| **Lowest** | | **Highest** | |
| **Value** | **Obs** | **Value** | **Obs** |
| 1.21244 | 889 | 2.63249 | 931 |
| 1.28062 | 721 | 2.68328 | 1211 |
| 1.40712 | 854 | 2.72947 | 952 |
| 1.41774 | 714 | 2.85832 | 1113 |
| 1.45945 | 1120 | 3.13369 | 945 |

| **ARM** | **Method** | **N** | **Mean** | **Std Dev** | **Std Err** | **Minimum** | **Maximum** |
| --- | --- | --- | --- | --- | --- | --- | --- |
| **COG** |  | 88 | 3.7344 | 1.5730 | 0.1677 | 1.0200 | 9.0400 |
| **MOG** |  | 89 | 4.2575 | 1.5376 | 0.1630 | 1.4700 | 9.8200 |
| **Diff (1-2)** | **Pooled** |  | -0.5231 | 1.5553 | 0.2338 |  |  |
| **Diff (1-2)** | **Satterthwaite** |  | -0.5231 |  | 0.2338 |  |  |

| **ARM** | **Method** | **Mean** | **95% CL Mean** | | **Std Dev** | **95% CL Std Dev** | |
| --- | --- | --- | --- | --- | --- | --- | --- |
| **COG** |  | 3.7344 | 3.4011 | 4.0677 | 1.5730 | 1.3700 | 1.8472 |
| **MOG** |  | 4.2575 | 3.9336 | 4.5814 | 1.5376 | 1.3401 | 1.8038 |
| **Diff (1-2)** | **Pooled** | -0.5231 | -0.9846 | -0.0616 | 1.5553 | 1.4080 | 1.7373 |
| **Diff (1-2)** | **Satterthwaite** | -0.5231 | -0.9846 | -0.0616 |  |  |  |

| **Method** | **Variances** | **DF** | **t Value** | **Pr > \|t\|** |
| --- | --- | --- | --- | --- |
| **Pooled** | Equal | 175 | -2.24 | 0.0265 |
| **Satterthwaite** | Unequal | 174.8 | -2.24 | 0.0266 |

| **Equality of Variances** | | | | |
| --- | --- | --- | --- | --- |
| **Method** | **Num DF** | **Den DF** | **F Value** | **Pr > F** |
| **Folded F** | 87 | 88 | 1.05 | 0.8313 |

| **ARM** | **Method** | **N** | **Mean** | **Std Dev** | **Std Err** | **Minimum** | **Maximum** |
| --- | --- | --- | --- | --- | --- | --- | --- |
| **COG** |  | 88 | 1.8871 | 0.4185 | 0.0446 | 1.0100 | 3.0067 |
| **MOG** |  | 89 | 2.0304 | 0.3698 | 0.0392 | 1.2124 | 3.1337 |
| **Diff (1-2)** | **Pooled** |  | -0.1432 | 0.3948 | 0.0593 |  |  |
| **Diff (1-2)** | **Satterthwaite** |  | -0.1432 |  | 0.0594 |  |  |

| **ARM** | **Method** | **Mean** | **95% CL Mean** | | **Std Dev** | **95% CL Std Dev** | |
| --- | --- | --- | --- | --- | --- | --- | --- |
| **COG** |  | 1.8871 | 1.7985 | 1.9758 | 0.4185 | 0.3645 | 0.4915 |
| **MOG** |  | 2.0304 | 1.9525 | 2.1082 | 0.3698 | 0.3223 | 0.4338 |
| **Diff (1-2)** | **Pooled** | -0.1432 | -0.2603 | -0.0261 | 0.3948 | 0.3574 | 0.4410 |
| **Diff (1-2)** | **Satterthwaite** | -0.1432 | -0.2604 | -0.0260 |  |  |  |

| **Method** | **Variances** | **DF** | **t Value** | **Pr > \|t\|** |
| --- | --- | --- | --- | --- |
| **Pooled** | Equal | 175 | -2.41 | 0.0168 |
| **Satterthwaite** | Unequal | 171.89 | -2.41 | 0.0169 |

| **Equality of Variances** | | | | |
| --- | --- | --- | --- | --- |
| **Method** | **Num DF** | **Den DF** | **F Value** | **Pr > F** |
| **Folded F** | 87 | 88 | 1.28 | 0.2485 |

| **Moments** | | | |
| --- | --- | --- | --- |
| **N** | 88 | **Sum Weights** | 88 |
| **Mean** | 24.1929545 | **Sum Observations** | 2128.98 |
| **Std Deviation** | 4.09480471 | **Variance** | 16.7674257 |
| **Skewness** | 0.40219587 | **Kurtosis** | -0.2742134 |
| **Uncorrected SS** | 52965.0824 | **Corrected SS** | 1458.76603 |
| **Coeff Variation** | 16.9256083 | **Std Error Mean** | 0.43650765 |

| **Basic Statistical Measures** | | | |
| --- | --- | --- | --- |
| **Location** | | **Variability** | |
| **Mean** | 24.19295 | **Std Deviation** | 4.09480 |
| **Median** | 23.41500 | **Variance** | 16.76743 |
| **Mode** | 17.58000 | **Range** | 19.23000 |
|  |  | **Interquartile Range** | 6.52000 |

| ***Note: The mode displayed is the smallest of 7 modes with a count of 2.*** |
| --- |

| **Tests for Location: Mu0=0** | | | | |
| --- | --- | --- | --- | --- |
| **Test** | **Statistic** | | **p Value** | |
| **Student's t** | **t** | 55.4239 | **Pr > \|t\|** | <.0001 |
| **Sign** | **M** | 44 | **Pr >= \|M\|** | <.0001 |
| **Signed Rank** | **S** | 1958 | **Pr >= \|S\|** | <.0001 |

| **Tests for Normality** | | | | |
| --- | --- | --- | --- | --- |
| **Test** | **Statistic** | | **p Value** | |
| **Shapiro-Wilk** | **W** | 0.976637 | **Pr < W** | 0.1125 |
| **Kolmogorov-Smirnov** | **D** | 0.084409 | **Pr > D** | 0.1231 |
| **Cramer-von Mises** | **W-Sq** | 0.132752 | **Pr > W-Sq** | 0.0415 |
| **Anderson-Darling** | **A-Sq** | 0.707827 | **Pr > A-Sq** | 0.0657 |

| **Quantiles (Definition 5)** | |
| --- | --- |
| **Level** | **Quantile** |
| **100% Max** | 35.670 |
| **99%** | 35.670 |
| **95%** | 30.860 |
| **90%** | 29.940 |
| **75% Q3** | 27.550 |
| **50% Median** | 23.415 |
| **25% Q1** | 21.030 |
| **10%** | 19.530 |
| **5%** | 17.860 |
| **1%** | 16.440 |
| **0% Min** | 16.440 |

| **Extreme Observations** | | | |
| --- | --- | --- | --- |
| **Lowest** | | **Highest** | |
| **Value** | **Obs** | **Value** | **Obs** |
| 16.44 | 28 | 30.86 | 161 |
| 16.67 | 378 | 31.14 | 385 |
| 17.58 | 595 | 31.79 | 448 |
| 17.58 | 168 | 34.34 | 14 |
| 17.86 | 7 | 35.67 | 308 |

| **Moments** | | | |
| --- | --- | --- | --- |
| **N** | 89 | **Sum Weights** | 89 |
| **Mean** | 25.1620225 | **Sum Observations** | 2239.42 |
| **Std Deviation** | 4.92632404 | **Variance** | 24.2686686 |
| **Skewness** | 0.60414249 | **Kurtosis** | 0.23010705 |
| **Uncorrected SS** | 58483.9792 | **Corrected SS** | 2135.64284 |
| **Coeff Variation** | 19.5784105 | **Std Error Mean** | 0.5221893 |

| **Basic Statistical Measures** | | | |
| --- | --- | --- | --- |
| **Location** | | **Variability** | |
| **Mean** | 25.16202 | **Std Deviation** | 4.92632 |
| **Median** | 24.45000 | **Variance** | 24.26867 |
| **Mode** | 20.20000 | **Range** | 24.22000 |
|  |  | **Interquartile Range** | 6.93000 |

| ***Note: The mode displayed is the smallest of 5 modes with a count of 2.*** |
| --- |

| **Tests for Location: Mu0=0** | | | | |
| --- | --- | --- | --- | --- |
| **Test** | **Statistic** | | **p Value** | |
| **Student's t** | **t** | 48.18563 | **Pr > \|t\|** | <.0001 |
| **Sign** | **M** | 44.5 | **Pr >= \|M\|** | <.0001 |
| **Signed Rank** | **S** | 2002.5 | **Pr >= \|S\|** | <.0001 |

| **Tests for Normality** | | | | |
| --- | --- | --- | --- | --- |
| **Test** | **Statistic** | | **p Value** | |
| **Shapiro-Wilk** | **W** | 0.971287 | **Pr < W** | 0.0456 |
| **Kolmogorov-Smirnov** | **D** | 0.099696 | **Pr > D** | 0.0277 |
| **Cramer-von Mises** | **W-Sq** | 0.104631 | **Pr > W-Sq** | 0.0972 |
| **Anderson-Darling** | **A-Sq** | 0.658485 | **Pr > A-Sq** | 0.0863 |

| **Quantiles (Definition 5)** | |
| --- | --- |
| **Level** | **Quantile** |
| **100% Max** | 39.84 |
| **99%** | 39.84 |
| **95%** | 34.05 |
| **90%** | 32.04 |
| **75% Q3** | 28.65 |
| **50% Median** | 24.45 |
| **25% Q1** | 21.72 |
| **10%** | 19.56 |
| **5%** | 18.18 |
| **1%** | 15.62 |
| **0% Min** | 15.62 |

| **Extreme Observations** | | | |
| --- | --- | --- | --- |
| **Lowest** | | **Highest** | |
| **Value** | **Obs** | **Value** | **Obs** |
| 15.62 | 1078 | 34.05 | 1029 |
| 16.85 | 1141 | 34.60 | 1008 |
| 17.57 | 861 | 34.79 | 728 |
| 17.71 | 1022 | 39.16 | 819 |
| 18.18 | 630 | 39.84 | 889 |

| **Box-Cox Transformation Information for Body Mass Index** | | | | |
| --- | --- | --- | --- | --- |
| **Lambda** |  | **R-Square** | **Log Like** |  |
| -2.0 |  | 0.01 | -272.969 |  |
| -1.9 |  | 0.01 | -271.748 |  |
| -1.8 |  | 0.01 | -270.597 |  |
| -1.7 |  | 0.01 | -269.514 |  |
| -1.6 |  | 0.01 | -268.502 |  |
| -1.5 |  | 0.01 | -267.559 |  |
| -1.4 |  | 0.01 | -266.686 |  |
| -1.3 |  | 0.01 | -265.884 |  |
| -1.2 |  | 0.01 | -265.153 |  |
| -1.1 |  | 0.01 | -264.493 |  |
| -1.0 |  | 0.01 | -263.904 |  |
| -0.9 |  | 0.01 | -263.386 | * |
| -0.8 |  | 0.01 | -262.941 | * |
| -0.7 |  | 0.01 | -262.567 | * |
| -0.6 |  | 0.01 | -262.266 | * |
| -0.5 |  | 0.01 | -262.037 | * |
| -0.4 |  | 0.01 | -261.881 | * |
| -0.3 |  | 0.01 | -261.798 | * |
| -0.2 |  | 0.01 | -261.789 | < |
| -0.1 |  | 0.01 | -261.853 | * |
| 0.0 | + | 0.01 | -261.991 | * |
| 0.1 |  | 0.01 | -262.203 | * |
| 0.2 |  | 0.01 | -262.489 | * |
| 0.3 |  | 0.01 | -262.849 | * |
| 0.4 |  | 0.01 | -263.285 | * |
| 0.5 |  | 0.01 | -263.795 |  |
| 0.6 |  | 0.01 | -264.380 |  |
| 0.7 |  | 0.01 | -265.041 |  |
| 0.8 |  | 0.01 | -265.777 |  |
| 0.9 |  | 0.01 | -266.590 |  |
| 1.0 |  | 0.01 | -267.478 |  |
| 1.1 |  | 0.01 | -268.442 |  |
| 1.2 |  | 0.01 | -269.483 |  |
| 1.3 |  | 0.01 | -270.601 |  |
| 1.4 |  | 0.01 | -271.795 |  |
| 1.5 |  | 0.01 | -273.067 |  |
| 1.6 |  | 0.01 | -274.416 |  |
| 1.7 |  | 0.01 | -275.842 |  |
| 1.8 |  | 0.01 | -277.345 |  |
| 1.9 |  | 0.01 | -278.926 |  |
| 2.0 |  | 0.01 | -280.585 |  |
| **< - Best Lambda * - 95% Confidence Interval + - Convenient Lambda** | | | | |

| **Model Statement Specification Details** | | | | |
| --- | --- | --- | --- | --- |
| **Type** | **DF** | **Variable** | **Description** | **Value** |
|  |  |  |  |  |
| **Dep** | 1 | BoxCox(BMI) | Lambda Used | -0.2 |
|  |  |  | Lambda | -0.2 |
|  |  |  | Log Likelihood | -261.8 |
|  |  |  | Conv. Lambda | 0 |
|  |  |  | Conv. Lambda LL | -262.0 |
|  |  |  | CI Limit | -263.7 |
|  |  |  | Alpha | 0.05 |
|  |  |  | Label | Body Mass Index |
|  |  |  |  |  |
| **Ind** | 1 | Class.ARMCOG | Label | Planned Arm COG |
|  |  |  |  |  |
| **Ind** | 0 | Class.ARMMOG | Options | Reference Level |
|  |  |  | Label | Planned Arm MOG |

| **Moments** | | | |
| --- | --- | --- | --- |
| **N** | 88 | **Sum Weights** | 88 |
| **Mean** | 0.53054729 | **Sum Observations** | 46.6881618 |
| **Std Deviation** | 0.01786513 | **Variance** | 0.00031916 |
| **Skewness** | 0.04590014 | **Kurtosis** | -0.4913008 |
| **Uncorrected SS** | 24.798045 | **Corrected SS** | 0.02776717 |
| **Coeff Variation** | 3.36730216 | **Std Error Mean** | 0.00190443 |

| **Basic Statistical Measures** | | | |
| --- | --- | --- | --- |
| **Location** | | **Variability** | |
| **Mean** | 0.530547 | **Std Deviation** | 0.01787 |
| **Median** | 0.532233 | **Variance** | 0.0003192 |
| **Mode** | 0.510932 | **Range** | 0.08198 |
|  |  | **Interquartile Range** | 0.02859 |

| ***Note: The mode displayed is the smallest of 7 modes with a count of 2.*** |
| --- |

| **Tests for Location: Mu0=0** | | | | |
| --- | --- | --- | --- | --- |
| **Test** | **Statistic** | | **p Value** | |
| **Student's t** | **t** | 278.586 | **Pr > \|t\|** | <.0001 |
| **Sign** | **M** | 44 | **Pr >= \|M\|** | <.0001 |
| **Signed Rank** | **S** | 1958 | **Pr >= \|S\|** | <.0001 |

| **Tests for Normality** | | | | |
| --- | --- | --- | --- | --- |
| **Test** | **Statistic** | | **p Value** | |
| **Shapiro-Wilk** | **W** | 0.986799 | **Pr < W** | 0.5177 |
| **Kolmogorov-Smirnov** | **D** | 0.072917 | **Pr > D** | >0.1500 |
| **Cramer-von Mises** | **W-Sq** | 0.068735 | **Pr > W-Sq** | >0.2500 |
| **Anderson-Darling** | **A-Sq** | 0.414018 | **Pr > A-Sq** | >0.2500 |

| **Quantiles (Definition 5)** | |
| --- | --- |
| **Level** | **Quantile** |
| **100% Max** | 0.571241 |
| **99%** | 0.571241 |
| **95%** | 0.561854 |
| **90%** | 0.551899 |
| **75% Q3** | 0.543791 |
| **50% Median** | 0.532233 |
| **25% Q1** | 0.515200 |
| **10%** | 0.506699 |
| **5%** | 0.503641 |
| **1%** | 0.489260 |
| **0% Min** | 0.489260 |

| **Extreme Observations** | | | |
| --- | --- | --- | --- |
| **Lowest** | | **Highest** | |
| **Value** | **Obs** | **Value** | **Obs** |
| 0.489260 | 308 | 0.561854 | 7 |
| 0.492992 | 14 | 0.563633 | 168 |
| 0.500659 | 448 | 0.563633 | 595 |
| 0.502732 | 385 | 0.569656 | 378 |
| 0.503641 | 161 | 0.571241 | 28 |

| **Moments** | | | |
| --- | --- | --- | --- |
| **N** | 89 | **Sum Weights** | 89 |
| **Mean** | 0.52695292 | **Sum Observations** | 46.89881 |
| **Std Deviation** | 0.0202539 | **Variance** | 0.00041022 |
| **Skewness** | -0.0282242 | **Kurtosis** | -0.3513529 |
| **Uncorrected SS** | 24.7495643 | **Corrected SS** | 0.03609942 |
| **Coeff Variation** | 3.84358901 | **Std Error Mean** | 0.00214691 |

| **Basic Statistical Measures** | | | |
| --- | --- | --- | --- |
| **Location** | | **Variability** | |
| **Mean** | 0.526953 | **Std Deviation** | 0.02025 |
| **Median** | 0.527648 | **Variance** | 0.0004102 |
| **Mode** | 0.508616 | **Range** | 0.09856 |
|  |  | **Interquartile Range** | 0.02911 |

| ***Note: The mode displayed is the smallest of 5 modes with a count of 2.*** |
| --- |

| **Tests for Location: Mu0=0** | | | | |
| --- | --- | --- | --- | --- |
| **Test** | **Statistic** | | **p Value** | |
| **Student's t** | **t** | 245.4472 | **Pr > \|t\|** | <.0001 |
| **Sign** | **M** | 44.5 | **Pr >= \|M\|** | <.0001 |
| **Signed Rank** | **S** | 2002.5 | **Pr >= \|S\|** | <.0001 |

| **Tests for Normality** | | | | |
| --- | --- | --- | --- | --- |
| **Test** | **Statistic** | | **p Value** | |
| **Shapiro-Wilk** | **W** | 0.993113 | **Pr < W** | 0.9276 |
| **Kolmogorov-Smirnov** | **D** | 0.068176 | **Pr > D** | >0.1500 |
| **Cramer-von Mises** | **W-Sq** | 0.042024 | **Pr > W-Sq** | >0.2500 |
| **Anderson-Darling** | **A-Sq** | 0.240099 | **Pr > A-Sq** | >0.2500 |

| **Quantiles (Definition 5)** | |
| --- | --- |
| **Level** | **Quantile** |
| **100% Max** | 0.577117 |
| **99%** | 0.577117 |
| **95%** | 0.559862 |
| **90%** | 0.551730 |
| **75% Q3** | 0.540291 |
| **50% Median** | 0.527648 |
| **25% Q1** | 0.511181 |
| **10%** | 0.499875 |
| **5%** | 0.493829 |
| **1%** | 0.478560 |
| **0% Min** | 0.478560 |

| **Extreme Observations** | | | |
| --- | --- | --- | --- |
| **Lowest** | | **Highest** | |
| **Value** | **Obs** | **Value** | **Obs** |
| 0.478560 | 889 | 0.559862 | 630 |
| 0.480210 | 819 | 0.562803 | 1022 |
| 0.491710 | 728 | 0.563697 | 861 |
| 0.492249 | 1008 | 0.568434 | 1141 |
| 0.493829 | 1029 | 0.577117 | 1078 |

| **ARM** | **Method** | **N** | **Mean** | **Std Dev** | **Std Err** | **Minimum** | **Maximum** |
| --- | --- | --- | --- | --- | --- | --- | --- |
| **COG** |  | 88 | 24.1930 | 4.0948 | 0.4365 | 16.4400 | 35.6700 |
| **MOG** |  | 89 | 25.1620 | 4.9263 | 0.5222 | 15.6200 | 39.8400 |
| **Diff (1-2)** | **Pooled** |  | -0.9691 | 4.5321 | 0.6813 |  |  |
| **Diff (1-2)** | **Satterthwaite** |  | -0.9691 |  | 0.6806 |  |  |

| **ARM** | **Method** | **Mean** | **95% CL Mean** | | **Std Dev** | **95% CL Std Dev** | |
| --- | --- | --- | --- | --- | --- | --- | --- |
| **COG** |  | 24.1930 | 23.3253 | 25.0606 | 4.0948 | 3.5664 | 4.8086 |
| **MOG** |  | 25.1620 | 24.1243 | 26.1998 | 4.9263 | 4.2937 | 5.7793 |
| **Diff (1-2)** | **Pooled** | -0.9691 | -2.3137 | 0.3756 | 4.5321 | 4.1029 | 5.0623 |
| **Diff (1-2)** | **Satterthwaite** | -0.9691 | -2.3126 | 0.3745 |  |  |  |

| **Method** | **Variances** | **DF** | **t Value** | **Pr > \|t\|** |
| --- | --- | --- | --- | --- |
| **Pooled** | Equal | 175 | -1.42 | 0.1567 |
| **Satterthwaite** | Unequal | 169.99 | -1.42 | 0.1563 |

| **Equality of Variances** | | | | |
| --- | --- | --- | --- | --- |
| **Method** | **Num DF** | **Den DF** | **F Value** | **Pr > F** |
| **Folded F** | 88 | 87 | 1.45 | 0.0857 |

| **ARM** | **Method** | **N** | **Mean** | **Std Dev** | **Std Err** | **Minimum** | **Maximum** |
| --- | --- | --- | --- | --- | --- | --- | --- |
| **COG** |  | 88 | 0.5305 | 0.0179 | 0.00190 | 0.4893 | 0.5712 |
| **MOG** |  | 89 | 0.5270 | 0.0203 | 0.00215 | 0.4786 | 0.5771 |
| **Diff (1-2)** | **Pooled** |  | 0.00359 | 0.0191 | 0.00287 |  |  |
| **Diff (1-2)** | **Satterthwaite** |  | 0.00359 |  | 0.00287 |  |  |

| **ARM** | **Method** | **Mean** | **95% CL Mean** | | **Std Dev** | **95% CL Std Dev** | |
| --- | --- | --- | --- | --- | --- | --- | --- |
| **COG** |  | 0.5305 | 0.5268 | 0.5343 | 0.0179 | 0.0156 | 0.0210 |
| **MOG** |  | 0.5270 | 0.5227 | 0.5312 | 0.0203 | 0.0177 | 0.0238 |
| **Diff (1-2)** | **Pooled** | 0.00359 | -0.00207 | 0.00926 | 0.0191 | 0.0173 | 0.0213 |
| **Diff (1-2)** | **Satterthwaite** | 0.00359 | -0.00207 | 0.00926 |  |  |  |

| **Method** | **Variances** | **DF** | **t Value** | **Pr > \|t\|** |
| --- | --- | --- | --- | --- |
| **Pooled** | Equal | 175 | 1.25 | 0.2124 |
| **Satterthwaite** | Unequal | 172.77 | 1.25 | 0.2121 |

| **Equality of Variances** | | | | |
| --- | --- | --- | --- | --- |
| **Method** | **Num DF** | **Den DF** | **F Value** | **Pr > F** |
| **Folded F** | 88 | 87 | 1.29 | 0.2427 |

| **Moments** | | | |
| --- | --- | --- | --- |
| **N** | 88 | **Sum Weights** | 88 |
| **Mean** | 63.1590909 | **Sum Observations** | 5558 |
| **Std Deviation** | 13.48638 | **Variance** | 181.882445 |
| **Skewness** | 0.77722023 | **Kurtosis** | 0.99070778 |
| **Uncorrected SS** | 366862 | **Corrected SS** | 15823.7727 |
| **Coeff Variation** | 21.3530306 | **Std Error Mean** | 1.43765294 |

| **Basic Statistical Measures** | | | |
| --- | --- | --- | --- |
| **Location** | | **Variability** | |
| **Mean** | 63.15909 | **Std Deviation** | 13.48638 |
| **Median** | 61.50000 | **Variance** | 181.88245 |
| **Mode** | 53.00000 | **Range** | 71.00000 |
|  |  | **Interquartile Range** | 16.50000 |

| **Tests for Location: Mu0=0** | | | | |
| --- | --- | --- | --- | --- |
| **Test** | **Statistic** | | **p Value** | |
| **Student's t** | **t** | 43.93208 | **Pr > \|t\|** | <.0001 |
| **Sign** | **M** | 44 | **Pr >= \|M\|** | <.0001 |
| **Signed Rank** | **S** | 1958 | **Pr >= \|S\|** | <.0001 |

| **Tests for Normality** | | | | |
| --- | --- | --- | --- | --- |
| **Test** | **Statistic** | | **p Value** | |
| **Shapiro-Wilk** | **W** | 0.96305 | **Pr < W** | 0.0131 |
| **Kolmogorov-Smirnov** | **D** | 0.082472 | **Pr > D** | 0.1440 |
| **Cramer-von Mises** | **W-Sq** | 0.111383 | **Pr > W-Sq** | 0.0818 |
| **Anderson-Darling** | **A-Sq** | 0.752276 | **Pr > A-Sq** | 0.0486 |

| **Quantiles (Definition 5)** | |
| --- | --- |
| **Level** | **Quantile** |
| **100% Max** | 108.0 |
| **99%** | 108.0 |
| **95%** | 89.0 |
| **90%** | 81.0 |
| **75% Q3** | 69.5 |
| **50% Median** | 61.5 |
| **25% Q1** | 53.0 |
| **10%** | 46.0 |
| **5%** | 45.0 |
| **1%** | 37.0 |
| **0% Min** | 37.0 |

| **Extreme Observations** | | | |
| --- | --- | --- | --- |
| **Lowest** | | **Highest** | |
| **Value** | **Obs** | **Value** | **Obs** |
| 37 | 378 | 89 | 14 |
| 40 | 518 | 89 | 203 |
| 40 | 28 | 90 | 385 |
| 45 | 595 | 103 | 448 |
| 45 | 357 | 108 | 308 |

| **Moments** | | | |
| --- | --- | --- | --- |
| **N** | 89 | **Sum Weights** | 89 |
| **Mean** | 64.7078652 | **Sum Observations** | 5759 |
| **Std Deviation** | 15.0674259 | **Variance** | 227.027324 |
| **Skewness** | 0.58477612 | **Kurtosis** | 0.3427759 |
| **Uncorrected SS** | 392631 | **Corrected SS** | 19978.4045 |
| **Coeff Variation** | 23.2853083 | **Std Error Mean** | 1.59714395 |

| **Basic Statistical Measures** | | | |
| --- | --- | --- | --- |
| **Location** | | **Variability** | |
| **Mean** | 64.70787 | **Std Deviation** | 15.06743 |
| **Median** | 64.00000 | **Variance** | 227.02732 |
| **Mode** | 70.00000 | **Range** | 70.00000 |
|  |  | **Interquartile Range** | 18.00000 |

| ***Note: The mode displayed is the smallest of 2 modes with a count of 6.*** |
| --- |

| **Tests for Location: Mu0=0** | | | | |
| --- | --- | --- | --- | --- |
| **Test** | **Statistic** | | **p Value** | |
| **Student's t** | **t** | 40.51474 | **Pr > \|t\|** | <.0001 |
| **Sign** | **M** | 44.5 | **Pr >= \|M\|** | <.0001 |
| **Signed Rank** | **S** | 2002.5 | **Pr >= \|S\|** | <.0001 |

| **Tests for Normality** | | | | |
| --- | --- | --- | --- | --- |
| **Test** | **Statistic** | | **p Value** | |
| **Shapiro-Wilk** | **W** | 0.966328 | **Pr < W** | 0.0207 |
| **Kolmogorov-Smirnov** | **D** | 0.093045 | **Pr > D** | 0.0564 |
| **Cramer-von Mises** | **W-Sq** | 0.079793 | **Pr > W-Sq** | 0.2133 |
| **Anderson-Darling** | **A-Sq** | 0.634873 | **Pr > A-Sq** | 0.0961 |

| **Quantiles (Definition 5)** | |
| --- | --- |
| **Level** | **Quantile** |
| **100% Max** | 109 |
| **99%** | 109 |
| **95%** | 99 |
| **90%** | 82 |
| **75% Q3** | 72 |
| **50% Median** | 64 |
| **25% Q1** | 54 |
| **10%** | 45 |
| **5%** | 42 |
| **1%** | 39 |
| **0% Min** | 39 |

| **Extreme Observations** | | | |
| --- | --- | --- | --- |
| **Lowest** | | **Highest** | |
| **Value** | **Obs** | **Value** | **Obs** |
| 39 | 1078 | 99 | 819 |
| 39 | 861 | 99 | 1162 |
| 40 | 1085 | 100 | 1008 |
| 41 | 1141 | 102 | 889 |
| 42 | 1022 | 109 | 728 |

| **Box-Cox Transformation Information for Weight** | | | | |
| --- | --- | --- | --- | --- |
| **Lambda** |  | **R-Square** | **Log Like** |  |
| -2.0 |  | 0.00 | -484.863 |  |
| -1.9 |  | 0.00 | -482.802 |  |
| -1.8 |  | 0.00 | -480.843 |  |
| -1.7 |  | 0.00 | -478.985 |  |
| -1.6 |  | 0.00 | -477.230 |  |
| -1.5 |  | 0.00 | -475.580 |  |
| -1.4 |  | 0.00 | -474.035 |  |
| -1.3 |  | 0.00 | -472.596 |  |
| -1.2 |  | 0.00 | -471.265 |  |
| -1.1 |  | 0.00 | -470.042 |  |
| -1.0 |  | 0.00 | -468.929 |  |
| -0.9 |  | 0.00 | -467.926 |  |
| -0.8 |  | 0.00 | -467.035 |  |
| -0.7 |  | 0.00 | -466.256 |  |
| -0.6 |  | 0.00 | -465.591 | * |
| -0.5 |  | 0.00 | -465.039 | * |
| -0.4 |  | 0.00 | -464.603 | * |
| -0.3 |  | 0.00 | -464.282 | * |
| -0.2 |  | 0.00 | -464.078 | * |
| -0.1 |  | 0.00 | -463.990 | < |
| 0.0 | + | 0.00 | -464.021 | * |
| 0.1 |  | 0.00 | -464.170 | * |
| 0.2 |  | 0.00 | -464.438 | * |
| 0.3 |  | 0.00 | -464.825 | * |
| 0.4 |  | 0.00 | -465.332 | * |
| 0.5 |  | 0.00 | -465.959 |  |
| 0.6 |  | 0.00 | -466.707 |  |
| 0.7 |  | 0.00 | -467.575 |  |
| 0.8 |  | 0.00 | -468.565 |  |
| 0.9 |  | 0.00 | -469.675 |  |
| 1.0 |  | 0.00 | -470.907 |  |
| 1.1 |  | 0.00 | -472.259 |  |
| 1.2 |  | 0.00 | -473.732 |  |
| 1.3 |  | 0.00 | -475.326 |  |
| 1.4 |  | 0.00 | -477.041 |  |
| 1.5 |  | 0.00 | -478.875 |  |
| 1.6 |  | 0.00 | -480.829 |  |
| 1.7 |  | 0.00 | -482.903 |  |
| 1.8 |  | 0.00 | -485.095 |  |
| 1.9 |  | 0.00 | -487.405 |  |
| 2.0 |  | 0.00 | -489.833 |  |
| **< - Best Lambda * - 95% Confidence Interval + - Convenient Lambda** | | | | |

| **Model Statement Specification Details** | | | | |
| --- | --- | --- | --- | --- |
| **Type** | **DF** | **Variable** | **Description** | **Value** |
|  |  |  |  |  |
| **Dep** | 1 | BoxCox(WGT) | Lambda Used | -0.1 |
|  |  |  | Lambda | -0.1 |
|  |  |  | Log Likelihood | -464.0 |
|  |  |  | Conv. Lambda | 0 |
|  |  |  | Conv. Lambda LL | -464.0 |
|  |  |  | CI Limit | -465.9 |
|  |  |  | Alpha | 0.05 |
|  |  |  | Label | Weight |
|  |  |  |  |  |
| **Ind** | 1 | Class.ARMCOG | Label | Planned Arm COG |
|  |  |  |  |  |
| **Ind** | 0 | Class.ARMMOG | Options | Reference Level |
|  |  |  | Label | Planned Arm MOG |

| **Moments** | | | |
| --- | --- | --- | --- |
| **N** | 88 | **Sum Weights** | 88 |
| **Mean** | 0.66220375 | **Sum Observations** | 58.2739302 |
| **Std Deviation** | 0.01379714 | **Variance** | 0.00019036 |
| **Skewness** | -0.0594389 | **Kurtosis** | 0.06882367 |
| **Uncorrected SS** | 38.6057766 | **Corrected SS** | 0.01656142 |
| **Coeff Variation** | 2.08351906 | **Std Error Mean** | 0.00147078 |

| **Basic Statistical Measures** | | | |
| --- | --- | --- | --- |
| **Location** | | **Variability** | |
| **Mean** | 0.662204 | **Std Deviation** | 0.01380 |
| **Median** | 0.662390 | **Variance** | 0.0001904 |
| **Mode** | 0.672314 | **Range** | 0.07079 |
|  |  | **Interquartile Range** | 0.01798 |

| **Tests for Location: Mu0=0** | | | | |
| --- | --- | --- | --- | --- |
| **Test** | **Statistic** | | **p Value** | |
| **Student's t** | **t** | 450.2398 | **Pr > \|t\|** | <.0001 |
| **Sign** | **M** | 44 | **Pr >= \|M\|** | <.0001 |
| **Signed Rank** | **S** | 1958 | **Pr >= \|S\|** | <.0001 |

| **Tests for Normality** | | | | |
| --- | --- | --- | --- | --- |
| **Test** | **Statistic** | | **p Value** | |
| **Shapiro-Wilk** | **W** | 0.993448 | **Pr < W** | 0.9438 |
| **Kolmogorov-Smirnov** | **D** | 0.052253 | **Pr > D** | >0.1500 |
| **Cramer-von Mises** | **W-Sq** | 0.031262 | **Pr > W-Sq** | >0.2500 |
| **Anderson-Darling** | **A-Sq** | 0.210525 | **Pr > A-Sq** | >0.2500 |

| **Quantiles (Definition 5)** | |
| --- | --- |
| **Level** | **Quantile** |
| **100% Max** | 0.696915 |
| **99%** | 0.696915 |
| **95%** | 0.683406 |
| **90%** | 0.681906 |
| **75% Q3** | 0.672314 |
| **50% Median** | 0.662390 |
| **25% Q1** | 0.654339 |
| **10%** | 0.644394 |
| **5%** | 0.638353 |
| **1%** | 0.626120 |
| **0% Min** | 0.626120 |

| **Extreme Observations** | | | |
| --- | --- | --- | --- |
| **Lowest** | | **Highest** | |
| **Value** | **Obs** | **Value** | **Obs** |
| 0.626120 | 308 | 0.683406 | 357 |
| 0.629095 | 448 | 0.683406 | 595 |
| 0.637640 | 385 | 0.691503 | 28 |
| 0.638353 | 203 | 0.691503 | 518 |
| 0.638353 | 14 | 0.696915 | 378 |

| **Moments** | | | |
| --- | --- | --- | --- |
| **N** | 89 | **Sum Weights** | 89 |
| **Mean** | 0.66094708 | **Sum Observations** | 58.8242904 |
| **Std Deviation** | 0.01530873 | **Variance** | 0.00023436 |
| **Skewness** | 0.081737 | **Kurtosis** | -0.316058 |
| **Uncorrected SS** | 38.9003665 | **Corrected SS** | 0.02062342 |
| **Coeff Variation** | 2.3161802 | **Std Error Mean** | 0.00162272 |

| **Basic Statistical Measures** | | | |
| --- | --- | --- | --- |
| **Location** | | **Variability** | |
| **Mean** | 0.660947 | **Std Deviation** | 0.01531 |
| **Median** | 0.659754 | **Variance** | 0.0002344 |
| **Mode** | 0.646831 | **Range** | 0.06771 |
|  |  | **Interquartile Range** | 0.01903 |

| ***Note: The mode displayed is the smallest of 2 modes with a count of 6.*** |
| --- |

| **Tests for Location: Mu0=0** | | | | |
| --- | --- | --- | --- | --- |
| **Test** | **Statistic** | | **p Value** | |
| **Student's t** | **t** | 407.3077 | **Pr > \|t\|** | <.0001 |
| **Sign** | **M** | 44.5 | **Pr >= \|M\|** | <.0001 |
| **Signed Rank** | **S** | 2002.5 | **Pr >= \|S\|** | <.0001 |

| **Tests for Normality** | | | | |
| --- | --- | --- | --- | --- |
| **Test** | **Statistic** | | **p Value** | |
| **Shapiro-Wilk** | **W** | 0.984631 | **Pr < W** | 0.3777 |
| **Kolmogorov-Smirnov** | **D** | 0.081623 | **Pr > D** | 0.1482 |
| **Cramer-von Mises** | **W-Sq** | 0.069913 | **Pr > W-Sq** | >0.2500 |
| **Anderson-Darling** | **A-Sq** | 0.41026 | **Pr > A-Sq** | >0.2500 |

| **Quantiles (Definition 5)** | |
| --- | --- |
| **Level** | **Quantile** |
| **100% Max** | 0.693256 |
| **99%** | 0.693256 |
| **95%** | 0.688137 |
| **90%** | 0.683406 |
| **75% Q3** | 0.671059 |
| **50% Median** | 0.659754 |
| **25% Q1** | 0.652029 |
| **10%** | 0.643604 |
| **5%** | 0.631592 |
| **1%** | 0.625543 |
| **0% Min** | 0.625543 |

| **Extreme Observations** | | | |
| --- | --- | --- | --- |
| **Lowest** | | **Highest** | |
| **Value** | **Obs** | **Value** | **Obs** |
| 0.625543 | 728 | 0.688137 | 1022 |
| 0.629709 | 889 | 0.689797 | 1141 |
| 0.630957 | 1008 | 0.691503 | 1085 |
| 0.631592 | 1162 | 0.693256 | 861 |
| 0.631592 | 819 | 0.693256 | 1078 |

| **ARM** | **Method** | **N** | **Mean** | **Std Dev** | **Std Err** | **Minimum** | **Maximum** |
| --- | --- | --- | --- | --- | --- | --- | --- |
| **COG** |  | 88 | 63.1591 | 13.4864 | 1.4377 | 37.0000 | 108.0 |
| **MOG** |  | 89 | 64.7079 | 15.0674 | 1.5971 | 39.0000 | 109.0 |
| **Diff (1-2)** | **Pooled** |  | -1.5488 | 14.3033 | 2.1502 |  |  |
| **Diff (1-2)** | **Satterthwaite** |  | -1.5488 |  | 2.1489 |  |  |

| **ARM** | **Method** | **Mean** | **95% CL Mean** | | **Std Dev** | **95% CL Std Dev** | |
| --- | --- | --- | --- | --- | --- | --- | --- |
| **COG** |  | 63.1591 | 60.3016 | 66.0166 | 13.4864 | 11.7459 | 15.8371 |
| **MOG** |  | 64.7079 | 61.5339 | 67.8819 | 15.0674 | 13.1325 | 17.6763 |
| **Diff (1-2)** | **Pooled** | -1.5488 | -5.7925 | 2.6950 | 14.3033 | 12.9489 | 15.9766 |
| **Diff (1-2)** | **Satterthwaite** | -1.5488 | -5.7901 | 2.6926 |  |  |  |

| **Method** | **Variances** | **DF** | **t Value** | **Pr > \|t\|** |
| --- | --- | --- | --- | --- |
| **Pooled** | Equal | 175 | -0.72 | 0.4723 |
| **Satterthwaite** | Unequal | 173.3 | -0.72 | 0.4720 |

| **Equality of Variances** | | | | |
| --- | --- | --- | --- | --- |
| **Method** | **Num DF** | **Den DF** | **F Value** | **Pr > F** |
| **Folded F** | 88 | 87 | 1.25 | 0.3019 |

| **ARM** | **Method** | **N** | **Mean** | **Std Dev** | **Std Err** | **Minimum** | **Maximum** |
| --- | --- | --- | --- | --- | --- | --- | --- |
| **COG** |  | 88 | 0.6622 | 0.0138 | 0.00147 | 0.6261 | 0.6969 |
| **MOG** |  | 89 | 0.6609 | 0.0153 | 0.00162 | 0.6255 | 0.6933 |
| **Diff (1-2)** | **Pooled** |  | 0.00126 | 0.0146 | 0.00219 |  |  |
| **Diff (1-2)** | **Satterthwaite** |  | 0.00126 |  | 0.00219 |  |  |

| **ARM** | **Method** | **Mean** | **95% CL Mean** | | **Std Dev** | **95% CL Std Dev** | |
| --- | --- | --- | --- | --- | --- | --- | --- |
| **COG** |  | 0.6622 | 0.6593 | 0.6651 | 0.0138 | 0.0120 | 0.0162 |
| **MOG** |  | 0.6609 | 0.6577 | 0.6642 | 0.0153 | 0.0133 | 0.0180 |
| **Diff (1-2)** | **Pooled** | 0.00126 | -0.00307 | 0.00558 | 0.0146 | 0.0132 | 0.0163 |
| **Diff (1-2)** | **Satterthwaite** | 0.00126 | -0.00307 | 0.00558 |  |  |  |

| **Method** | **Variances** | **DF** | **t Value** | **Pr > \|t\|** |
| --- | --- | --- | --- | --- |
| **Pooled** | Equal | 175 | 0.57 | 0.5671 |
| **Satterthwaite** | Unequal | 173.52 | 0.57 | 0.5668 |

| **Equality of Variances** | | | | |
| --- | --- | --- | --- | --- |
| **Method** | **Num DF** | **Den DF** | **F Value** | **Pr > F** |
| **Folded F** | 88 | 87 | 1.23 | 0.3329 |

| **MONTHS** | **Month0** | **Month1** | **Month2** | **Month3** | **Month4** | **Month5** | **Month6** |
| --- | --- | --- | --- | --- | --- | --- | --- |
| Month0 | 1.34985 | 1.39321 | 1.36891 | 1.37144 | 1.34397 | 1.30295 | 1.31899 |
| Month1 | 1.39321 | 1.65715 | 1.62226 | 1.62908 | 1.60531 | 1.57129 | 1.58419 |
| Month2 | 1.36891 | 1.62226 | 1.88842 | 1.81332 | 1.85634 | 1.83203 | 1.85608 |
| Month3 | 1.37144 | 1.62908 | 1.81332 | 1.91591 | 1.91620 | 1.88336 | 1.89782 |
| Month4 | 1.34397 | 1.60531 | 1.85634 | 1.91620 | 2.11154 | 2.09131 | 2.14130 |
| Month5 | 1.30295 | 1.57129 | 1.83203 | 1.88336 | 2.09131 | 2.22123 | 2.28659 |
| Month6 | 1.31899 | 1.58419 | 1.85608 | 1.89782 | 2.14130 | 2.28659 | 2.47406 |

| **MONTHS** | **Month0** | **Month1** | **Month2** | **Month3** | **Month4** | **Month5** | **Month6** |
| --- | --- | --- | --- | --- | --- | --- | --- |
| Month0 | 1.00000 | 0.93152 | 0.85740 | 0.85280 | 0.79607 | 0.75247 | 0.72176 |
| Month1 | 0.93152 | 1.00000 | 0.91705 | 0.91427 | 0.85818 | 0.81899 | 0.78238 |
| Month2 | 0.85740 | 0.91705 | 1.00000 | 0.95332 | 0.92963 | 0.89451 | 0.85870 |
| Month3 | 0.85280 | 0.91427 | 0.95332 | 1.00000 | 0.95269 | 0.91295 | 0.87169 |
| Month4 | 0.79607 | 0.85818 | 0.92963 | 0.95269 | 1.00000 | 0.96566 | 0.93685 |
| Month5 | 0.75247 | 0.81899 | 0.89451 | 0.91295 | 0.96566 | 1.00000 | 0.97541 |
| Month6 | 0.72176 | 0.78238 | 0.85870 | 0.87169 | 0.93685 | 0.97541 | 1.00000 |

| **MONTHS** | **Month0** | **Month1** | **Month2** | **Month3** | **Month4** | **Month5** | **Month6** |
| --- | --- | --- | --- | --- | --- | --- | --- |
| Month0 | 18.9433 | 19.1387 | 19.0862 | 19.1255 | 19.1035 | 19.0948 | 19.0763 |
| Month1 | 19.1387 | 19.6746 | 19.5999 | 19.6617 | 19.6397 | 19.6668 | 19.6338 |
| Month2 | 19.0862 | 19.5999 | 19.8816 | 19.9212 | 19.9207 | 19.9182 | 19.8689 |
| Month3 | 19.1255 | 19.6617 | 19.9212 | 20.2766 | 20.2276 | 20.2388 | 20.1688 |
| Month4 | 19.1035 | 19.6397 | 19.9207 | 20.2276 | 20.4484 | 20.4106 | 20.3300 |
| Month5 | 19.0948 | 19.6668 | 19.9182 | 20.2388 | 20.4106 | 20.6536 | 20.5526 |
| Month6 | 19.0763 | 19.6338 | 19.8689 | 20.1688 | 20.3300 | 20.5526 | 20.6589 |

| **MONTHS** | **Month0** | **Month1** | **Month2** | **Month3** | **Month4** | **Month5** | **Month6** |
| --- | --- | --- | --- | --- | --- | --- | --- |
| Month0 | 1.00000 | 0.99136 | 0.98348 | 0.97586 | 0.97063 | 0.96536 | 0.96430 |
| Month1 | 0.99136 | 1.00000 | 0.99100 | 0.98440 | 0.97916 | 0.97562 | 0.97386 |
| Month2 | 0.98348 | 0.99100 | 1.00000 | 0.99219 | 0.98798 | 0.98294 | 0.98038 |
| Month3 | 0.97586 | 0.98440 | 0.99219 | 1.00000 | 0.99338 | 0.98899 | 0.98544 |
| Month4 | 0.97063 | 0.97916 | 0.98798 | 0.99338 | 1.00000 | 0.99318 | 0.98913 |
| Month5 | 0.96536 | 0.97562 | 0.98294 | 0.98899 | 0.99318 | 1.00000 | 0.99498 |
| Month6 | 0.96430 | 0.97386 | 0.98038 | 0.98544 | 0.98913 | 0.99498 | 1.00000 |

| **MONTHS** | **Month0** | **Month1** | **Month2** | **Month3** | **Month4** | **Month5** | **Month6** |
| --- | --- | --- | --- | --- | --- | --- | --- |
| Month0 | 187.687 | 189.882 | 190.249 | 191.144 | 190.520 | 190.366 | 191.249 |
| Month1 | 189.882 | 194.311 | 194.491 | 195.540 | 194.916 | 195.006 | 195.804 |
| Month2 | 190.249 | 194.491 | 196.674 | 197.549 | 197.022 | 196.912 | 197.676 |
| Month3 | 191.144 | 195.540 | 197.549 | 200.497 | 199.713 | 199.667 | 200.317 |
| Month4 | 190.520 | 194.916 | 197.022 | 199.713 | 200.627 | 200.287 | 200.873 |
| Month5 | 190.366 | 195.006 | 196.912 | 199.667 | 200.287 | 201.732 | 202.247 |
| Month6 | 191.249 | 195.804 | 197.676 | 200.317 | 200.873 | 202.247 | 204.025 |

| **MONTHS** | **Month0** | **Month1** | **Month2** | **Month3** | **Month4** | **Month5** | **Month6** |
| --- | --- | --- | --- | --- | --- | --- | --- |
| Month0 | 1.00000 | 0.99430 | 0.99022 | 0.98534 | 0.98181 | 0.97833 | 0.97733 |
| Month1 | 0.99430 | 1.00000 | 0.99490 | 0.99068 | 0.98720 | 0.98495 | 0.98341 |
| Month2 | 0.99022 | 0.99490 | 1.00000 | 0.99483 | 0.99185 | 0.98858 | 0.98682 |
| Month3 | 0.98534 | 0.99068 | 0.99483 | 1.00000 | 0.99577 | 0.99281 | 0.99043 |
| Month4 | 0.98181 | 0.98720 | 0.99185 | 0.99577 | 1.00000 | 0.99557 | 0.99286 |
| Month5 | 0.97833 | 0.98495 | 0.98858 | 0.99281 | 0.99557 | 1.00000 | 0.99691 |
| Month6 | 0.97733 | 0.98341 | 0.98682 | 0.99043 | 0.99286 | 0.99691 | 1.00000 |

| **Model Information** | |
| --- | --- |
| **Data Set** | DATAPATH.ADMOLCRT |
| **Dependent Variable** | CD4 |
| **Covariance Structure** | Unstructured |
| **Subject Effect** | SUBJID |
| **Estimation Method** | REML |
| **Residual Variance Method** | Profile |
| **Fixed Effects SE Method** | Model-Based |
| **Degrees of Freedom Method** | Containment |

| **Class Level Information** | | |
| --- | --- | --- |
| **Class** | **Levels** | **Values** |
| **SUBJID** | 177 | 90 91 92 93 94 95 96 97 98 99 100 101 102 103 104 105 106 107 108 109 110 111 112 113 114 115 116 117 118 119 120 121 122 123 124 125 126 127 128 129 130 131 132 133 134 135 136 137 138 139 140 141 142 143 144 145 146 147 148 149 150 151 152 153 154 155 156 157 158 159 160 161 162 163 164 165 166 167 168 169 170 171 172 173 174 175 176 177 1 2 3 4 5 6 7 8 9 10 11 12 13 14 15 16 17 18 19 20 21 22 23 24 25 26 27 28 29 30 31 32 33 34 35 36 37 38 39 40 41 42 43 44 45 46 47 48 49 50 51 52 53 54 55 56 57 58 59 60 61 62 63 64 65 66 67 68 69 70 71 72 73 74 75 76 77 78 79 80 81 82 83 84 85 86 87 88 89 |
| **ARM** | 2 | MOG COG |
| **VISITGR** | 7 | 1 2 3 4 5 6 0 |

| **Dimensions** | |
| --- | --- |
| **Covariance Parameters** | 4 |
| **Columns in X** | 6 |
| **Columns in Z per Subject** | 2 |
| **Subjects** | 177 |
| **Max Obs per Subject** | 7 |

| **Number of Observations** | |
| --- | --- |
| **Number of Observations Read** | 1239 |
| **Number of Observations Used** | 1239 |
| **Number of Observations Not Used** | 0 |

| **Iteration History** | | | |
| --- | --- | --- | --- |
| **Iteration** | **Evaluations** | **-2 Res Log Like** | **Criterion** |
| **0** | 1 | 4338.92460896 |  |
| **1** | 1 | 1943.78351480 | 0.00000000 |

| Convergence criteria met. |
| --- |

| **Covariance Parameter Estimates** | | | | | |
| --- | --- | --- | --- | --- | --- |
| **Cov Parm** | **Subject** | **Estimate** | **Standard Error** | **Z Value** | **Pr Z** |
| **UN(1,1)** | SUBJID | 1.4605 | 0.1615 | 9.05 | <.0001 |
| **UN(2,1)** | SUBJID | 0.002029 | 0.01642 | 0.12 | 0.9016 |
| **UN(2,2)** | SUBJID | 0.02732 | 0.003334 | 8.19 | <.0001 |
| **Residual** |  | 0.1070 | 0.005087 | 21.04 | <.0001 |

| **Fit Statistics** | |
| --- | --- |
| **-2 Res Log Likelihood** | 1943.8 |
| **AIC (Smaller is Better)** | 1951.8 |
| **AICC (Smaller is Better)** | 1951.8 |
| **BIC (Smaller is Better)** | 1964.5 |

| **Null Model Likelihood Ratio Test** | | |
| --- | --- | --- |
| **DF** | **Chi-Square** | **Pr > ChiSq** |
| 3 | 2395.14 | <.0001 |

| **Solution for Fixed Effects** | | | | | | |
| --- | --- | --- | --- | --- | --- | --- |
| **Effect** | **Planned Arm** | **Estimate** | **Standard Error** | **DF** | **t Value** | **Pr > \|t\|** |
| **Intercept** |  | 3.5635 | 0.1310 | 175 | 27.20 | <.0001 |
| **ARM** | MOG | -0.1055 | 0.1847 | 885 | -0.57 | 0.5680 |
| **ARM** | COG | 0 | . | . | . | . |
| **VISIT** |  | 0.03355 | 0.01881 | 175 | 1.78 | 0.0762 |
| **VISIT*ARM** | MOG | 0.1033 | 0.02653 | 885 | 3.89 | 0.0001 |
| **VISIT*ARM** | COG | 0 | . | . | . | . |

| **Type 3 Tests of Fixed Effects** | | | | |
| --- | --- | --- | --- | --- |
| **Effect** | **Num DF** | **Den DF** | **F Value** | **Pr > F** |
| **ARM** | 1 | 885 | 0.33 | 0.5680 |
| **VISIT** | 1 | 175 | 41.25 | <.0001 |
| **VISIT*ARM** | 1 | 885 | 15.16 | 0.0001 |

| **Estimates** | | | | | | | | |
| --- | --- | --- | --- | --- | --- | --- | --- | --- |
| **Label** | **Estimate** | **Standard Error** | **DF** | **t Value** | **Pr > \|t\|** | **Alpha** | **Lower** | **Upper** |
| **Treatment effect overtime** | 0.1033 | 0.02653 | 885 | 3.89 | 0.0001 | 0.05 | 0.05122 | 0.1554 |

| **Least Squares Means** | | | | | | |
| --- | --- | --- | --- | --- | --- | --- |
| **Effect** | **Planned Arm** | **Estimate** | **Standard Error** | **DF** | **t Value** | **Pr > \|t\|** |
| **ARM** | MOG | 3.8684 | 0.1396 | 885 | 27.72 | <.0001 |
| **ARM** | COG | 3.6641 | 0.1404 | 885 | 26.10 | <.0001 |

| **Differences of Least Squares Means** | | | | | | | |
| --- | --- | --- | --- | --- | --- | --- | --- |
| **Effect** | **Planned Arm** | **Planned Arm** | **Estimate** | **Standard Error** | **DF** | **t Value** | **Pr > \|t\|** |
| **ARM** | MOG | COG | 0.2043 | 0.1980 | 885 | 1.03 | 0.3022 |

| **Model Information** | |
| --- | --- |
| **Data Set** | DATAPATH.ADMOLCRT |
| **Dependent Variable** | CD4 |
| **Covariance Structure** | Unstructured |
| **Subject Effect** | SUBJID |
| **Estimation Method** | REML |
| **Residual Variance Method** | Profile |
| **Fixed Effects SE Method** | Model-Based |
| **Degrees of Freedom Method** | Containment |

| **Class Level Information** | | |
| --- | --- | --- |
| **Class** | **Levels** | **Values** |
| **SUBJID** | 177 | 90 91 92 93 94 95 96 97 98 99 100 101 102 103 104 105 106 107 108 109 110 111 112 113 114 115 116 117 118 119 120 121 122 123 124 125 126 127 128 129 130 131 132 133 134 135 136 137 138 139 140 141 142 143 144 145 146 147 148 149 150 151 152 153 154 155 156 157 158 159 160 161 162 163 164 165 166 167 168 169 170 171 172 173 174 175 176 177 1 2 3 4 5 6 7 8 9 10 11 12 13 14 15 16 17 18 19 20 21 22 23 24 25 26 27 28 29 30 31 32 33 34 35 36 37 38 39 40 41 42 43 44 45 46 47 48 49 50 51 52 53 54 55 56 57 58 59 60 61 62 63 64 65 66 67 68 69 70 71 72 73 74 75 76 77 78 79 80 81 82 83 84 85 86 87 88 89 |
| **ARM** | 2 | MOG COG |
| **VISITGR** | 7 | 1 2 3 4 5 6 0 |
| **AGEGR1** | 5 | 40-49 20-29 30-39 50-60 < 20 |
| **RELG** | 2 | Islam Christianity |
| **RACE** | 4 | Hausa / Fulani Yoruba Igbo Other |
| **SEX** | 2 | Female Male |
| **MARSTS** | 4 | Married Single Widowed Divorcedo |
| **EDC** | 5 | None Secondary Quranic Tertiary Primary |
| **OCC** | 5 | Unemployed Trader Artisan Entrepreneur Civil servant |
| **FAMMEM** | 3 | 6-10 10> 2-5 |
| **INGR1** | 3 | Minimum Income and below Above minimum Income Not Indicated |

| **Dimensions** | |
| --- | --- |
| **Covariance Parameters** | 4 |
| **Columns in X** | 39 |
| **Columns in Z per Subject** | 2 |
| **Subjects** | 177 |
| **Max Obs per Subject** | 7 |

| **Number of Observations** | |
| --- | --- |
| **Number of Observations Read** | 1239 |
| **Number of Observations Used** | 1239 |
| **Number of Observations Not Used** | 0 |

| **Iteration History** | | | |
| --- | --- | --- | --- |
| **Iteration** | **Evaluations** | **-2 Res Log Like** | **Criterion** |
| **0** | 1 | 4273.22620142 |  |
| **1** | 2 | 1936.20963049 | 0.00000005 |
| **2** | 1 | 1936.20962388 | 0.00000000 |

| Convergence criteria met. |
| --- |

| **Covariance Parameter Estimates** | | | | | |
| --- | --- | --- | --- | --- | --- |
| **Cov Parm** | **Subject** | **Estimate** | **Standard Error** | **Z Value** | **Pr Z** |
| **UN(1,1)** | SUBJID | 1.4451 | 0.1720 | 8.40 | <.0001 |
| **UN(2,1)** | SUBJID | 0.01519 | 0.01776 | 0.86 | 0.3925 |
| **UN(2,2)** | SUBJID | 0.02732 | 0.003334 | 8.19 | <.0001 |
| **Residual** |  | 0.1070 | 0.005087 | 21.04 | <.0001 |

| **Fit Statistics** | |
| --- | --- |
| **-2 Res Log Likelihood** | 1936.2 |
| **AIC (Smaller is Better)** | 1944.2 |
| **AICC (Smaller is Better)** | 1944.2 |
| **BIC (Smaller is Better)** | 1956.9 |

| **Null Model Likelihood Ratio Test** | | |
| --- | --- | --- |
| **DF** | **Chi-Square** | **Pr > ChiSq** |
| 3 | 2337.02 | <.0001 |

| **Solution for Fixed Effects** | | | | | | | | | |
| --- | --- | --- | --- | --- | --- | --- | --- | --- | --- |
| **Effect** | **Planned Arm** | **Age Group** | **Religion** | **Race** | **Sex** | **Marital Status** | **Education** | **Occupation** | **Number of Family Members** |
| **Intercept** |  |  |  |  |  |  |  |  |  |
| **ARM** | MOG |  |  |  |  |  |  |  |  |
| **ARM** | COG |  |  |  |  |  |  |  |  |
| **VISIT** |  |  |  |  |  |  |  |  |  |
| **VISIT*ARM** | MOG |  |  |  |  |  |  |  |  |
| **VISIT*ARM** | COG |  |  |  |  |  |  |  |  |
| **RACE** |  |  |  | Hausa / Fulani |  |  |  |  |  |
| **RACE** |  |  |  | Yoruba |  |  |  |  |  |
| **RACE** |  |  |  | Igbo |  |  |  |  |  |
| **RACE** |  |  |  | Other |  |  |  |  |  |
| **FAMMEM** |  |  |  |  |  |  |  |  | 6-10 |
| **FAMMEM** |  |  |  |  |  |  |  |  | 10> |
| **FAMMEM** |  |  |  |  |  |  |  |  | 2-5 |
| **INGR1** |  |  |  |  |  |  |  |  |  |
| **INGR1** |  |  |  |  |  |  |  |  |  |
| **INGR1** |  |  |  |  |  |  |  |  |  |
| **SEX** |  |  |  |  | Female |  |  |  |  |
| **SEX** |  |  |  |  | Male |  |  |  |  |
| **AGEGR1** |  | 40-49 |  |  |  |  |  |  |  |
| **AGEGR1** |  | 20-29 |  |  |  |  |  |  |  |
| **AGEGR1** |  | 30-39 |  |  |  |  |  |  |  |
| **AGEGR1** |  | 50-60 |  |  |  |  |  |  |  |
| **AGEGR1** |  | < 20 |  |  |  |  |  |  |  |
| **RELG** |  |  | Islam |  |  |  |  |  |  |
| **RELG** |  |  | Christianity |  |  |  |  |  |  |
| **MARSTS** |  |  |  |  |  | Married |  |  |  |
| **MARSTS** |  |  |  |  |  | Single |  |  |  |
| **MARSTS** |  |  |  |  |  | Widowed |  |  |  |
| **MARSTS** |  |  |  |  |  | Divorcedo |  |  |  |
| **EDC** |  |  |  |  |  |  | None |  |  |
| **EDC** |  |  |  |  |  |  | Secondary |  |  |
| **EDC** |  |  |  |  |  |  | Quranic |  |  |
| **EDC** |  |  |  |  |  |  | Tertiary |  |  |
| **EDC** |  |  |  |  |  |  | Primary |  |  |
| **OCC** |  |  |  |  |  |  |  | Unemployed |  |
| **OCC** |  |  |  |  |  |  |  | Trader |  |
| **OCC** |  |  |  |  |  |  |  | Artisan |  |
| **OCC** |  |  |  |  |  |  |  | Entrepreneur |  |
| **OCC** |  |  |  |  |  |  |  | Civil servant |  |

| **Solution for Fixed Effects** | | | | | | | | | |
| --- | --- | --- | --- | --- | --- | --- | --- | --- | --- |
| **Effect** | **Planned Arm** | **Age Group** | **Religion** | **Race** | **Sex** | **Marital Status** | **Education** | **Occupation** | **Annual Income Group** |
| **Intercept** |  |  |  |  |  |  |  |  |  |
| **ARM** | MOG |  |  |  |  |  |  |  |  |
| **ARM** | COG |  |  |  |  |  |  |  |  |
| **VISIT** |  |  |  |  |  |  |  |  |  |
| **VISIT*ARM** | MOG |  |  |  |  |  |  |  |  |
| **VISIT*ARM** | COG |  |  |  |  |  |  |  |  |
| **RACE** |  |  |  | Hausa / Fulani |  |  |  |  |  |
| **RACE** |  |  |  | Yoruba |  |  |  |  |  |
| **RACE** |  |  |  | Igbo |  |  |  |  |  |
| **RACE** |  |  |  | Other |  |  |  |  |  |
| **FAMMEM** |  |  |  |  |  |  |  |  |  |
| **FAMMEM** |  |  |  |  |  |  |  |  |  |
| **FAMMEM** |  |  |  |  |  |  |  |  |  |
| **INGR1** |  |  |  |  |  |  |  |  | Minimum Income and below |
| **INGR1** |  |  |  |  |  |  |  |  | Above minimum Income |
| **INGR1** |  |  |  |  |  |  |  |  | Not Indicated |
| **SEX** |  |  |  |  | Female |  |  |  |  |
| **SEX** |  |  |  |  | Male |  |  |  |  |
| **AGEGR1** |  | 40-49 |  |  |  |  |  |  |  |
| **AGEGR1** |  | 20-29 |  |  |  |  |  |  |  |
| **AGEGR1** |  | 30-39 |  |  |  |  |  |  |  |
| **AGEGR1** |  | 50-60 |  |  |  |  |  |  |  |
| **AGEGR1** |  | < 20 |  |  |  |  |  |  |  |
| **RELG** |  |  | Islam |  |  |  |  |  |  |
| **RELG** |  |  | Christianity |  |  |  |  |  |  |
| **MARSTS** |  |  |  |  |  | Married |  |  |  |
| **MARSTS** |  |  |  |  |  | Single |  |  |  |
| **MARSTS** |  |  |  |  |  | Widowed |  |  |  |
| **MARSTS** |  |  |  |  |  | Divorcedo |  |  |  |
| **EDC** |  |  |  |  |  |  | None |  |  |
| **EDC** |  |  |  |  |  |  | Secondary |  |  |
| **EDC** |  |  |  |  |  |  | Quranic |  |  |
| **EDC** |  |  |  |  |  |  | Tertiary |  |  |
| **EDC** |  |  |  |  |  |  | Primary |  |  |
| **OCC** |  |  |  |  |  |  |  | Unemployed |  |
| **OCC** |  |  |  |  |  |  |  | Trader |  |
| **OCC** |  |  |  |  |  |  |  | Artisan |  |
| **OCC** |  |  |  |  |  |  |  | Entrepreneur |  |
| **OCC** |  |  |  |  |  |  |  | Civil servant |  |

| **Solution for Fixed Effects** | | | | | | | | | |
| --- | --- | --- | --- | --- | --- | --- | --- | --- | --- |
| **Effect** | **Planned Arm** | **Age Group** | **Religion** | **Race** | **Sex** | **Marital Status** | **Education** | **Occupation** | **Estimate** |
| **Intercept** |  |  |  |  |  |  |  |  | 2.7973 |
| **ARM** | MOG |  |  |  |  |  |  |  | -0.03321 |
| **ARM** | COG |  |  |  |  |  |  |  | 0 |
| **VISIT** |  |  |  |  |  |  |  |  | 0.03355 |
| **VISIT*ARM** | MOG |  |  |  |  |  |  |  | 0.1033 |
| **VISIT*ARM** | COG |  |  |  |  |  |  |  | 0 |
| **RACE** |  |  |  | Hausa / Fulani |  |  |  |  | 0.04884 |
| **RACE** |  |  |  | Yoruba |  |  |  |  | -0.3776 |
| **RACE** |  |  |  | Igbo |  |  |  |  | 0.6599 |
| **RACE** |  |  |  | Other |  |  |  |  | 0 |
| **FAMMEM** |  |  |  |  |  |  |  |  | 0.4903 |
| **FAMMEM** |  |  |  |  |  |  |  |  | 0.5146 |
| **FAMMEM** |  |  |  |  |  |  |  |  | 0 |
| **INGR1** |  |  |  |  |  |  |  |  | 0.1183 |
| **INGR1** |  |  |  |  |  |  |  |  | 0.6181 |
| **INGR1** |  |  |  |  |  |  |  |  | 0 |
| **SEX** |  |  |  |  | Female |  |  |  | 0.1641 |
| **SEX** |  |  |  |  | Male |  |  |  | 0 |
| **AGEGR1** |  | 40-49 |  |  |  |  |  |  | 0.2343 |
| **AGEGR1** |  | 20-29 |  |  |  |  |  |  | 0.08141 |
| **AGEGR1** |  | 30-39 |  |  |  |  |  |  | -0.03575 |
| **AGEGR1** |  | 50-60 |  |  |  |  |  |  | -0.2085 |
| **AGEGR1** |  | < 20 |  |  |  |  |  |  | 0 |
| **RELG** |  |  | Islam |  |  |  |  |  | -0.09587 |
| **RELG** |  |  | Christianity |  |  |  |  |  | 0 |
| **MARSTS** |  |  |  |  |  | Married |  |  | -0.04391 |
| **MARSTS** |  |  |  |  |  | Single |  |  | 0.1291 |
| **MARSTS** |  |  |  |  |  | Widowed |  |  | 0.07399 |
| **MARSTS** |  |  |  |  |  | Divorcedo |  |  | 0 |
| **EDC** |  |  |  |  |  |  | None |  | 0.04308 |
| **EDC** |  |  |  |  |  |  | Secondary |  | -0.03589 |
| **EDC** |  |  |  |  |  |  | Quranic |  | -0.5790 |
| **EDC** |  |  |  |  |  |  | Tertiary |  | -0.03134 |
| **EDC** |  |  |  |  |  |  | Primary |  | 0 |
| **OCC** |  |  |  |  |  |  |  | Unemployed | 0.3477 |
| **OCC** |  |  |  |  |  |  |  | Trader | 0.07638 |
| **OCC** |  |  |  |  |  |  |  | Artisan | 0.2262 |
| **OCC** |  |  |  |  |  |  |  | Entrepreneur | 0.2466 |
| **OCC** |  |  |  |  |  |  |  | Civil servant | 0 |

| **Solution for Fixed Effects** | | | | | | | | | | |
| --- | --- | --- | --- | --- | --- | --- | --- | --- | --- | --- |
| **Effect** | **Planned Arm** | **Age Group** | **Religion** | **Race** | **Sex** | **Marital Status** | **Education** | **Occupation** | **Standard Error** | **DF** |
| **Intercept** |  |  |  |  |  |  |  |  | 0.9600 | 151 |
| **ARM** | MOG |  |  |  |  |  |  |  | 0.1915 | 885 |
| **ARM** | COG |  |  |  |  |  |  |  | . | . |
| **VISIT** |  |  |  |  |  |  |  |  | 0.01881 | 175 |
| **VISIT*ARM** | MOG |  |  |  |  |  |  |  | 0.02653 | 885 |
| **VISIT*ARM** | COG |  |  |  |  |  |  |  | . | . |
| **RACE** |  |  |  | Hausa / Fulani |  |  |  |  | 0.3080 | 885 |
| **RACE** |  |  |  | Yoruba |  |  |  |  | 0.3651 | 885 |
| **RACE** |  |  |  | Igbo |  |  |  |  | 0.3870 | 885 |
| **RACE** |  |  |  | Other |  |  |  |  | . | . |
| **FAMMEM** |  |  |  |  |  |  |  |  | 0.2444 | 885 |
| **FAMMEM** |  |  |  |  |  |  |  |  | 0.2393 | 885 |
| **FAMMEM** |  |  |  |  |  |  |  |  | . | . |
| **INGR1** |  |  |  |  |  |  |  |  | 0.3544 | 885 |
| **INGR1** |  |  |  |  |  |  |  |  | 0.4322 | 885 |
| **INGR1** |  |  |  |  |  |  |  |  | . | . |
| **SEX** |  |  |  |  | Female |  |  |  | 0.2653 | 885 |
| **SEX** |  |  |  |  | Male |  |  |  | . | . |
| **AGEGR1** |  | 40-49 |  |  |  |  |  |  | 0.6993 | 885 |
| **AGEGR1** |  | 20-29 |  |  |  |  |  |  | 0.6821 | 885 |
| **AGEGR1** |  | 30-39 |  |  |  |  |  |  | 0.6688 | 885 |
| **AGEGR1** |  | 50-60 |  |  |  |  |  |  | 0.7569 | 885 |
| **AGEGR1** |  | < 20 |  |  |  |  |  |  | . | . |
| **RELG** |  |  | Islam |  |  |  |  |  | 0.2466 | 885 |
| **RELG** |  |  | Christianity |  |  |  |  |  | . | . |
| **MARSTS** |  |  |  |  |  | Married |  |  | 0.2621 | 885 |
| **MARSTS** |  |  |  |  |  | Single |  |  | 0.3697 | 885 |
| **MARSTS** |  |  |  |  |  | Widowed |  |  | 0.3005 | 885 |
| **MARSTS** |  |  |  |  |  | Divorcedo |  |  | . | . |
| **EDC** |  |  |  |  |  |  | None |  | 0.3679 | 885 |
| **EDC** |  |  |  |  |  |  | Secondary |  | 0.3231 | 885 |
| **EDC** |  |  |  |  |  |  | Quranic |  | 0.3478 | 885 |
| **EDC** |  |  |  |  |  |  | Tertiary |  | 0.3573 | 885 |
| **EDC** |  |  |  |  |  |  | Primary |  | . | . |
| **OCC** |  |  |  |  |  |  |  | Unemployed | 0.3355 | 885 |
| **OCC** |  |  |  |  |  |  |  | Trader | 0.3125 | 885 |
| **OCC** |  |  |  |  |  |  |  | Artisan | 0.3320 | 885 |
| **OCC** |  |  |  |  |  |  |  | Entrepreneur | 0.3626 | 885 |
| **OCC** |  |  |  |  |  |  |  | Civil servant | . | . |

| **Solution for Fixed Effects** | | | | | | | | | | |
| --- | --- | --- | --- | --- | --- | --- | --- | --- | --- | --- |
| **Effect** | **Planned Arm** | **Age Group** | **Religion** | **Race** | **Sex** | **Marital Status** | **Education** | **Occupation** | **t Value** | **Pr > \|t\|** |
| **Intercept** |  |  |  |  |  |  |  |  | 2.91 | 0.0041 |
| **ARM** | MOG |  |  |  |  |  |  |  | -0.17 | 0.8624 |
| **ARM** | COG |  |  |  |  |  |  |  | . | . |
| **VISIT** |  |  |  |  |  |  |  |  | 1.78 | 0.0762 |
| **VISIT*ARM** | MOG |  |  |  |  |  |  |  | 3.89 | 0.0001 |
| **VISIT*ARM** | COG |  |  |  |  |  |  |  | . | . |
| **RACE** |  |  |  | Hausa / Fulani |  |  |  |  | 0.16 | 0.8740 |
| **RACE** |  |  |  | Yoruba |  |  |  |  | -1.03 | 0.3014 |
| **RACE** |  |  |  | Igbo |  |  |  |  | 1.71 | 0.0885 |
| **RACE** |  |  |  | Other |  |  |  |  | . | . |
| **FAMMEM** |  |  |  |  |  |  |  |  | 2.01 | 0.0452 |
| **FAMMEM** |  |  |  |  |  |  |  |  | 2.15 | 0.0318 |
| **FAMMEM** |  |  |  |  |  |  |  |  | . | . |
| **INGR1** |  |  |  |  |  |  |  |  | 0.33 | 0.7387 |
| **INGR1** |  |  |  |  |  |  |  |  | 1.43 | 0.1530 |
| **INGR1** |  |  |  |  |  |  |  |  | . | . |
| **SEX** |  |  |  |  | Female |  |  |  | 0.62 | 0.5364 |
| **SEX** |  |  |  |  | Male |  |  |  | . | . |
| **AGEGR1** |  | 40-49 |  |  |  |  |  |  | 0.34 | 0.7377 |
| **AGEGR1** |  | 20-29 |  |  |  |  |  |  | 0.12 | 0.9050 |
| **AGEGR1** |  | 30-39 |  |  |  |  |  |  | -0.05 | 0.9574 |
| **AGEGR1** |  | 50-60 |  |  |  |  |  |  | -0.28 | 0.7830 |
| **AGEGR1** |  | < 20 |  |  |  |  |  |  | . | . |
| **RELG** |  |  | Islam |  |  |  |  |  | -0.39 | 0.6975 |
| **RELG** |  |  | Christianity |  |  |  |  |  | . | . |
| **MARSTS** |  |  |  |  |  | Married |  |  | -0.17 | 0.8670 |
| **MARSTS** |  |  |  |  |  | Single |  |  | 0.35 | 0.7271 |
| **MARSTS** |  |  |  |  |  | Widowed |  |  | 0.25 | 0.8056 |
| **MARSTS** |  |  |  |  |  | Divorcedo |  |  | . | . |
| **EDC** |  |  |  |  |  |  | None |  | 0.12 | 0.9068 |
| **EDC** |  |  |  |  |  |  | Secondary |  | -0.11 | 0.9116 |
| **EDC** |  |  |  |  |  |  | Quranic |  | -1.66 | 0.0964 |
| **EDC** |  |  |  |  |  |  | Tertiary |  | -0.09 | 0.9301 |
| **EDC** |  |  |  |  |  |  | Primary |  | . | . |
| **OCC** |  |  |  |  |  |  |  | Unemployed | 1.04 | 0.3004 |
| **OCC** |  |  |  |  |  |  |  | Trader | 0.24 | 0.8070 |
| **OCC** |  |  |  |  |  |  |  | Artisan | 0.68 | 0.4958 |
| **OCC** |  |  |  |  |  |  |  | Entrepreneur | 0.68 | 0.4967 |
| **OCC** |  |  |  |  |  |  |  | Civil servant | . | . |

| **Type 3 Tests of Fixed Effects** | | | | |
| --- | --- | --- | --- | --- |
| **Effect** | **Num DF** | **Den DF** | **F Value** | **Pr > F** |
| **ARM** | 1 | 885 | 0.03 | 0.8624 |
| **VISIT** | 1 | 175 | 41.25 | <.0001 |
| **VISIT*ARM** | 1 | 885 | 15.16 | 0.0001 |
| **RACE** | 3 | 885 | 2.63 | 0.0491 |
| **FAMMEM** | 2 | 885 | 3.04 | 0.0483 |
| **INGR1** | 2 | 885 | 1.59 | 0.2042 |
| **SEX** | 1 | 885 | 0.38 | 0.5364 |
| **AGEGR1** | 4 | 885 | 0.41 | 0.8036 |
| **RELG** | 1 | 885 | 0.15 | 0.6975 |
| **MARSTS** | 3 | 885 | 0.13 | 0.9394 |
| **EDC** | 4 | 885 | 1.09 | 0.3605 |
| **OCC** | 4 | 885 | 0.36 | 0.8342 |

| **Estimates** | | | | | | | | |
| --- | --- | --- | --- | --- | --- | --- | --- | --- |
| **Label** | **Estimate** | **Standard Error** | **DF** | **t Value** | **Pr > \|t\|** | **Alpha** | **Lower** | **Upper** |
| **Treatment effect overtime** | 0.1033 | 0.02653 | 885 | 3.89 | 0.0001 | 0.05 | 0.05122 | 0.1554 |

| **Least Squares Means** | | | | | | |
| --- | --- | --- | --- | --- | --- | --- |
| **Effect** | **Planned Arm** | **Estimate** | **Standard Error** | **DF** | **t Value** | **Pr > \|t\|** |
| **ARM** | MOG | 3.9847 | 0.2513 | 885 | 15.86 | <.0001 |
| **ARM** | COG | 3.7080 | 0.2564 | 885 | 14.46 | <.0001 |

| **Differences of Least Squares Means** | | | | | | | | | |
| --- | --- | --- | --- | --- | --- | --- | --- | --- | --- |
| **Effect** | **Planned Arm** | **Planned Arm** | **Estimate** | **Standard Error** | **DF** | **t Value** | **Pr > \|t\|** | **Adjustment** | **Adj P** |
| **ARM** | MOG | COG | 0.2767 | 0.2086 | 885 | 1.33 | 0.1850 | Bonferroni | 0.1850 |

| **Model Information** | |
| --- | --- |
| **Data Set** | DATAPATH.ADMOLCRT |
| **Dependent Variable** | BMI |
| **Covariance Structure** | Unstructured |
| **Subject Effect** | SUBJID |
| **Estimation Method** | REML |
| **Residual Variance Method** | Profile |
| **Fixed Effects SE Method** | Model-Based |
| **Degrees of Freedom Method** | Containment |

| **Class Level Information** | | |
| --- | --- | --- |
| **Class** | **Levels** | **Values** |
| **SUBJID** | 177 | 90 91 92 93 94 95 96 97 98 99 100 101 102 103 104 105 106 107 108 109 110 111 112 113 114 115 116 117 118 119 120 121 122 123 124 125 126 127 128 129 130 131 132 133 134 135 136 137 138 139 140 141 142 143 144 145 146 147 148 149 150 151 152 153 154 155 156 157 158 159 160 161 162 163 164 165 166 167 168 169 170 171 172 173 174 175 176 177 1 2 3 4 5 6 7 8 9 10 11 12 13 14 15 16 17 18 19 20 21 22 23 24 25 26 27 28 29 30 31 32 33 34 35 36 37 38 39 40 41 42 43 44 45 46 47 48 49 50 51 52 53 54 55 56 57 58 59 60 61 62 63 64 65 66 67 68 69 70 71 72 73 74 75 76 77 78 79 80 81 82 83 84 85 86 87 88 89 |
| **ARM** | 2 | MOG COG |
| **VISITGR** | 7 | 1 2 3 4 5 6 0 |

| **Dimensions** | |
| --- | --- |
| **Covariance Parameters** | 4 |
| **Columns in X** | 6 |
| **Columns in Z per Subject** | 2 |
| **Subjects** | 177 |
| **Max Obs per Subject** | 7 |

| **Number of Observations** | |
| --- | --- |
| **Number of Observations Read** | 1239 |
| **Number of Observations Used** | 1239 |
| **Number of Observations Not Used** | 0 |

| **Iteration History** | | | |
| --- | --- | --- | --- |
| **Iteration** | **Evaluations** | **-2 Res Log Like** | **Criterion** |
| **0** | 1 | 7217.82513929 |  |
| **1** | 1 | 2869.79086652 | 0.00000000 |

| Convergence criteria met. |
| --- |

| **Covariance Parameter Estimates** | | | | | |
| --- | --- | --- | --- | --- | --- |
| **Cov Parm** | **Subject** | **Estimate** | **Standard Error** | **Z Value** | **Pr Z** |
| **UN(1,1)** | SUBJID | 18.9986 | 2.0396 | 9.31 | <.0001 |
| **UN(2,1)** | SUBJID | 0.04439 | 0.06844 | 0.65 | 0.5166 |
| **UN(2,2)** | SUBJID | 0.03674 | 0.004598 | 7.99 | <.0001 |
| **Residual** |  | 0.1731 | 0.008230 | 21.04 | <.0001 |

| **Fit Statistics** | |
| --- | --- |
| **-2 Res Log Likelihood** | 2869.8 |
| **AIC (Smaller is Better)** | 2877.8 |
| **AICC (Smaller is Better)** | 2877.8 |
| **BIC (Smaller is Better)** | 2890.5 |

| **Null Model Likelihood Ratio Test** | | |
| --- | --- | --- |
| **DF** | **Chi-Square** | **Pr > ChiSq** |
| 3 | 4348.03 | <.0001 |

| **Solution for Fixed Effects** | | | | | | |
| --- | --- | --- | --- | --- | --- | --- |
| **Effect** | **Planned Arm** | **Estimate** | **Standard Error** | **DF** | **t Value** | **Pr > \|t\|** |
| **Intercept** |  | 23.7353 | 0.4656 | 175 | 50.98 | <.0001 |
| **ARM** | MOG | 1.1028 | 0.6566 | 885 | 1.68 | 0.0934 |
| **ARM** | COG | 0 | . | . | . | . |
| **VISIT** |  | 0.07769 | 0.02208 | 175 | 3.52 | 0.0006 |
| **VISIT*ARM** | MOG | -0.02031 | 0.03114 | 885 | -0.65 | 0.5145 |
| **VISIT*ARM** | COG | 0 | . | . | . | . |

| **Type 3 Tests of Fixed Effects** | | | | |
| --- | --- | --- | --- | --- |
| **Effect** | **Num DF** | **Den DF** | **F Value** | **Pr > F** |
| **ARM** | 1 | 885 | 2.82 | 0.0934 |
| **VISIT** | 1 | 175 | 18.81 | <.0001 |
| **VISIT*ARM** | 1 | 885 | 0.43 | 0.5145 |

| **Estimates** | | | | | | | | |
| --- | --- | --- | --- | --- | --- | --- | --- | --- |
| **Label** | **Estimate** | **Standard Error** | **DF** | **t Value** | **Pr > \|t\|** | **Alpha** | **Lower** | **Upper** |
| **Treatment effect overtime** | -0.02031 | 0.03114 | 885 | -0.65 | 0.5145 | 0.05 | -0.08144 | 0.04081 |

| **Least Squares Means** | | | | | | |
| --- | --- | --- | --- | --- | --- | --- |
| **Effect** | **Planned Arm** | **Estimate** | **Standard Error** | **DF** | **t Value** | **Pr > \|t\|** |
| **ARM** | MOG | 25.0102 | 0.4695 | 885 | 53.27 | <.0001 |
| **ARM** | COG | 23.9684 | 0.4722 | 885 | 50.76 | <.0001 |

| **Differences of Least Squares Means** | | | | | | | |
| --- | --- | --- | --- | --- | --- | --- | --- |
| **Effect** | **Planned Arm** | **Planned Arm** | **Estimate** | **Standard Error** | **DF** | **t Value** | **Pr > \|t\|** |
| **ARM** | MOG | COG | 1.0418 | 0.6659 | 885 | 1.56 | 0.1180 |

| **Model Information** | |
| --- | --- |
| **Data Set** | DATAPATH.ADMOLCRT |
| **Dependent Variable** | BMI |
| **Covariance Structure** | Unstructured |
| **Subject Effect** | SUBJID |
| **Estimation Method** | REML |
| **Residual Variance Method** | Profile |
| **Fixed Effects SE Method** | Model-Based |
| **Degrees of Freedom Method** | Containment |

| **Class Level Information** | | |
| --- | --- | --- |
| **Class** | **Levels** | **Values** |
| **SUBJID** | 177 | 90 91 92 93 94 95 96 97 98 99 100 101 102 103 104 105 106 107 108 109 110 111 112 113 114 115 116 117 118 119 120 121 122 123 124 125 126 127 128 129 130 131 132 133 134 135 136 137 138 139 140 141 142 143 144 145 146 147 148 149 150 151 152 153 154 155 156 157 158 159 160 161 162 163 164 165 166 167 168 169 170 171 172 173 174 175 176 177 1 2 3 4 5 6 7 8 9 10 11 12 13 14 15 16 17 18 19 20 21 22 23 24 25 26 27 28 29 30 31 32 33 34 35 36 37 38 39 40 41 42 43 44 45 46 47 48 49 50 51 52 53 54 55 56 57 58 59 60 61 62 63 64 65 66 67 68 69 70 71 72 73 74 75 76 77 78 79 80 81 82 83 84 85 86 87 88 89 |
| **ARM** | 2 | MOG COG |
| **VISITGR** | 7 | 1 2 3 4 5 6 0 |
| **AGEGR1** | 5 | 40-49 20-29 30-39 50-60 < 20 |
| **RELG** | 2 | Islam Christianity |
| **RACE** | 4 | Hausa / Fulani Yoruba Igbo Other |
| **SEX** | 2 | Female Male |
| **MARSTS** | 4 | Married Single Widowed Divorcedo |
| **EDC** | 5 | None Secondary Quranic Tertiary Primary |
| **OCC** | 5 | Unemployed Trader Artisan Entrepreneur Civil servant |
| **FAMMEM** | 3 | 6-10 10> 2-5 |
| **INGR1** | 3 | Minimum Income and below Above minimum Income Not Indicated |

| **Dimensions** | |
| --- | --- |
| **Covariance Parameters** | 4 |
| **Columns in X** | 39 |
| **Columns in Z per Subject** | 2 |
| **Subjects** | 177 |
| **Max Obs per Subject** | 7 |

| **Number of Observations** | |
| --- | --- |
| **Number of Observations Read** | 1239 |
| **Number of Observations Used** | 1239 |
| **Number of Observations Not Used** | 0 |

| **Iteration History** | | | |
| --- | --- | --- | --- |
| **Iteration** | **Evaluations** | **-2 Res Log Like** | **Criterion** |
| **0** | 1 | 7091.23340641 |  |
| **1** | 2 | 2806.75341962 | 0.00000991 |
| **2** | 1 | 2806.75054485 | 0.00000000 |

| Convergence criteria met. |
| --- |

| **Covariance Parameter Estimates** | | | | | |
| --- | --- | --- | --- | --- | --- |
| **Cov Parm** | **Subject** | **Estimate** | **Standard Error** | **Z Value** | **Pr Z** |
| **UN(1,1)** | SUBJID | 19.6008 | 2.2654 | 8.65 | <.0001 |
| **UN(2,1)** | SUBJID | 0.08086 | 0.07656 | 1.06 | 0.2908 |
| **UN(2,2)** | SUBJID | 0.03674 | 0.004597 | 7.99 | <.0001 |
| **Residual** |  | 0.1731 | 0.008230 | 21.04 | <.0001 |

| **Fit Statistics** | |
| --- | --- |
| **-2 Res Log Likelihood** | 2806.8 |
| **AIC (Smaller is Better)** | 2814.8 |
| **AICC (Smaller is Better)** | 2814.8 |
| **BIC (Smaller is Better)** | 2827.5 |

| **Null Model Likelihood Ratio Test** | | |
| --- | --- | --- |
| **DF** | **Chi-Square** | **Pr > ChiSq** |
| 3 | 4284.48 | <.0001 |

| **Solution for Fixed Effects** | | | | | | | | | |
| --- | --- | --- | --- | --- | --- | --- | --- | --- | --- |
| **Effect** | **Planned Arm** | **Age Group** | **Religion** | **Race** | **Sex** | **Marital Status** | **Education** | **Occupation** | **Number of Family Members** |
| **Intercept** |  |  |  |  |  |  |  |  |  |
| **ARM** | MOG |  |  |  |  |  |  |  |  |
| **ARM** | COG |  |  |  |  |  |  |  |  |
| **VISIT** |  |  |  |  |  |  |  |  |  |
| **VISIT*ARM** | MOG |  |  |  |  |  |  |  |  |
| **VISIT*ARM** | COG |  |  |  |  |  |  |  |  |
| **RACE** |  |  |  | Hausa / Fulani |  |  |  |  |  |
| **RACE** |  |  |  | Yoruba |  |  |  |  |  |
| **RACE** |  |  |  | Igbo |  |  |  |  |  |
| **RACE** |  |  |  | Other |  |  |  |  |  |
| **FAMMEM** |  |  |  |  |  |  |  |  | 6-10 |
| **FAMMEM** |  |  |  |  |  |  |  |  | 10> |
| **FAMMEM** |  |  |  |  |  |  |  |  | 2-5 |
| **INGR1** |  |  |  |  |  |  |  |  |  |
| **INGR1** |  |  |  |  |  |  |  |  |  |
| **INGR1** |  |  |  |  |  |  |  |  |  |
| **SEX** |  |  |  |  | Female |  |  |  |  |
| **SEX** |  |  |  |  | Male |  |  |  |  |
| **AGEGR1** |  | 40-49 |  |  |  |  |  |  |  |
| **AGEGR1** |  | 20-29 |  |  |  |  |  |  |  |
| **AGEGR1** |  | 30-39 |  |  |  |  |  |  |  |
| **AGEGR1** |  | 50-60 |  |  |  |  |  |  |  |
| **AGEGR1** |  | < 20 |  |  |  |  |  |  |  |
| **RELG** |  |  | Islam |  |  |  |  |  |  |
| **RELG** |  |  | Christianity |  |  |  |  |  |  |
| **MARSTS** |  |  |  |  |  | Married |  |  |  |
| **MARSTS** |  |  |  |  |  | Single |  |  |  |
| **MARSTS** |  |  |  |  |  | Widowed |  |  |  |
| **MARSTS** |  |  |  |  |  | Divorcedo |  |  |  |
| **EDC** |  |  |  |  |  |  | None |  |  |
| **EDC** |  |  |  |  |  |  | Secondary |  |  |
| **EDC** |  |  |  |  |  |  | Quranic |  |  |
| **EDC** |  |  |  |  |  |  | Tertiary |  |  |
| **EDC** |  |  |  |  |  |  | Primary |  |  |
| **OCC** |  |  |  |  |  |  |  | Unemployed |  |
| **OCC** |  |  |  |  |  |  |  | Trader |  |
| **OCC** |  |  |  |  |  |  |  | Artisan |  |
| **OCC** |  |  |  |  |  |  |  | Entrepreneur |  |
| **OCC** |  |  |  |  |  |  |  | Civil servant |  |

| **Solution for Fixed Effects** | | | | | | | | | |
| --- | --- | --- | --- | --- | --- | --- | --- | --- | --- |
| **Effect** | **Planned Arm** | **Age Group** | **Religion** | **Race** | **Sex** | **Marital Status** | **Education** | **Occupation** | **Annual Income Group** |
| **Intercept** |  |  |  |  |  |  |  |  |  |
| **ARM** | MOG |  |  |  |  |  |  |  |  |
| **ARM** | COG |  |  |  |  |  |  |  |  |
| **VISIT** |  |  |  |  |  |  |  |  |  |
| **VISIT*ARM** | MOG |  |  |  |  |  |  |  |  |
| **VISIT*ARM** | COG |  |  |  |  |  |  |  |  |
| **RACE** |  |  |  | Hausa / Fulani |  |  |  |  |  |
| **RACE** |  |  |  | Yoruba |  |  |  |  |  |
| **RACE** |  |  |  | Igbo |  |  |  |  |  |
| **RACE** |  |  |  | Other |  |  |  |  |  |
| **FAMMEM** |  |  |  |  |  |  |  |  |  |
| **FAMMEM** |  |  |  |  |  |  |  |  |  |
| **FAMMEM** |  |  |  |  |  |  |  |  |  |
| **INGR1** |  |  |  |  |  |  |  |  | Minimum Income and below |
| **INGR1** |  |  |  |  |  |  |  |  | Above minimum Income |
| **INGR1** |  |  |  |  |  |  |  |  | Not Indicated |
| **SEX** |  |  |  |  | Female |  |  |  |  |
| **SEX** |  |  |  |  | Male |  |  |  |  |
| **AGEGR1** |  | 40-49 |  |  |  |  |  |  |  |
| **AGEGR1** |  | 20-29 |  |  |  |  |  |  |  |
| **AGEGR1** |  | 30-39 |  |  |  |  |  |  |  |
| **AGEGR1** |  | 50-60 |  |  |  |  |  |  |  |
| **AGEGR1** |  | < 20 |  |  |  |  |  |  |  |
| **RELG** |  |  | Islam |  |  |  |  |  |  |
| **RELG** |  |  | Christianity |  |  |  |  |  |  |
| **MARSTS** |  |  |  |  |  | Married |  |  |  |
| **MARSTS** |  |  |  |  |  | Single |  |  |  |
| **MARSTS** |  |  |  |  |  | Widowed |  |  |  |
| **MARSTS** |  |  |  |  |  | Divorcedo |  |  |  |
| **EDC** |  |  |  |  |  |  | None |  |  |
| **EDC** |  |  |  |  |  |  | Secondary |  |  |
| **EDC** |  |  |  |  |  |  | Quranic |  |  |
| **EDC** |  |  |  |  |  |  | Tertiary |  |  |
| **EDC** |  |  |  |  |  |  | Primary |  |  |
| **OCC** |  |  |  |  |  |  |  | Unemployed |  |
| **OCC** |  |  |  |  |  |  |  | Trader |  |
| **OCC** |  |  |  |  |  |  |  | Artisan |  |
| **OCC** |  |  |  |  |  |  |  | Entrepreneur |  |
| **OCC** |  |  |  |  |  |  |  | Civil servant |  |

| **Solution for Fixed Effects** | | | | | | | | | |
| --- | --- | --- | --- | --- | --- | --- | --- | --- | --- |
| **Effect** | **Planned Arm** | **Age Group** | **Religion** | **Race** | **Sex** | **Marital Status** | **Education** | **Occupation** | **Estimate** |
| **Intercept** |  |  |  |  |  |  |  |  | 24.7915 |
| **ARM** | MOG |  |  |  |  |  |  |  | 1.4131 |
| **ARM** | COG |  |  |  |  |  |  |  | 0 |
| **VISIT** |  |  |  |  |  |  |  |  | 0.07769 |
| **VISIT*ARM** | MOG |  |  |  |  |  |  |  | -0.02031 |
| **VISIT*ARM** | COG |  |  |  |  |  |  |  | 0 |
| **RACE** |  |  |  | Hausa / Fulani |  |  |  |  | -2.0525 |
| **RACE** |  |  |  | Yoruba |  |  |  |  | -1.1934 |
| **RACE** |  |  |  | Igbo |  |  |  |  | -0.7318 |
| **RACE** |  |  |  | Other |  |  |  |  | 0 |
| **FAMMEM** |  |  |  |  |  |  |  |  | 0.6850 |
| **FAMMEM** |  |  |  |  |  |  |  |  | 0.07249 |
| **FAMMEM** |  |  |  |  |  |  |  |  | 0 |
| **INGR1** |  |  |  |  |  |  |  |  | 0.3696 |
| **INGR1** |  |  |  |  |  |  |  |  | 1.1220 |
| **INGR1** |  |  |  |  |  |  |  |  | 0 |
| **SEX** |  |  |  |  | Female |  |  |  | -0.7862 |
| **SEX** |  |  |  |  | Male |  |  |  | 0 |
| **AGEGR1** |  | 40-49 |  |  |  |  |  |  | 1.8700 |
| **AGEGR1** |  | 20-29 |  |  |  |  |  |  | 1.1080 |
| **AGEGR1** |  | 30-39 |  |  |  |  |  |  | 1.8162 |
| **AGEGR1** |  | 50-60 |  |  |  |  |  |  | 0.4024 |
| **AGEGR1** |  | < 20 |  |  |  |  |  |  | 0 |
| **RELG** |  |  | Islam |  |  |  |  |  | -0.09295 |
| **RELG** |  |  | Christianity |  |  |  |  |  | 0 |
| **MARSTS** |  |  |  |  |  | Married |  |  | -1.0045 |
| **MARSTS** |  |  |  |  |  | Single |  |  | -0.8328 |
| **MARSTS** |  |  |  |  |  | Widowed |  |  | -0.1990 |
| **MARSTS** |  |  |  |  |  | Divorcedo |  |  | 0 |
| **EDC** |  |  |  |  |  |  | None |  | -0.4988 |
| **EDC** |  |  |  |  |  |  | Secondary |  | -1.5060 |
| **EDC** |  |  |  |  |  |  | Quranic |  | -1.2220 |
| **EDC** |  |  |  |  |  |  | Tertiary |  | 0.7404 |
| **EDC** |  |  |  |  |  |  | Primary |  | 0 |
| **OCC** |  |  |  |  |  |  |  | Unemployed | -0.3909 |
| **OCC** |  |  |  |  |  |  |  | Trader | 0.3517 |
| **OCC** |  |  |  |  |  |  |  | Artisan | 0.1828 |
| **OCC** |  |  |  |  |  |  |  | Entrepreneur | -1.0607 |
| **OCC** |  |  |  |  |  |  |  | Civil servant | 0 |

| **Solution for Fixed Effects** | | | | | | | | | | |
| --- | --- | --- | --- | --- | --- | --- | --- | --- | --- | --- |
| **Effect** | **Planned Arm** | **Age Group** | **Religion** | **Race** | **Sex** | **Marital Status** | **Education** | **Occupation** | **Standard Error** | **DF** |
| **Intercept** |  |  |  |  |  |  |  |  | 3.4763 | 151 |
| **ARM** | MOG |  |  |  |  |  |  |  | 0.6946 | 885 |
| **ARM** | COG |  |  |  |  |  |  |  | . | . |
| **VISIT** |  |  |  |  |  |  |  |  | 0.02208 | 175 |
| **VISIT*ARM** | MOG |  |  |  |  |  |  |  | 0.03114 | 885 |
| **VISIT*ARM** | COG |  |  |  |  |  |  |  | . | . |
| **RACE** |  |  |  | Hausa / Fulani |  |  |  |  | 1.1152 | 885 |
| **RACE** |  |  |  | Yoruba |  |  |  |  | 1.3220 | 885 |
| **RACE** |  |  |  | Igbo |  |  |  |  | 1.4014 | 885 |
| **RACE** |  |  |  | Other |  |  |  |  | . | . |
| **FAMMEM** |  |  |  |  |  |  |  |  | 0.8850 | 885 |
| **FAMMEM** |  |  |  |  |  |  |  |  | 0.8665 | 885 |
| **FAMMEM** |  |  |  |  |  |  |  |  | . | . |
| **INGR1** |  |  |  |  |  |  |  |  | 1.2831 | 885 |
| **INGR1** |  |  |  |  |  |  |  |  | 1.5647 | 885 |
| **INGR1** |  |  |  |  |  |  |  |  | . | . |
| **SEX** |  |  |  |  | Female |  |  |  | 0.9605 | 885 |
| **SEX** |  |  |  |  | Male |  |  |  | . | . |
| **AGEGR1** |  | 40-49 |  |  |  |  |  |  | 2.5320 | 885 |
| **AGEGR1** |  | 20-29 |  |  |  |  |  |  | 2.4699 | 885 |
| **AGEGR1** |  | 30-39 |  |  |  |  |  |  | 2.4215 | 885 |
| **AGEGR1** |  | 50-60 |  |  |  |  |  |  | 2.7405 | 885 |
| **AGEGR1** |  | < 20 |  |  |  |  |  |  | . | . |
| **RELG** |  |  | Islam |  |  |  |  |  | 0.8928 | 885 |
| **RELG** |  |  | Christianity |  |  |  |  |  | . | . |
| **MARSTS** |  |  |  |  |  | Married |  |  | 0.9491 | 885 |
| **MARSTS** |  |  |  |  |  | Single |  |  | 1.3385 | 885 |
| **MARSTS** |  |  |  |  |  | Widowed |  |  | 1.0881 | 885 |
| **MARSTS** |  |  |  |  |  | Divorcedo |  |  | . | . |
| **EDC** |  |  |  |  |  |  | None |  | 1.3320 | 885 |
| **EDC** |  |  |  |  |  |  | Secondary |  | 1.1699 | 885 |
| **EDC** |  |  |  |  |  |  | Quranic |  | 1.2595 | 885 |
| **EDC** |  |  |  |  |  |  | Tertiary |  | 1.2937 | 885 |
| **EDC** |  |  |  |  |  |  | Primary |  | . | . |
| **OCC** |  |  |  |  |  |  |  | Unemployed | 1.2148 | 885 |
| **OCC** |  |  |  |  |  |  |  | Trader | 1.1316 | 885 |
| **OCC** |  |  |  |  |  |  |  | Artisan | 1.2021 | 885 |
| **OCC** |  |  |  |  |  |  |  | Entrepreneur | 1.3128 | 885 |
| **OCC** |  |  |  |  |  |  |  | Civil servant | . | . |

| **Solution for Fixed Effects** | | | | | | | | | | |
| --- | --- | --- | --- | --- | --- | --- | --- | --- | --- | --- |
| **Effect** | **Planned Arm** | **Age Group** | **Religion** | **Race** | **Sex** | **Marital Status** | **Education** | **Occupation** | **t Value** | **Pr > \|t\|** |
| **Intercept** |  |  |  |  |  |  |  |  | 7.13 | <.0001 |
| **ARM** | MOG |  |  |  |  |  |  |  | 2.03 | 0.0422 |
| **ARM** | COG |  |  |  |  |  |  |  | . | . |
| **VISIT** |  |  |  |  |  |  |  |  | 3.52 | 0.0006 |
| **VISIT*ARM** | MOG |  |  |  |  |  |  |  | -0.65 | 0.5145 |
| **VISIT*ARM** | COG |  |  |  |  |  |  |  | . | . |
| **RACE** |  |  |  | Hausa / Fulani |  |  |  |  | -1.84 | 0.0660 |
| **RACE** |  |  |  | Yoruba |  |  |  |  | -0.90 | 0.3669 |
| **RACE** |  |  |  | Igbo |  |  |  |  | -0.52 | 0.6017 |
| **RACE** |  |  |  | Other |  |  |  |  | . | . |
| **FAMMEM** |  |  |  |  |  |  |  |  | 0.77 | 0.4391 |
| **FAMMEM** |  |  |  |  |  |  |  |  | 0.08 | 0.9333 |
| **FAMMEM** |  |  |  |  |  |  |  |  | . | . |
| **INGR1** |  |  |  |  |  |  |  |  | 0.29 | 0.7734 |
| **INGR1** |  |  |  |  |  |  |  |  | 0.72 | 0.4735 |
| **INGR1** |  |  |  |  |  |  |  |  | . | . |
| **SEX** |  |  |  |  | Female |  |  |  | -0.82 | 0.4133 |
| **SEX** |  |  |  |  | Male |  |  |  | . | . |
| **AGEGR1** |  | 40-49 |  |  |  |  |  |  | 0.74 | 0.4604 |
| **AGEGR1** |  | 20-29 |  |  |  |  |  |  | 0.45 | 0.6538 |
| **AGEGR1** |  | 30-39 |  |  |  |  |  |  | 0.75 | 0.4534 |
| **AGEGR1** |  | 50-60 |  |  |  |  |  |  | 0.15 | 0.8833 |
| **AGEGR1** |  | < 20 |  |  |  |  |  |  | . | . |
| **RELG** |  |  | Islam |  |  |  |  |  | -0.10 | 0.9171 |
| **RELG** |  |  | Christianity |  |  |  |  |  | . | . |
| **MARSTS** |  |  |  |  |  | Married |  |  | -1.06 | 0.2902 |
| **MARSTS** |  |  |  |  |  | Single |  |  | -0.62 | 0.5340 |
| **MARSTS** |  |  |  |  |  | Widowed |  |  | -0.18 | 0.8549 |
| **MARSTS** |  |  |  |  |  | Divorcedo |  |  | . | . |
| **EDC** |  |  |  |  |  |  | None |  | -0.37 | 0.7082 |
| **EDC** |  |  |  |  |  |  | Secondary |  | -1.29 | 0.1983 |
| **EDC** |  |  |  |  |  |  | Quranic |  | -0.97 | 0.3322 |
| **EDC** |  |  |  |  |  |  | Tertiary |  | 0.57 | 0.5672 |
| **EDC** |  |  |  |  |  |  | Primary |  | . | . |
| **OCC** |  |  |  |  |  |  |  | Unemployed | -0.32 | 0.7477 |
| **OCC** |  |  |  |  |  |  |  | Trader | 0.31 | 0.7560 |
| **OCC** |  |  |  |  |  |  |  | Artisan | 0.15 | 0.8792 |
| **OCC** |  |  |  |  |  |  |  | Entrepreneur | -0.81 | 0.4194 |
| **OCC** |  |  |  |  |  |  |  | Civil servant | . | . |

| **Type 3 Tests of Fixed Effects** | | | | |
| --- | --- | --- | --- | --- |
| **Effect** | **Num DF** | **Den DF** | **F Value** | **Pr > F** |
| **ARM** | 1 | 885 | 4.14 | 0.0422 |
| **VISIT** | 1 | 175 | 18.81 | <.0001 |
| **VISIT*ARM** | 1 | 885 | 0.43 | 0.5145 |
| **RACE** | 3 | 885 | 1.34 | 0.2602 |
| **FAMMEM** | 2 | 885 | 0.34 | 0.7147 |
| **INGR1** | 2 | 885 | 0.32 | 0.7258 |
| **SEX** | 1 | 885 | 0.67 | 0.4133 |
| **AGEGR1** | 4 | 885 | 0.51 | 0.7310 |
| **RELG** | 1 | 885 | 0.01 | 0.9171 |
| **MARSTS** | 3 | 885 | 0.46 | 0.7113 |
| **EDC** | 4 | 885 | 1.42 | 0.2240 |
| **OCC** | 4 | 885 | 0.42 | 0.7926 |

| **Estimates** | | | | | | | | |
| --- | --- | --- | --- | --- | --- | --- | --- | --- |
| **Label** | **Estimate** | **Standard Error** | **DF** | **t Value** | **Pr > \|t\|** | **Alpha** | **Lower** | **Upper** |
| **Treatment effect overtime** | -0.02031 | 0.03114 | 885 | -0.65 | 0.5145 | 0.05 | -0.08144 | 0.04081 |

| **Least Squares Means** | | | | | | |
| --- | --- | --- | --- | --- | --- | --- |
| **Effect** | **Planned Arm** | **Estimate** | **Standard Error** | **DF** | **t Value** | **Pr > \|t\|** |
| **ARM** | MOG | 25.5420 | 0.8903 | 885 | 28.69 | <.0001 |
| **ARM** | COG | 24.1898 | 0.9091 | 885 | 26.61 | <.0001 |

| **Differences of Least Squares Means** | | | | | | | | | |
| --- | --- | --- | --- | --- | --- | --- | --- | --- | --- |
| **Effect** | **Planned Arm** | **Planned Arm** | **Estimate** | **Standard Error** | **DF** | **t Value** | **Pr > \|t\|** | **Adjustment** | **Adj P** |
| **ARM** | MOG | COG | 1.3522 | 0.7069 | 885 | 1.91 | 0.0561 | Bonferroni | 0.0561 |

| **Model Information** | |
| --- | --- |
| **Data Set** | DATAPATH.ADMOLCRT |
| **Dependent Variable** | WGT |
| **Covariance Structure** | Unstructured |
| **Subject Effect** | SUBJID |
| **Estimation Method** | REML |
| **Residual Variance Method** | Profile |
| **Fixed Effects SE Method** | Model-Based |
| **Degrees of Freedom Method** | Containment |

| **Class Level Information** | | |
| --- | --- | --- |
| **Class** | **Levels** | **Values** |
| **SUBJID** | 177 | 90 91 92 93 94 95 96 97 98 99 100 101 102 103 104 105 106 107 108 109 110 111 112 113 114 115 116 117 118 119 120 121 122 123 124 125 126 127 128 129 130 131 132 133 134 135 136 137 138 139 140 141 142 143 144 145 146 147 148 149 150 151 152 153 154 155 156 157 158 159 160 161 162 163 164 165 166 167 168 169 170 171 172 173 174 175 176 177 1 2 3 4 5 6 7 8 9 10 11 12 13 14 15 16 17 18 19 20 21 22 23 24 25 26 27 28 29 30 31 32 33 34 35 36 37 38 39 40 41 42 43 44 45 46 47 48 49 50 51 52 53 54 55 56 57 58 59 60 61 62 63 64 65 66 67 68 69 70 71 72 73 74 75 76 77 78 79 80 81 82 83 84 85 86 87 88 89 |
| **ARM** | 2 | MOG COG |
| **VISITGR** | 7 | 1 2 3 4 5 6 0 |

| **Dimensions** | |
| --- | --- |
| **Covariance Parameters** | 4 |
| **Columns in X** | 6 |
| **Columns in Z per Subject** | 2 |
| **Subjects** | 177 |
| **Max Obs per Subject** | 7 |

| **Number of Observations** | |
| --- | --- |
| **Number of Observations Read** | 1239 |
| **Number of Observations Used** | 1239 |
| **Number of Observations Not Used** | 0 |

| **Iteration History** | | | |
| --- | --- | --- | --- |
| **Iteration** | **Evaluations** | **-2 Res Log Like** | **Criterion** |
| **0** | 1 | 10055.99317190 |  |
| **1** | 1 | 5210.97496494 | 0.00000000 |

| Convergence criteria met. |
| --- |

| **Covariance Parameter Estimates** | | | | | |
| --- | --- | --- | --- | --- | --- |
| **Cov Parm** | **Subject** | **Estimate** | **Standard Error** | **Z Value** | **Pr Z** |
| **UN(1,1)** | SUBJID | 190.56 | 20.4253 | 9.33 | <.0001 |
| **UN(2,1)** | SUBJID | 0.6035 | 0.5446 | 1.11 | 0.2678 |
| **UN(2,2)** | SUBJID | 0.2319 | 0.02896 | 8.01 | <.0001 |
| **Residual** |  | 1.0757 | 0.05114 | 21.04 | <.0001 |

| **Fit Statistics** | |
| --- | --- |
| **-2 Res Log Likelihood** | 5211.0 |
| **AIC (Smaller is Better)** | 5219.0 |
| **AICC (Smaller is Better)** | 5219.0 |
| **BIC (Smaller is Better)** | 5231.7 |

| **Null Model Likelihood Ratio Test** | | |
| --- | --- | --- |
| **DF** | **Chi-Square** | **Pr > ChiSq** |
| 3 | 4845.02 | <.0001 |

| **Solution for Fixed Effects** | | | | | | |
| --- | --- | --- | --- | --- | --- | --- |
| **Effect** | **Planned Arm** | **Estimate** | **Standard Error** | **DF** | **t Value** | **Pr > \|t\|** |
| **Intercept** |  | 61.9229 | 1.4735 | 175 | 42.02 | <.0001 |
| **ARM** | MOG | 1.9026 | 2.0780 | 885 | 0.92 | 0.3601 |
| **ARM** | COG | 0 | . | . | . | . |
| **VISIT** |  | 0.2086 | 0.05543 | 175 | 3.76 | 0.0002 |
| **VISIT*ARM** | MOG | -0.04608 | 0.07816 | 885 | -0.59 | 0.5556 |
| **VISIT*ARM** | COG | 0 | . | . | . | . |

| **Type 3 Tests of Fixed Effects** | | | | |
| --- | --- | --- | --- | --- |
| **Effect** | **Num DF** | **Den DF** | **F Value** | **Pr > F** |
| **ARM** | 1 | 885 | 0.84 | 0.3601 |
| **VISIT** | 1 | 175 | 22.54 | <.0001 |
| **VISIT*ARM** | 1 | 885 | 0.35 | 0.5556 |

| **Estimates** | | | | | | | | |
| --- | --- | --- | --- | --- | --- | --- | --- | --- |
| **Label** | **Estimate** | **Standard Error** | **DF** | **t Value** | **Pr > \|t\|** | **Alpha** | **Lower** | **Upper** |
| **Treatment effect overtime** | -0.04608 | 0.07816 | 885 | -0.59 | 0.5556 | 0.05 | -0.1995 | 0.1073 |

| **Least Squares Means** | | | | | | |
| --- | --- | --- | --- | --- | --- | --- |
| **Effect** | **Planned Arm** | **Estimate** | **Standard Error** | **DF** | **t Value** | **Pr > \|t\|** |
| **ARM** | MOG | 64.3130 | 1.4856 | 885 | 43.29 | <.0001 |
| **ARM** | COG | 62.5487 | 1.4940 | 885 | 41.87 | <.0001 |

| **Differences of Least Squares Means** | | | | | | | |
| --- | --- | --- | --- | --- | --- | --- | --- |
| **Effect** | **Planned Arm** | **Planned Arm** | **Estimate** | **Standard Error** | **DF** | **t Value** | **Pr > \|t\|** |
| **ARM** | MOG | COG | 1.7643 | 2.1069 | 885 | 0.84 | 0.4026 |

| **Model Information** | |
| --- | --- |
| **Data Set** | DATAPATH.ADMOLCRT |
| **Dependent Variable** | WGT |
| **Covariance Structure** | Unstructured |
| **Subject Effect** | SUBJID |
| **Estimation Method** | REML |
| **Residual Variance Method** | Profile |
| **Fixed Effects SE Method** | Model-Based |
| **Degrees of Freedom Method** | Containment |

| **Class Level Information** | | |
| --- | --- | --- |
| **Class** | **Levels** | **Values** |
| **SUBJID** | 177 | 90 91 92 93 94 95 96 97 98 99 100 101 102 103 104 105 106 107 108 109 110 111 112 113 114 115 116 117 118 119 120 121 122 123 124 125 126 127 128 129 130 131 132 133 134 135 136 137 138 139 140 141 142 143 144 145 146 147 148 149 150 151 152 153 154 155 156 157 158 159 160 161 162 163 164 165 166 167 168 169 170 171 172 173 174 175 176 177 1 2 3 4 5 6 7 8 9 10 11 12 13 14 15 16 17 18 19 20 21 22 23 24 25 26 27 28 29 30 31 32 33 34 35 36 37 38 39 40 41 42 43 44 45 46 47 48 49 50 51 52 53 54 55 56 57 58 59 60 61 62 63 64 65 66 67 68 69 70 71 72 73 74 75 76 77 78 79 80 81 82 83 84 85 86 87 88 89 |
| **ARM** | 2 | MOG COG |
| **VISITGR** | 7 | 1 2 3 4 5 6 0 |
| **AGEGR1** | 5 | 40-49 20-29 30-39 50-60 < 20 |
| **RELG** | 2 | Islam Christianity |
| **RACE** | 4 | Hausa / Fulani Yoruba Igbo Other |
| **SEX** | 2 | Female Male |
| **MARSTS** | 4 | Married Single Widowed Divorcedo |
| **EDC** | 5 | None Secondary Quranic Tertiary Primary |
| **OCC** | 5 | Unemployed Trader Artisan Entrepreneur Civil servant |
| **FAMMEM** | 3 | 6-10 10> 2-5 |
| **INGR1** | 3 | Minimum Income and below Above minimum Income Not Indicated |

| **Dimensions** | |
| --- | --- |
| **Covariance Parameters** | 4 |
| **Columns in X** | 39 |
| **Columns in Z per Subject** | 2 |
| **Subjects** | 177 |
| **Max Obs per Subject** | 7 |

| **Number of Observations** | |
| --- | --- |
| **Number of Observations Read** | 1239 |
| **Number of Observations Used** | 1239 |
| **Number of Observations Not Used** | 0 |

| **Iteration History** | | | |
| --- | --- | --- | --- |
| **Iteration** | **Evaluations** | **-2 Res Log Like** | **Criterion** |
| **0** | 1 | 9838.02440893 |  |
| **1** | 2 | 5088.24724876 | 0.00000145 |
| **2** | 1 | 5088.24517474 | 0.00000000 |

| Convergence criteria met. |
| --- |

| **Covariance Parameter Estimates** | | | | | |
| --- | --- | --- | --- | --- | --- |
| **Cov Parm** | **Subject** | **Estimate** | **Standard Error** | **Z Value** | **Pr Z** |
| **UN(1,1)** | SUBJID | 191.25 | 22.0760 | 8.66 | <.0001 |
| **UN(2,1)** | SUBJID | 0.8563 | 0.5984 | 1.43 | 0.1525 |
| **UN(2,2)** | SUBJID | 0.2319 | 0.02896 | 8.01 | <.0001 |
| **Residual** |  | 1.0757 | 0.05114 | 21.04 | <.0001 |

| **Fit Statistics** | |
| --- | --- |
| **-2 Res Log Likelihood** | 5088.2 |
| **AIC (Smaller is Better)** | 5096.2 |
| **AICC (Smaller is Better)** | 5096.3 |
| **BIC (Smaller is Better)** | 5108.9 |

| **Null Model Likelihood Ratio Test** | | |
| --- | --- | --- |
| **DF** | **Chi-Square** | **Pr > ChiSq** |
| 3 | 4749.78 | <.0001 |

| **Solution for Fixed Effects** | | | | | | | | | |
| --- | --- | --- | --- | --- | --- | --- | --- | --- | --- |
| **Effect** | **Planned Arm** | **Age Group** | **Religion** | **Race** | **Sex** | **Marital Status** | **Education** | **Occupation** | **Number of Family Members** |
| **Intercept** |  |  |  |  |  |  |  |  |  |
| **ARM** | MOG |  |  |  |  |  |  |  |  |
| **ARM** | COG |  |  |  |  |  |  |  |  |
| **VISIT** |  |  |  |  |  |  |  |  |  |
| **VISIT*ARM** | MOG |  |  |  |  |  |  |  |  |
| **VISIT*ARM** | COG |  |  |  |  |  |  |  |  |
| **RACE** |  |  |  | Hausa / Fulani |  |  |  |  |  |
| **RACE** |  |  |  | Yoruba |  |  |  |  |  |
| **RACE** |  |  |  | Igbo |  |  |  |  |  |
| **RACE** |  |  |  | Other |  |  |  |  |  |
| **FAMMEM** |  |  |  |  |  |  |  |  | 6-10 |
| **FAMMEM** |  |  |  |  |  |  |  |  | 10> |
| **FAMMEM** |  |  |  |  |  |  |  |  | 2-5 |
| **INGR1** |  |  |  |  |  |  |  |  |  |
| **INGR1** |  |  |  |  |  |  |  |  |  |
| **INGR1** |  |  |  |  |  |  |  |  |  |
| **SEX** |  |  |  |  | Female |  |  |  |  |
| **SEX** |  |  |  |  | Male |  |  |  |  |
| **AGEGR1** |  | 40-49 |  |  |  |  |  |  |  |
| **AGEGR1** |  | 20-29 |  |  |  |  |  |  |  |
| **AGEGR1** |  | 30-39 |  |  |  |  |  |  |  |
| **AGEGR1** |  | 50-60 |  |  |  |  |  |  |  |
| **AGEGR1** |  | < 20 |  |  |  |  |  |  |  |
| **RELG** |  |  | Islam |  |  |  |  |  |  |
| **RELG** |  |  | Christianity |  |  |  |  |  |  |
| **MARSTS** |  |  |  |  |  | Married |  |  |  |
| **MARSTS** |  |  |  |  |  | Single |  |  |  |
| **MARSTS** |  |  |  |  |  | Widowed |  |  |  |
| **MARSTS** |  |  |  |  |  | Divorcedo |  |  |  |
| **EDC** |  |  |  |  |  |  | None |  |  |
| **EDC** |  |  |  |  |  |  | Secondary |  |  |
| **EDC** |  |  |  |  |  |  | Quranic |  |  |
| **EDC** |  |  |  |  |  |  | Tertiary |  |  |
| **EDC** |  |  |  |  |  |  | Primary |  |  |
| **OCC** |  |  |  |  |  |  |  | Unemployed |  |
| **OCC** |  |  |  |  |  |  |  | Trader |  |
| **OCC** |  |  |  |  |  |  |  | Artisan |  |
| **OCC** |  |  |  |  |  |  |  | Entrepreneur |  |
| **OCC** |  |  |  |  |  |  |  | Civil servant |  |

| **Solution for Fixed Effects** | | | | | | | | | |
| --- | --- | --- | --- | --- | --- | --- | --- | --- | --- |
| **Effect** | **Planned Arm** | **Age Group** | **Religion** | **Race** | **Sex** | **Marital Status** | **Education** | **Occupation** | **Annual Income Group** |
| **Intercept** |  |  |  |  |  |  |  |  |  |
| **ARM** | MOG |  |  |  |  |  |  |  |  |
| **ARM** | COG |  |  |  |  |  |  |  |  |
| **VISIT** |  |  |  |  |  |  |  |  |  |
| **VISIT*ARM** | MOG |  |  |  |  |  |  |  |  |
| **VISIT*ARM** | COG |  |  |  |  |  |  |  |  |
| **RACE** |  |  |  | Hausa / Fulani |  |  |  |  |  |
| **RACE** |  |  |  | Yoruba |  |  |  |  |  |
| **RACE** |  |  |  | Igbo |  |  |  |  |  |
| **RACE** |  |  |  | Other |  |  |  |  |  |
| **FAMMEM** |  |  |  |  |  |  |  |  |  |
| **FAMMEM** |  |  |  |  |  |  |  |  |  |
| **FAMMEM** |  |  |  |  |  |  |  |  |  |
| **INGR1** |  |  |  |  |  |  |  |  | Minimum Income and below |
| **INGR1** |  |  |  |  |  |  |  |  | Above minimum Income |
| **INGR1** |  |  |  |  |  |  |  |  | Not Indicated |
| **SEX** |  |  |  |  | Female |  |  |  |  |
| **SEX** |  |  |  |  | Male |  |  |  |  |
| **AGEGR1** |  | 40-49 |  |  |  |  |  |  |  |
| **AGEGR1** |  | 20-29 |  |  |  |  |  |  |  |
| **AGEGR1** |  | 30-39 |  |  |  |  |  |  |  |
| **AGEGR1** |  | 50-60 |  |  |  |  |  |  |  |
| **AGEGR1** |  | < 20 |  |  |  |  |  |  |  |
| **RELG** |  |  | Islam |  |  |  |  |  |  |
| **RELG** |  |  | Christianity |  |  |  |  |  |  |
| **MARSTS** |  |  |  |  |  | Married |  |  |  |
| **MARSTS** |  |  |  |  |  | Single |  |  |  |
| **MARSTS** |  |  |  |  |  | Widowed |  |  |  |
| **MARSTS** |  |  |  |  |  | Divorcedo |  |  |  |
| **EDC** |  |  |  |  |  |  | None |  |  |
| **EDC** |  |  |  |  |  |  | Secondary |  |  |
| **EDC** |  |  |  |  |  |  | Quranic |  |  |
| **EDC** |  |  |  |  |  |  | Tertiary |  |  |
| **EDC** |  |  |  |  |  |  | Primary |  |  |
| **OCC** |  |  |  |  |  |  |  | Unemployed |  |
| **OCC** |  |  |  |  |  |  |  | Trader |  |
| **OCC** |  |  |  |  |  |  |  | Artisan |  |
| **OCC** |  |  |  |  |  |  |  | Entrepreneur |  |
| **OCC** |  |  |  |  |  |  |  | Civil servant |  |

| **Solution for Fixed Effects** | | | | | | | | | |
| --- | --- | --- | --- | --- | --- | --- | --- | --- | --- |
| **Effect** | **Planned Arm** | **Age Group** | **Religion** | **Race** | **Sex** | **Marital Status** | **Education** | **Occupation** | **Estimate** |
| **Intercept** |  |  |  |  |  |  |  |  | 57.6995 |
| **ARM** | MOG |  |  |  |  |  |  |  | 3.1373 |
| **ARM** | COG |  |  |  |  |  |  |  | 0 |
| **VISIT** |  |  |  |  |  |  |  |  | 0.2086 |
| **VISIT*ARM** | MOG |  |  |  |  |  |  |  | -0.04608 |
| **VISIT*ARM** | COG |  |  |  |  |  |  |  | 0 |
| **RACE** |  |  |  | Hausa / Fulani |  |  |  |  | -2.7028 |
| **RACE** |  |  |  | Yoruba |  |  |  |  | -2.3950 |
| **RACE** |  |  |  | Igbo |  |  |  |  | 3.0270 |
| **RACE** |  |  |  | Other |  |  |  |  | 0 |
| **FAMMEM** |  |  |  |  |  |  |  |  | -0.1739 |
| **FAMMEM** |  |  |  |  |  |  |  |  | -0.9283 |
| **FAMMEM** |  |  |  |  |  |  |  |  | 0 |
| **INGR1** |  |  |  |  |  |  |  |  | 1.4736 |
| **INGR1** |  |  |  |  |  |  |  |  | 5.4595 |
| **INGR1** |  |  |  |  |  |  |  |  | 0 |
| **SEX** |  |  |  |  | Female |  |  |  | -2.7034 |
| **SEX** |  |  |  |  | Male |  |  |  | 0 |
| **AGEGR1** |  | 40-49 |  |  |  |  |  |  | 7.9597 |
| **AGEGR1** |  | 20-29 |  |  |  |  |  |  | 3.8570 |
| **AGEGR1** |  | 30-39 |  |  |  |  |  |  | 6.0788 |
| **AGEGR1** |  | 50-60 |  |  |  |  |  |  | 3.0057 |
| **AGEGR1** |  | < 20 |  |  |  |  |  |  | 0 |
| **RELG** |  |  | Islam |  |  |  |  |  | 0.7165 |
| **RELG** |  |  | Christianity |  |  |  |  |  | 0 |
| **MARSTS** |  |  |  |  |  | Married |  |  | -1.4055 |
| **MARSTS** |  |  |  |  |  | Single |  |  | -2.0011 |
| **MARSTS** |  |  |  |  |  | Widowed |  |  | 1.9724 |
| **MARSTS** |  |  |  |  |  | Divorcedo |  |  | 0 |
| **EDC** |  |  |  |  |  |  | None |  | 0.8899 |
| **EDC** |  |  |  |  |  |  | Secondary |  | -4.1279 |
| **EDC** |  |  |  |  |  |  | Quranic |  | -5.1077 |
| **EDC** |  |  |  |  |  |  | Tertiary |  | 2.6682 |
| **EDC** |  |  |  |  |  |  | Primary |  | 0 |
| **OCC** |  |  |  |  |  |  |  | Unemployed | 0.4189 |
| **OCC** |  |  |  |  |  |  |  | Trader | 2.2527 |
| **OCC** |  |  |  |  |  |  |  | Artisan | 4.4105 |
| **OCC** |  |  |  |  |  |  |  | Entrepreneur | -2.7305 |
| **OCC** |  |  |  |  |  |  |  | Civil servant | 0 |

| **Solution for Fixed Effects** | | | | | | | | | | |
| --- | --- | --- | --- | --- | --- | --- | --- | --- | --- | --- |
| **Effect** | **Planned Arm** | **Age Group** | **Religion** | **Race** | **Sex** | **Marital Status** | **Education** | **Occupation** | **Standard Error** | **DF** |
| **Intercept** |  |  |  |  |  |  |  |  | 10.8186 | 151 |
| **ARM** | MOG |  |  |  |  |  |  |  | 2.1676 | 885 |
| **ARM** | COG |  |  |  |  |  |  |  | . | . |
| **VISIT** |  |  |  |  |  |  |  |  | 0.05543 | 175 |
| **VISIT*ARM** | MOG |  |  |  |  |  |  |  | 0.07816 | 885 |
| **VISIT*ARM** | COG |  |  |  |  |  |  |  | . | . |
| **RACE** |  |  |  | Hausa / Fulani |  |  |  |  | 3.4706 | 885 |
| **RACE** |  |  |  | Yoruba |  |  |  |  | 4.1139 | 885 |
| **RACE** |  |  |  | Igbo |  |  |  |  | 4.3611 | 885 |
| **RACE** |  |  |  | Other |  |  |  |  | . | . |
| **FAMMEM** |  |  |  |  |  |  |  |  | 2.7541 | 885 |
| **FAMMEM** |  |  |  |  |  |  |  |  | 2.6965 | 885 |
| **FAMMEM** |  |  |  |  |  |  |  |  | . | . |
| **INGR1** |  |  |  |  |  |  |  |  | 3.9931 | 885 |
| **INGR1** |  |  |  |  |  |  |  |  | 4.8694 | 885 |
| **INGR1** |  |  |  |  |  |  |  |  | . | . |
| **SEX** |  |  |  |  | Female |  |  |  | 2.9891 | 885 |
| **SEX** |  |  |  |  | Male |  |  |  | . | . |
| **AGEGR1** |  | 40-49 |  |  |  |  |  |  | 7.8795 | 885 |
| **AGEGR1** |  | 20-29 |  |  |  |  |  |  | 7.6862 | 885 |
| **AGEGR1** |  | 30-39 |  |  |  |  |  |  | 7.5357 | 885 |
| **AGEGR1** |  | 50-60 |  |  |  |  |  |  | 8.5283 | 885 |
| **AGEGR1** |  | < 20 |  |  |  |  |  |  | . | . |
| **RELG** |  |  | Islam |  |  |  |  |  | 2.7784 | 885 |
| **RELG** |  |  | Christianity |  |  |  |  |  | . | . |
| **MARSTS** |  |  |  |  |  | Married |  |  | 2.9536 | 885 |
| **MARSTS** |  |  |  |  |  | Single |  |  | 4.1653 | 885 |
| **MARSTS** |  |  |  |  |  | Widowed |  |  | 3.3861 | 885 |
| **MARSTS** |  |  |  |  |  | Divorcedo |  |  | . | . |
| **EDC** |  |  |  |  |  |  | None |  | 4.1453 | 885 |
| **EDC** |  |  |  |  |  |  | Secondary |  | 3.6407 | 885 |
| **EDC** |  |  |  |  |  |  | Quranic |  | 3.9194 | 885 |
| **EDC** |  |  |  |  |  |  | Tertiary |  | 4.0260 | 885 |
| **EDC** |  |  |  |  |  |  | Primary |  | . | . |
| **OCC** |  |  |  |  |  |  |  | Unemployed | 3.7803 | 885 |
| **OCC** |  |  |  |  |  |  |  | Trader | 3.5215 | 885 |
| **OCC** |  |  |  |  |  |  |  | Artisan | 3.7410 | 885 |
| **OCC** |  |  |  |  |  |  |  | Entrepreneur | 4.0855 | 885 |
| **OCC** |  |  |  |  |  |  |  | Civil servant | . | . |

| **Solution for Fixed Effects** | | | | | | | | | | |
| --- | --- | --- | --- | --- | --- | --- | --- | --- | --- | --- |
| **Effect** | **Planned Arm** | **Age Group** | **Religion** | **Race** | **Sex** | **Marital Status** | **Education** | **Occupation** | **t Value** | **Pr > \|t\|** |
| **Intercept** |  |  |  |  |  |  |  |  | 5.33 | <.0001 |
| **ARM** | MOG |  |  |  |  |  |  |  | 1.45 | 0.1482 |
| **ARM** | COG |  |  |  |  |  |  |  | . | . |
| **VISIT** |  |  |  |  |  |  |  |  | 3.76 | 0.0002 |
| **VISIT*ARM** | MOG |  |  |  |  |  |  |  | -0.59 | 0.5556 |
| **VISIT*ARM** | COG |  |  |  |  |  |  |  | . | . |
| **RACE** |  |  |  | Hausa / Fulani |  |  |  |  | -0.78 | 0.4363 |
| **RACE** |  |  |  | Yoruba |  |  |  |  | -0.58 | 0.5606 |
| **RACE** |  |  |  | Igbo |  |  |  |  | 0.69 | 0.4878 |
| **RACE** |  |  |  | Other |  |  |  |  | . | . |
| **FAMMEM** |  |  |  |  |  |  |  |  | -0.06 | 0.9497 |
| **FAMMEM** |  |  |  |  |  |  |  |  | -0.34 | 0.7307 |
| **FAMMEM** |  |  |  |  |  |  |  |  | . | . |
| **INGR1** |  |  |  |  |  |  |  |  | 0.37 | 0.7122 |
| **INGR1** |  |  |  |  |  |  |  |  | 1.12 | 0.2625 |
| **INGR1** |  |  |  |  |  |  |  |  | . | . |
| **SEX** |  |  |  |  | Female |  |  |  | -0.90 | 0.3660 |
| **SEX** |  |  |  |  | Male |  |  |  | . | . |
| **AGEGR1** |  | 40-49 |  |  |  |  |  |  | 1.01 | 0.3127 |
| **AGEGR1** |  | 20-29 |  |  |  |  |  |  | 0.50 | 0.6159 |
| **AGEGR1** |  | 30-39 |  |  |  |  |  |  | 0.81 | 0.4201 |
| **AGEGR1** |  | 50-60 |  |  |  |  |  |  | 0.35 | 0.7246 |
| **AGEGR1** |  | < 20 |  |  |  |  |  |  | . | . |
| **RELG** |  |  | Islam |  |  |  |  |  | 0.26 | 0.7965 |
| **RELG** |  |  | Christianity |  |  |  |  |  | . | . |
| **MARSTS** |  |  |  |  |  | Married |  |  | -0.48 | 0.6343 |
| **MARSTS** |  |  |  |  |  | Single |  |  | -0.48 | 0.6310 |
| **MARSTS** |  |  |  |  |  | Widowed |  |  | 0.58 | 0.5604 |
| **MARSTS** |  |  |  |  |  | Divorcedo |  |  | . | . |
| **EDC** |  |  |  |  |  |  | None |  | 0.21 | 0.8301 |
| **EDC** |  |  |  |  |  |  | Secondary |  | -1.13 | 0.2572 |
| **EDC** |  |  |  |  |  |  | Quranic |  | -1.30 | 0.1929 |
| **EDC** |  |  |  |  |  |  | Tertiary |  | 0.66 | 0.5077 |
| **EDC** |  |  |  |  |  |  | Primary |  | . | . |
| **OCC** |  |  |  |  |  |  |  | Unemployed | 0.11 | 0.9118 |
| **OCC** |  |  |  |  |  |  |  | Trader | 0.64 | 0.5225 |
| **OCC** |  |  |  |  |  |  |  | Artisan | 1.18 | 0.2387 |
| **OCC** |  |  |  |  |  |  |  | Entrepreneur | -0.67 | 0.5041 |
| **OCC** |  |  |  |  |  |  |  | Civil servant | . | . |

| **Type 3 Tests of Fixed Effects** | | | | |
| --- | --- | --- | --- | --- |
| **Effect** | **Num DF** | **Den DF** | **F Value** | **Pr > F** |
| **ARM** | 1 | 885 | 2.09 | 0.1482 |
| **VISIT** | 1 | 175 | 22.54 | <.0001 |
| **VISIT*ARM** | 1 | 885 | 0.35 | 0.5556 |
| **RACE** | 3 | 885 | 0.96 | 0.4113 |
| **FAMMEM** | 2 | 885 | 0.06 | 0.9385 |
| **INGR1** | 2 | 885 | 0.86 | 0.4244 |
| **SEX** | 1 | 885 | 0.82 | 0.3660 |
| **AGEGR1** | 4 | 885 | 0.69 | 0.6000 |
| **RELG** | 1 | 885 | 0.07 | 0.7965 |
| **MARSTS** | 3 | 885 | 0.47 | 0.7034 |
| **EDC** | 4 | 885 | 1.81 | 0.1244 |
| **OCC** | 4 | 885 | 1.00 | 0.4044 |

| **Estimates** | | | | | | | | |
| --- | --- | --- | --- | --- | --- | --- | --- | --- |
| **Label** | **Estimate** | **Standard Error** | **DF** | **t Value** | **Pr > \|t\|** | **Alpha** | **Lower** | **Upper** |
| **Treatment effect overtime** | -0.04608 | 0.07816 | 885 | -0.59 | 0.5556 | 0.05 | -0.1995 | 0.1073 |

| **Least Squares Means** | | | | | | |
| --- | --- | --- | --- | --- | --- | --- |
| **Effect** | **Planned Arm** | **Estimate** | **Standard Error** | **DF** | **t Value** | **Pr > \|t\|** |
| **ARM** | MOG | 65.3134 | 2.7720 | 885 | 23.56 | <.0001 |
| **ARM** | COG | 62.3144 | 2.8304 | 885 | 22.02 | <.0001 |

| **Differences of Least Squares Means** | | | | | | | | | |
| --- | --- | --- | --- | --- | --- | --- | --- | --- | --- |
| **Effect** | **Planned Arm** | **Planned Arm** | **Estimate** | **Standard Error** | **DF** | **t Value** | **Pr > \|t\|** | **Adjustment** | **Adj P** |
| **ARM** | MOG | COG | 2.9990 | 2.2032 | 885 | 1.36 | 0.1738 | Bonferroni | 0.1738 |

| **Model Information** | |
| --- | --- |
| **Data Set** | DATAPATH.ADMOLCRT |
| **Response Variable** | VL |
| **Response Distribution** | Binary |
| **Link Function** | Logit |
| **Variance Function** | Default |
| **Variance Matrix Blocked By** | SUBJID |
| **Estimation Technique** | Residual PL |
| **Degrees of Freedom Method** | Containment |

| **Class Level Information** | | |
| --- | --- | --- |
| **Class** | **Levels** | **Values** |
| **SUBJID** | 177 | 90 91 92 93 94 95 96 97 98 99 100 101 102 103 104 105 106 107 108 109 110 111 112 113 114 115 116 117 118 119 120 121 122 123 124 125 126 127 128 129 130 131 132 133 134 135 136 137 138 139 140 141 142 143 144 145 146 147 148 149 150 151 152 153 154 155 156 157 158 159 160 161 162 163 164 165 166 167 168 169 170 171 172 173 174 175 176 177 1 2 3 4 5 6 7 8 9 10 11 12 13 14 15 16 17 18 19 20 21 22 23 24 25 26 27 28 29 30 31 32 33 34 35 36 37 38 39 40 41 42 43 44 45 46 47 48 49 50 51 52 53 54 55 56 57 58 59 60 61 62 63 64 65 66 67 68 69 70 71 72 73 74 75 76 77 78 79 80 81 82 83 84 85 86 87 88 89 |
| **ARM** | 2 | MOG COG |
| **VISITGR** | 2 | 6 0 |

| **Number of Observations Read** | 354 |
| --- | --- |
| **Number of Observations Used** | 354 |

| **Response Profile** | | |
| --- | --- | --- |
| **Ordered Value** | **VL** | **Total Frequency** |
| **1** | 0 | 270 |
| **2** | 1 | 84 |
| **The GLIMMIX procedure is modeling the probability that VL='1'.** | | |

| **Dimensions** | |
| --- | --- |
| **G-side Cov. Parameters** | 1 |
| **Columns in X** | 6 |
| **Columns in Z per Subject** | 1 |
| **Subjects (Blocks in V)** | 177 |
| **Max Obs per Subject** | 2 |

| **Optimization Information** | |
| --- | --- |
| **Optimization Technique** | Newton-Raphson with Ridging |
| **Parameters in Optimization** | 1 |
| **Lower Boundaries** | 1 |
| **Upper Boundaries** | 0 |
| **Fixed Effects** | Profiled |
| **Starting From** | Data |

| **Iteration History** | | | | | |
| --- | --- | --- | --- | --- | --- |
| **Iteration** | **Restarts** | **Subiterations** | **Objective Function** | **Change** | **Max Gradient** |
| **0** | **0** | **4** | 1591.7898271 | 2.00000000 | 1.525E-6 |
| **1** | **0** | **3** | 1620.8790433 | 0.13309221 | 2.548E-7 |
| **2** | **0** | **3** | 1635.7884715 | 0.05400083 | 2.99E-10 |
| **3** | **0** | **2** | 1641.113724 | 0.01865150 | 1.131E-6 |
| **4** | **0** | **2** | 1642.9330411 | 0.00626119 | 1.484E-8 |
| **5** | **0** | **2** | 1643.541219 | 0.00208032 | 1.83E-10 |
| **6** | **0** | **1** | 1643.7429945 | 0.00068837 | 6.147E-6 |
| **7** | **0** | **1** | 1643.8097283 | 0.00022802 | 6.743E-7 |
| **8** | **0** | **1** | 1643.8318295 | 0.00007542 | 7.376E-8 |
| **9** | **0** | **1** | 1643.8391392 | 0.00002493 | 8.061E-9 |
| **10** | **0** | **1** | 1643.8415557 | 0.00000824 | 8.81E-10 |
| **11** | **0** | **1** | 1643.8423544 | 0.00000272 | 9.62E-11 |
| **12** | **0** | **1** | 1643.8426184 | 0.00000090 | 1.04E-11 |
| **13** | **0** | **0** | 1643.8427057 | 0.00000000 | 4.434E-6 |

| Convergence criterion (PCONV=1.11022E-8) satisfied. |
| --- |

| **Fit Statistics** | |
| --- | --- |
| **-2 Res Log Pseudo-Likelihood** | 1643.84 |
| **Generalized Chi-Square** | 197.05 |
| **Gener. Chi-Square / DF** | 0.56 |

| **Covariance Parameter Estimates** | | | |
| --- | --- | --- | --- |
| **Cov Parm** | **Subject** | **Estimate** | **Standard Error** |
| **Intercept** | SUBJID | 2.1790 | 0.5408 |

| **Solutions for Fixed Effects** | | | | | | |
| --- | --- | --- | --- | --- | --- | --- |
| **Effect** | **Planned Arm** | **Estimate** | **Standard Error** | **DF** | **t Value** | **Pr > \|t\|** |
| **Intercept** |  | -1.0709 | 0.3085 | 175 | -3.47 | 0.0007 |
| **ARM** | MOG | -0.2721 | 0.4434 | 175 | -0.61 | 0.5402 |
| **ARM** | COG | 0 | . | . | . | . |
| **VISIT** |  | -0.04857 | 0.06377 | 175 | -0.76 | 0.4473 |
| **VISIT*ARM** | MOG | -0.00514 | 0.09258 | 175 | -0.06 | 0.9558 |
| **VISIT*ARM** | COG | 0 | . | . | . | . |

| **Type III Tests of Fixed Effects** | | | | |
| --- | --- | --- | --- | --- |
| **Effect** | **Num DF** | **Den DF** | **F Value** | **Pr > F** |
| **ARM** | 1 | 175 | 0.38 | 0.5402 |
| **VISIT** | 1 | 175 | 1.22 | 0.2708 |
| **VISIT*ARM** | 1 | 175 | 0.00 | 0.9558 |

| **Estimates** | | | | | | | | | | | | |
| --- | --- | --- | --- | --- | --- | --- | --- | --- | --- | --- | --- | --- |
| **Label** | **Estimate** | **Standard Error** | **DF** | **t Value** | **Pr > \|t\|** | **Alpha** | **Lower** | **Upper** | **Mean** | **Standard Error Mean** | **Lower Mean** | **Upper Mean** |
| **Treatment effect overtime** | -0.00514 | 0.09258 | 175 | -0.06 | 0.9558 | 0.05 | -0.1879 | 0.1776 | 0.4987 | 0.02315 | 0.4532 | 0.5443 |

| **ARM Least Squares Means** | | | | | | | | | | | | |
| --- | --- | --- | --- | --- | --- | --- | --- | --- | --- | --- | --- | --- |
| **Planned Arm** | **Estimate** | **Standard Error** | **DF** | **t Value** | **Pr > \|t\|** | **Alpha** | **Lower** | **Upper** | **Mean** | **Standard Error Mean** | **Lower Mean** | **Upper Mean** |
| MOG | -1.5041 | 0.2595 | 175 | -5.80 | <.0001 | 0.05 | -2.0163 | -0.9919 | 0.1818 | 0.03861 | 0.1175 | 0.2705 |
| COG | -1.2166 | 0.2508 | 175 | -4.85 | <.0001 | 0.05 | -1.7116 | -0.7215 | 0.2285 | 0.04423 | 0.1530 | 0.3271 |

| **Differences of ARM Least Squares Means** | | | | | | | | | |
| --- | --- | --- | --- | --- | --- | --- | --- | --- | --- |
| **Planned Arm** | **Planned Arm** | **Estimate** | **Standard Error** | **DF** | **t Value** | **Pr > \|t\|** | **Alpha** | **Lower** | **Upper** |
| MOG | COG | -0.2875 | 0.3609 | 175 | -0.80 | 0.4267 | 0.05 | -0.9999 | 0.4248 |

| **Model Information** | |
| --- | --- |
| **Data Set** | DATAPATH.ADMOLCRT |
| **Response Variable** | VL |
| **Response Distribution** | Binary |
| **Link Function** | Logit |
| **Variance Function** | Default |
| **Variance Matrix Blocked By** | SUBJID |
| **Estimation Technique** | Residual PL |
| **Degrees of Freedom Method** | Containment |

| **Class Level Information** | | |
| --- | --- | --- |
| **Class** | **Levels** | **Values** |
| **SUBJID** | 177 | 90 91 92 93 94 95 96 97 98 99 100 101 102 103 104 105 106 107 108 109 110 111 112 113 114 115 116 117 118 119 120 121 122 123 124 125 126 127 128 129 130 131 132 133 134 135 136 137 138 139 140 141 142 143 144 145 146 147 148 149 150 151 152 153 154 155 156 157 158 159 160 161 162 163 164 165 166 167 168 169 170 171 172 173 174 175 176 177 1 2 3 4 5 6 7 8 9 10 11 12 13 14 15 16 17 18 19 20 21 22 23 24 25 26 27 28 29 30 31 32 33 34 35 36 37 38 39 40 41 42 43 44 45 46 47 48 49 50 51 52 53 54 55 56 57 58 59 60 61 62 63 64 65 66 67 68 69 70 71 72 73 74 75 76 77 78 79 80 81 82 83 84 85 86 87 88 89 |
| **ARM** | 2 | MOG COG |
| **VISITGR** | 2 | 6 0 |
| **AGEGR1** | 5 | 40-49 20-29 30-39 50-60 < 20 |
| **RELG** | 2 | Islam Christianity |
| **RACE** | 4 | Hausa / Fulani Yoruba Igbo Other |
| **SEX** | 2 | Female Male |
| **MARSTS** | 4 | Married Single Widowed Divorcedo |
| **EDC** | 5 | None Secondary Quranic Tertiary Primary |
| **OCC** | 5 | Unemployed Trader Artisan Entrepreneur Civil servant |
| **FAMMEM** | 3 | 6-10 10> 2-5 |
| **INGR1** | 3 | Minimum Income and below Above minimum Income Not Indicated |

| **Number of Observations Read** | 354 |
| --- | --- |
| **Number of Observations Used** | 354 |

| **Response Profile** | | |
| --- | --- | --- |
| **Ordered Value** | **VL** | **Total Frequency** |
| **1** | 0 | 270 |
| **2** | 1 | 84 |
| **The GLIMMIX procedure is modeling the probability that VL='1'.** | | |

| **Dimensions** | |
| --- | --- |
| **G-side Cov. Parameters** | 1 |
| **Columns in X** | 39 |
| **Columns in Z per Subject** | 1 |
| **Subjects (Blocks in V)** | 177 |
| **Max Obs per Subject** | 2 |

| **Optimization Information** | |
| --- | --- |
| **Optimization Technique** | Newton-Raphson with Ridging |
| **Parameters in Optimization** | 1 |
| **Lower Boundaries** | 1 |
| **Upper Boundaries** | 0 |
| **Fixed Effects** | Profiled |
| **Starting From** | Data |

| **Iteration History** | | | | | |
| --- | --- | --- | --- | --- | --- |
| **Iteration** | **Restarts** | **Subiterations** | **Objective Function** | **Change** | **Max Gradient** |
| **0** | **0** | **4** | 1585.2649107 | 2.00000000 | 4.041E-6 |
| **1** | **0** | **4** | 1648.7110505 | 2.00000000 | 2.58E-10 |
| **2** | **0** | **3** | 1685.3608159 | 1.06766175 | 2.961E-7 |
| **3** | **0** | **3** | 1700.7431772 | 0.12003489 | 2.66E-10 |
| **4** | **0** | **2** | 1706.5147129 | 0.03934123 | 1.029E-6 |
| **5** | **0** | **2** | 1708.574251 | 0.01340027 | 1.625E-8 |
| **6** | **0** | **2** | 1709.2937574 | 0.00461241 | 2.4E-10 |
| **7** | **0** | **1** | 1709.5431169 | 0.00158958 | 6.082E-6 |
| **8** | **0** | **1** | 1709.6292303 | 0.00054951 | 7.279E-7 |
| **9** | **0** | **1** | 1709.659019 | 0.00018974 | 8.684E-8 |
| **10** | **0** | **1** | 1709.6693072 | 0.00006549 | 1.035E-8 |
| **11** | **0** | **1** | 1709.6728586 | 0.00002260 | 1.232E-9 |
| **12** | **0** | **1** | 1709.6740842 | 0.00000780 | 1.47E-10 |
| **13** | **0** | **1** | 1709.6745072 | 0.00000269 | 1.75E-11 |
| **14** | **0** | **0** | 1709.6746531 | 0.00000000 | 4.728E-6 |

| Convergence criterion (PCONV=1.11022E-8) satisfied. |
| --- |

| **Fit Statistics** | |
| --- | --- |
| **-2 Res Log Pseudo-Likelihood** | 1709.67 |
| **Generalized Chi-Square** | 166.04 |
| **Gener. Chi-Square / DF** | 0.51 |

| **Covariance Parameter Estimates** | | | |
| --- | --- | --- | --- |
| **Cov Parm** | **Subject** | **Estimate** | **Standard Error** |
| **Intercept** | SUBJID | 3.3247 | 0.7869 |

| **Solutions for Fixed Effects** | | | | | | | | | |
| --- | --- | --- | --- | --- | --- | --- | --- | --- | --- |
| **Effect** | **Planned Arm** | **Age Group** | **Religion** | **Race** | **Sex** | **Marital Status** | **Education** | **Occupation** | **Number of Family Members** |
| **Intercept** |  |  |  |  |  |  |  |  |  |
| **ARM** | MOG |  |  |  |  |  |  |  |  |
| **ARM** | COG |  |  |  |  |  |  |  |  |
| **VISIT** |  |  |  |  |  |  |  |  |  |
| **VISIT*ARM** | MOG |  |  |  |  |  |  |  |  |
| **VISIT*ARM** | COG |  |  |  |  |  |  |  |  |
| **RACE** |  |  |  | Hausa / Fulani |  |  |  |  |  |
| **RACE** |  |  |  | Yoruba |  |  |  |  |  |
| **RACE** |  |  |  | Igbo |  |  |  |  |  |
| **RACE** |  |  |  | Other |  |  |  |  |  |
| **FAMMEM** |  |  |  |  |  |  |  |  | 6-10 |
| **FAMMEM** |  |  |  |  |  |  |  |  | 10> |
| **FAMMEM** |  |  |  |  |  |  |  |  | 2-5 |
| **INGR1** |  |  |  |  |  |  |  |  |  |
| **INGR1** |  |  |  |  |  |  |  |  |  |
| **INGR1** |  |  |  |  |  |  |  |  |  |
| **SEX** |  |  |  |  | Female |  |  |  |  |
| **SEX** |  |  |  |  | Male |  |  |  |  |
| **AGEGR1** |  | 40-49 |  |  |  |  |  |  |  |
| **AGEGR1** |  | 20-29 |  |  |  |  |  |  |  |
| **AGEGR1** |  | 30-39 |  |  |  |  |  |  |  |
| **AGEGR1** |  | 50-60 |  |  |  |  |  |  |  |
| **AGEGR1** |  | < 20 |  |  |  |  |  |  |  |
| **RELG** |  |  | Islam |  |  |  |  |  |  |
| **RELG** |  |  | Christianity |  |  |  |  |  |  |
| **MARSTS** |  |  |  |  |  | Married |  |  |  |
| **MARSTS** |  |  |  |  |  | Single |  |  |  |
| **MARSTS** |  |  |  |  |  | Widowed |  |  |  |
| **MARSTS** |  |  |  |  |  | Divorcedo |  |  |  |
| **EDC** |  |  |  |  |  |  | None |  |  |
| **EDC** |  |  |  |  |  |  | Secondary |  |  |
| **EDC** |  |  |  |  |  |  | Quranic |  |  |
| **EDC** |  |  |  |  |  |  | Tertiary |  |  |
| **EDC** |  |  |  |  |  |  | Primary |  |  |
| **OCC** |  |  |  |  |  |  |  | Unemployed |  |
| **OCC** |  |  |  |  |  |  |  | Trader |  |
| **OCC** |  |  |  |  |  |  |  | Artisan |  |
| **OCC** |  |  |  |  |  |  |  | Entrepreneur |  |
| **OCC** |  |  |  |  |  |  |  | Civil servant |  |

| **Solutions for Fixed Effects** | | | | | | | | | |
| --- | --- | --- | --- | --- | --- | --- | --- | --- | --- |
| **Effect** | **Planned Arm** | **Age Group** | **Religion** | **Race** | **Sex** | **Marital Status** | **Education** | **Occupation** | **Annual Income Group** |
| **Intercept** |  |  |  |  |  |  |  |  |  |
| **ARM** | MOG |  |  |  |  |  |  |  |  |
| **ARM** | COG |  |  |  |  |  |  |  |  |
| **VISIT** |  |  |  |  |  |  |  |  |  |
| **VISIT*ARM** | MOG |  |  |  |  |  |  |  |  |
| **VISIT*ARM** | COG |  |  |  |  |  |  |  |  |
| **RACE** |  |  |  | Hausa / Fulani |  |  |  |  |  |
| **RACE** |  |  |  | Yoruba |  |  |  |  |  |
| **RACE** |  |  |  | Igbo |  |  |  |  |  |
| **RACE** |  |  |  | Other |  |  |  |  |  |
| **FAMMEM** |  |  |  |  |  |  |  |  |  |
| **FAMMEM** |  |  |  |  |  |  |  |  |  |
| **FAMMEM** |  |  |  |  |  |  |  |  |  |
| **INGR1** |  |  |  |  |  |  |  |  | Minimum Income and below |
| **INGR1** |  |  |  |  |  |  |  |  | Above minimum Income |
| **INGR1** |  |  |  |  |  |  |  |  | Not Indicated |
| **SEX** |  |  |  |  | Female |  |  |  |  |
| **SEX** |  |  |  |  | Male |  |  |  |  |
| **AGEGR1** |  | 40-49 |  |  |  |  |  |  |  |
| **AGEGR1** |  | 20-29 |  |  |  |  |  |  |  |
| **AGEGR1** |  | 30-39 |  |  |  |  |  |  |  |
| **AGEGR1** |  | 50-60 |  |  |  |  |  |  |  |
| **AGEGR1** |  | < 20 |  |  |  |  |  |  |  |
| **RELG** |  |  | Islam |  |  |  |  |  |  |
| **RELG** |  |  | Christianity |  |  |  |  |  |  |
| **MARSTS** |  |  |  |  |  | Married |  |  |  |
| **MARSTS** |  |  |  |  |  | Single |  |  |  |
| **MARSTS** |  |  |  |  |  | Widowed |  |  |  |
| **MARSTS** |  |  |  |  |  | Divorcedo |  |  |  |
| **EDC** |  |  |  |  |  |  | None |  |  |
| **EDC** |  |  |  |  |  |  | Secondary |  |  |
| **EDC** |  |  |  |  |  |  | Quranic |  |  |
| **EDC** |  |  |  |  |  |  | Tertiary |  |  |
| **EDC** |  |  |  |  |  |  | Primary |  |  |
| **OCC** |  |  |  |  |  |  |  | Unemployed |  |
| **OCC** |  |  |  |  |  |  |  | Trader |  |
| **OCC** |  |  |  |  |  |  |  | Artisan |  |
| **OCC** |  |  |  |  |  |  |  | Entrepreneur |  |
| **OCC** |  |  |  |  |  |  |  | Civil servant |  |

| **Solutions for Fixed Effects** | | | | | | | | | |
| --- | --- | --- | --- | --- | --- | --- | --- | --- | --- |
| **Effect** | **Planned Arm** | **Age Group** | **Religion** | **Race** | **Sex** | **Marital Status** | **Education** | **Occupation** | **Estimate** |
| **Intercept** |  |  |  |  |  |  |  |  | 1.3329 |
| **ARM** | MOG |  |  |  |  |  |  |  | -0.4326 |
| **ARM** | COG |  |  |  |  |  |  |  | 0 |
| **VISIT** |  |  |  |  |  |  |  |  | -0.05977 |
| **VISIT*ARM** | MOG |  |  |  |  |  |  |  | -0.00336 |
| **VISIT*ARM** | COG |  |  |  |  |  |  |  | 0 |
| **RACE** |  |  |  | Hausa / Fulani |  |  |  |  | -0.2494 |
| **RACE** |  |  |  | Yoruba |  |  |  |  | 0.8660 |
| **RACE** |  |  |  | Igbo |  |  |  |  | -0.7394 |
| **RACE** |  |  |  | Other |  |  |  |  | 0 |
| **FAMMEM** |  |  |  |  |  |  |  |  | -1.0259 |
| **FAMMEM** |  |  |  |  |  |  |  |  | -0.9094 |
| **FAMMEM** |  |  |  |  |  |  |  |  | 0 |
| **INGR1** |  |  |  |  |  |  |  |  | -0.09671 |
| **INGR1** |  |  |  |  |  |  |  |  | -1.7061 |
| **INGR1** |  |  |  |  |  |  |  |  | 0 |
| **SEX** |  |  |  |  | Female |  |  |  | -0.7645 |
| **SEX** |  |  |  |  | Male |  |  |  | 0 |
| **AGEGR1** |  | 40-49 |  |  |  |  |  |  | -0.6967 |
| **AGEGR1** |  | 20-29 |  |  |  |  |  |  | -0.2788 |
| **AGEGR1** |  | 30-39 |  |  |  |  |  |  | -0.5646 |
| **AGEGR1** |  | 50-60 |  |  |  |  |  |  | 0.04443 |
| **AGEGR1** |  | < 20 |  |  |  |  |  |  | 0 |
| **RELG** |  |  | Islam |  |  |  |  |  | -0.1547 |
| **RELG** |  |  | Christianity |  |  |  |  |  | 0 |
| **MARSTS** |  |  |  |  |  | Married |  |  | -0.1827 |
| **MARSTS** |  |  |  |  |  | Single |  |  | -0.3421 |
| **MARSTS** |  |  |  |  |  | Widowed |  |  | -0.07256 |
| **MARSTS** |  |  |  |  |  | Divorcedo |  |  | 0 |
| **EDC** |  |  |  |  |  |  | None |  | -0.4519 |
| **EDC** |  |  |  |  |  |  | Secondary |  | -0.00268 |
| **EDC** |  |  |  |  |  |  | Quranic |  | 0.1806 |
| **EDC** |  |  |  |  |  |  | Tertiary |  | -0.03774 |
| **EDC** |  |  |  |  |  |  | Primary |  | 0 |
| **OCC** |  |  |  |  |  |  |  | Unemployed | -0.2275 |
| **OCC** |  |  |  |  |  |  |  | Trader | -0.2660 |
| **OCC** |  |  |  |  |  |  |  | Artisan | -0.08306 |
| **OCC** |  |  |  |  |  |  |  | Entrepreneur | 0.1402 |
| **OCC** |  |  |  |  |  |  |  | Civil servant | 0 |

| **Solutions for Fixed Effects** | | | | | | | | | | |
| --- | --- | --- | --- | --- | --- | --- | --- | --- | --- | --- |
| **Effect** | **Planned Arm** | **Age Group** | **Religion** | **Race** | **Sex** | **Marital Status** | **Education** | **Occupation** | **Standard Error** | **DF** |
| **Intercept** |  |  |  |  |  |  |  |  | 2.2359 | 151 |
| **ARM** | MOG |  |  |  |  |  |  |  | 0.5316 | 175 |
| **ARM** | COG |  |  |  |  |  |  |  | . | . |
| **VISIT** |  |  |  |  |  |  |  |  | 0.07083 | 175 |
| **VISIT*ARM** | MOG |  |  |  |  |  |  |  | 0.1016 | 175 |
| **VISIT*ARM** | COG |  |  |  |  |  |  |  | . | . |
| **RACE** |  |  |  | Hausa / Fulani |  |  |  |  | 0.7345 | 175 |
| **RACE** |  |  |  | Yoruba |  |  |  |  | 0.8295 | 175 |
| **RACE** |  |  |  | Igbo |  |  |  |  | 0.9325 | 175 |
| **RACE** |  |  |  | Other |  |  |  |  | . | . |
| **FAMMEM** |  |  |  |  |  |  |  |  | 0.5941 | 175 |
| **FAMMEM** |  |  |  |  |  |  |  |  | 0.5531 | 175 |
| **FAMMEM** |  |  |  |  |  |  |  |  | . | . |
| **INGR1** |  |  |  |  |  |  |  |  | 0.8003 | 175 |
| **INGR1** |  |  |  |  |  |  |  |  | 1.0531 | 175 |
| **INGR1** |  |  |  |  |  |  |  |  | . | . |
| **SEX** |  |  |  |  | Female |  |  |  | 0.6209 | 175 |
| **SEX** |  |  |  |  | Male |  |  |  | . | . |
| **AGEGR1** |  | 40-49 |  |  |  |  |  |  | 1.5961 | 175 |
| **AGEGR1** |  | 20-29 |  |  |  |  |  |  | 1.5319 | 175 |
| **AGEGR1** |  | 30-39 |  |  |  |  |  |  | 1.5093 | 175 |
| **AGEGR1** |  | 50-60 |  |  |  |  |  |  | 1.6805 | 175 |
| **AGEGR1** |  | < 20 |  |  |  |  |  |  | . | . |
| **RELG** |  |  | Islam |  |  |  |  |  | 0.5669 | 175 |
| **RELG** |  |  | Christianity |  |  |  |  |  | . | . |
| **MARSTS** |  |  |  |  |  | Married |  |  | 0.6097 | 175 |
| **MARSTS** |  |  |  |  |  | Single |  |  | 0.8461 | 175 |
| **MARSTS** |  |  |  |  |  | Widowed |  |  | 0.6944 | 175 |
| **MARSTS** |  |  |  |  |  | Divorcedo |  |  | . | . |
| **EDC** |  |  |  |  |  |  | None |  | 0.8699 | 175 |
| **EDC** |  |  |  |  |  |  | Secondary |  | 0.7479 | 175 |
| **EDC** |  |  |  |  |  |  | Quranic |  | 0.8078 | 175 |
| **EDC** |  |  |  |  |  |  | Tertiary |  | 0.8456 | 175 |
| **EDC** |  |  |  |  |  |  | Primary |  | . | . |
| **OCC** |  |  |  |  |  |  |  | Unemployed | 0.7829 | 175 |
| **OCC** |  |  |  |  |  |  |  | Trader | 0.7529 | 175 |
| **OCC** |  |  |  |  |  |  |  | Artisan | 0.7784 | 175 |
| **OCC** |  |  |  |  |  |  |  | Entrepreneur | 0.8360 | 175 |
| **OCC** |  |  |  |  |  |  |  | Civil servant | . | . |

| **Solutions for Fixed Effects** | | | | | | | | | | |
| --- | --- | --- | --- | --- | --- | --- | --- | --- | --- | --- |
| **Effect** | **Planned Arm** | **Age Group** | **Religion** | **Race** | **Sex** | **Marital Status** | **Education** | **Occupation** | **t Value** | **Pr > \|t\|** |
| **Intercept** |  |  |  |  |  |  |  |  | 0.60 | 0.5520 |
| **ARM** | MOG |  |  |  |  |  |  |  | -0.81 | 0.4169 |
| **ARM** | COG |  |  |  |  |  |  |  | . | . |
| **VISIT** |  |  |  |  |  |  |  |  | -0.84 | 0.3999 |
| **VISIT*ARM** | MOG |  |  |  |  |  |  |  | -0.03 | 0.9737 |
| **VISIT*ARM** | COG |  |  |  |  |  |  |  | . | . |
| **RACE** |  |  |  | Hausa / Fulani |  |  |  |  | -0.34 | 0.7346 |
| **RACE** |  |  |  | Yoruba |  |  |  |  | 1.04 | 0.2979 |
| **RACE** |  |  |  | Igbo |  |  |  |  | -0.79 | 0.4289 |
| **RACE** |  |  |  | Other |  |  |  |  | . | . |
| **FAMMEM** |  |  |  |  |  |  |  |  | -1.73 | 0.0860 |
| **FAMMEM** |  |  |  |  |  |  |  |  | -1.64 | 0.1020 |
| **FAMMEM** |  |  |  |  |  |  |  |  | . | . |
| **INGR1** |  |  |  |  |  |  |  |  | -0.12 | 0.9040 |
| **INGR1** |  |  |  |  |  |  |  |  | -1.62 | 0.1070 |
| **INGR1** |  |  |  |  |  |  |  |  | . | . |
| **SEX** |  |  |  |  | Female |  |  |  | -1.23 | 0.2199 |
| **SEX** |  |  |  |  | Male |  |  |  | . | . |
| **AGEGR1** |  | 40-49 |  |  |  |  |  |  | -0.44 | 0.6630 |
| **AGEGR1** |  | 20-29 |  |  |  |  |  |  | -0.18 | 0.8558 |
| **AGEGR1** |  | 30-39 |  |  |  |  |  |  | -0.37 | 0.7088 |
| **AGEGR1** |  | 50-60 |  |  |  |  |  |  | 0.03 | 0.9789 |
| **AGEGR1** |  | < 20 |  |  |  |  |  |  | . | . |
| **RELG** |  |  | Islam |  |  |  |  |  | -0.27 | 0.7853 |
| **RELG** |  |  | Christianity |  |  |  |  |  | . | . |
| **MARSTS** |  |  |  |  |  | Married |  |  | -0.30 | 0.7648 |
| **MARSTS** |  |  |  |  |  | Single |  |  | -0.40 | 0.6865 |
| **MARSTS** |  |  |  |  |  | Widowed |  |  | -0.10 | 0.9169 |
| **MARSTS** |  |  |  |  |  | Divorcedo |  |  | . | . |
| **EDC** |  |  |  |  |  |  | None |  | -0.52 | 0.6041 |
| **EDC** |  |  |  |  |  |  | Secondary |  | -0.00 | 0.9971 |
| **EDC** |  |  |  |  |  |  | Quranic |  | 0.22 | 0.8233 |
| **EDC** |  |  |  |  |  |  | Tertiary |  | -0.04 | 0.9645 |
| **EDC** |  |  |  |  |  |  | Primary |  | . | . |
| **OCC** |  |  |  |  |  |  |  | Unemployed | -0.29 | 0.7717 |
| **OCC** |  |  |  |  |  |  |  | Trader | -0.35 | 0.7243 |
| **OCC** |  |  |  |  |  |  |  | Artisan | -0.11 | 0.9151 |
| **OCC** |  |  |  |  |  |  |  | Entrepreneur | 0.17 | 0.8670 |
| **OCC** |  |  |  |  |  |  |  | Civil servant | . | . |

| **Type III Tests of Fixed Effects** | | | | |
| --- | --- | --- | --- | --- |
| **Effect** | **Num DF** | **Den DF** | **F Value** | **Pr > F** |
| **ARM** | 1 | 175 | 0.66 | 0.4169 |
| **VISIT** | 1 | 175 | 1.46 | 0.2281 |
| **VISIT*ARM** | 1 | 175 | 0.00 | 0.9737 |
| **RACE** | 3 | 175 | 1.49 | 0.2188 |
| **FAMMEM** | 2 | 175 | 2.01 | 0.1365 |
| **INGR1** | 2 | 175 | 2.15 | 0.1200 |
| **SEX** | 1 | 175 | 1.52 | 0.2199 |
| **AGEGR1** | 4 | 175 | 0.25 | 0.9090 |
| **RELG** | 1 | 175 | 0.07 | 0.7853 |
| **MARSTS** | 3 | 175 | 0.06 | 0.9799 |
| **EDC** | 4 | 175 | 0.17 | 0.9551 |
| **OCC** | 4 | 175 | 0.10 | 0.9819 |

| **Estimates** | | | | | | | | | | | | |
| --- | --- | --- | --- | --- | --- | --- | --- | --- | --- | --- | --- | --- |
| **Label** | **Estimate** | **Standard Error** | **DF** | **t Value** | **Pr > \|t\|** | **Alpha** | **Lower** | **Upper** | **Mean** | **Standard Error Mean** | **Lower Mean** | **Upper Mean** |
| **Treatment effect overtime** | -0.00336 | 0.1016 | 175 | -0.03 | 0.9737 | 0.05 | -0.2039 | 0.1972 | 0.4992 | 0.02540 | 0.4492 | 0.5491 |

| **ARM Least Squares Means** | | | | | | | | | | | | |
| --- | --- | --- | --- | --- | --- | --- | --- | --- | --- | --- | --- | --- |
| **Planned Arm** | **Estimate** | **Standard Error** | **DF** | **t Value** | **Pr > \|t\|** | **Alpha** | **Lower** | **Upper** | **Mean** | **Standard Error Mean** | **Lower Mean** | **Upper Mean** |
| MOG | -1.6235 | 0.5702 | 175 | -2.85 | 0.0049 | 0.05 | -2.7489 | -0.4981 | 0.1647 | 0.07846 | 0.06015 | 0.3780 |
| COG | -1.1808 | 0.5822 | 175 | -2.03 | 0.0440 | 0.05 | -2.3298 | -0.03183 | 0.2349 | 0.1046 | 0.08868 | 0.4920 |

| **Differences of ARM Least Squares Means** | | | | | | | | | |
| --- | --- | --- | --- | --- | --- | --- | --- | --- | --- |
| **Planned Arm** | **Planned Arm** | **Estimate** | **Standard Error** | **DF** | **t Value** | **Pr > \|t\|** | **Alpha** | **Lower** | **Upper** |
| MOG | COG | -0.4427 | 0.4522 | 175 | -0.98 | 0.3289 | 0.05 | -1.3351 | 0.4497 |
