# Supplementary material for: A double-blind, randomized controlled trial to examine the effect of Moringa oleifera leaf powder supplementation on the immune status and anthropometric parameters of adult HIV patients on antiretroviral therapy in a resource-limited setting
Source: PLoS One. 2021 Dec 31;16(12):e0261935. doi: 10.1371/journal.pone.0261935 (PMC8722362; doi:10.1371/journal.pone.0261935)
Supplement: S4 File — (DOCX) [file pone.0261935.s005.docx]

**RESEARCH PROPOSAL**

**EVALUATION OF THE ADDITION OF MORINGA OLEIFERA AS A NUTRITIONAL SUPPLEMENT ON THE ANTHROPOMETRIC, VIRAL LOAD AND CD4 COUNTS OF ADULT HIV PATIENTS ON ANTIRETROVIRAL THERAPY IN NIGERIA**

**Aisha Gambo: 213574503**

**Supervisor: Prof Indres Moodley**

***School of Public Health Medicine, College of Health Sciences,***

***Howard College Campus, University of Kwazulu-Natal, Durban, South Africa***

**Introduction**

Human Immunodeficiency Virus (HIV) infection and Acquired Immune Deficiency Syndrome (HIV and AIDS) is a serious global health issue, with more than 300 people being newly infected with the HIV every hour ([Biswas, 2012](#_ENREF_8)). Of the 35 million people living with HIV global, 25.8 million are in sub- Saharan Africa, the region most affected by the epidemic ([UNAIDS, 2015](#_ENREF_63)). Nigeria with a projected estimate of 3,229,757 people has the second largest population of people living with HIV and AIDS, second only to South Africa ([UNAIDS Global Report., 2013](#_ENREF_64)). There were 220,394 new HIV infections reported for 2013 a total of 210,031 people died from AIDS related cases ([Nigeria GARPR, 2014](#_ENREF_40)). Considerable progress has been made in providing global access to antiretroviral therapy (ART), with over three million people currently on ART worldwide ([UNAIDS, 2013](#_ENREF_62)). ART has proved efficacious in reducing morbidity and mortality related to HIV infection through the reduction in HIV viral load, an improved CD4 level which is associated with a decreased episodes of opportunistic infections ([Contri et al., 2011](#_ENREF_13)). However, improved nutrition may enhance the effectiveness of ART in infected adults and children ([Blaise Savadogo et al., 2013](#_ENREF_10)). Disease severity indicators may be directly modified by adequate nutrient intake as recommended by recent reports ([Frank et al., 2014](#_ENREF_24)). Due to widespread of food insecurity and malnutrition which is endemic in resource poor countries of Africa and Asia where most of the world’s HIV- infected people live ([Swaminathan et al., 2010](#_ENREF_54)) adequate nutrition is often not available.

Malnutrition is defined as either under- or over- nutrition and it is a threat to global public health ([Tathiah et al., 2013](#_ENREF_57)). Under-nutrition is defined as the inability of the body to consume sufficient energy, protein and micronutrients to meet its basic requirements for optimal growth and development, Under-nutrition generally manifests as underweight (low weight- for- age), wasting (low weight- for- height) and stunting (low height- for-age). Over-nutrition manifests as overweight (excessive weight relative to height) and as obesity ( excessive body fat content) ([Reddy et al., 2008](#_ENREF_47)). Malnutrition is a common manifestation among people (adults and children) infected with HIV and AIDS ([Swaminathan et al., 2010](#_ENREF_54)) and is associated with nutritional deficiencies that influences the progression of disease, increases morbidity and lowers chances of survival ([Anabwani and Navario, 2005](#_ENREF_3); [Kimani-Murage et al., 2011](#_ENREF_28)). Additionally, malabsorption in patients with chronic HIV disease may increase loss of nutrients, and patients may also experience secondary infections, loss of appetite, and increased energy expenditures, all of which contribute to poor nutritional status. The nutritional status of children infected with HIV is an important predictor of their immunological status ([McHenry et al., 2014](#_ENREF_33)). Furthermore, under-nutrition challenges the survival, growth and optimal development of children and women. It is a risk factor for poor cognitive development, reduced human capacity, premature death and other health consequences thereby reducing the strength and capacity of nations. Under-nutrition significantly hinders socio-economic development and the potential to reduce poverty ([Black et al., 2013](#_ENREF_9)). Throughout the world, the intensity of maternal and child under-nutrition remains unacceptably high ([Black et al., 2013](#_ENREF_9)). In South Africa about 35 per cent of total population lack food security. These high levels of food insecurity at household level is a vulnerability aggravated by the burden of HIV and AIDS ([Kimani-Murage et al., 2011](#_ENREF_28)). In Nigeria, the prevalence of malnutrition is high. A recent study among Nigerian men and women has shown that mortality rates among chronically energy deficient individuals who are mildly, moderately, and severely underweight are 40%, 140%, and 150% greater respectively than rates among non- chronically energy deficient (CED) individuals ([Rotimi et al., 1999](#_ENREF_48)). A study conducted in Kano, North-west region of Nigeria suggests that about 7.8% of children attending selected hospitals with mal-nutrition are HIV positive ([Sudawa et al., 2013](#_ENREF_52)).This confirmed that HIV and AIDS epidemic in Kano which has high poverty levels particularly at rural areas ([Sudawa et al., 2013](#_ENREF_52)) is still a public health problem of enormous magnitude that deserves priority attention. Kano, the commercial capital of Northern Nigeria has 9 million inhabitants and a HIV sero-prevalence of 3.4% ([Nigeria GARPR, 2014](#_ENREF_40)). Based on the aforementioned challenges, it is evident that malnutrition in people infected with HIV is a global challenge and that effective nutritional supplementation strategies are essential for enhancing the effectiveness of ART to improve the quality of lives of those people living with HIV and AIDS to enable them to realise their full potential.

**Role of Nutritional Supplementation in People Living with HIV and AIDS**

The nutritional status of people infected with HIV-1 has been extensively studied because of its influence on both humoral and cell-mediated immune function ([Nunnari et al., 2012](#_ENREF_41)). The deficiencies of vitamins and both micro and macronutrients are likely to co-exist among HIV-infected people. Micronutrients are important for maintaining optimal functioning of immunological function, reduction of oxidative stress and other metabolic processes ([Mda et al., 2010](#_ENREF_34)). Micronutrient supplementation in the diet of HIV-infected children was reported to decrease diarrhoea-associated morbidity ([Mda et al., 2010](#_ENREF_34)), improve child growth ([Steenkamp et al., 2009](#_ENREF_51)), reduce risks of respiratory infections ([Mda et al., 2010](#_ENREF_34)) and reduce all cause of mortality ([Nunnari et al., 2012](#_ENREF_41)).

Several studies have reported on the effects of different micronutrient supplementation on the immune and nutritional status of HIV- infected people. Zinc and vitamin A play a prominent role in maintaining cellular integrity ([Ndeezi et al., 2010](#_ENREF_38)). Vitamin A has been reported to have significant immunoregulatory properties on monocycte differentiation and function and also has been shown to increase the lymphocyte count, particularly the CD4+ subset, improve natural killer cell toxicity and improve the maintenance of epithelial integrity ([Nunnari et al., 2012](#_ENREF_41)). Deficiencies in antioxidants during HIV infection may facilitate disease progression by contributing to immune dysregulation and viral replication. Selenium and zinc are the key trace elements which serve as antioxidants and plays a vital role in disease progression among people infected with HIV-1 ([Bunupuradah et al., 2012](#_ENREF_11)). Selenium has been reported to play an important role in both cell-mediated and humoral immune responses. Selenium supplementation was reported to prevent to some extent morbidities in people infected with HIV-1 ([Nunnari et al., 2012](#_ENREF_41)). Other vitamins and micronutrients that play vital roles include vitamins E, B_12_, C, folic acid and iron. Reports from literature on role of nutrients in relation to adult HIV disease progression includes a randomized trial by ([Fawzi et al., 2004](#_ENREF_22)) to examine the effect of micronutrients supplements on the risks of clinical disease progression, HIV- related complications, CD4 cell counts and viral load in HIV – positive women in Tanzania. The results suggests multivitamin supplements delays the progression of the disease and provides an effective, low-cost means of delaying the initiation of antiretroviral therapy in these women. Several researches have reported on the role of vitamins and micronutrients on disease progression and mortality of children infected with HIV. These studies include a cross sectional baseline survey conducted in Uganda to determine the serum zinc status in HIV infected children and factors associated with zinc deficiency. The weight and height, HIV and AIDS clinical disease staging and absolute CD4 cell count were recorded. The survey concluded that low zinc status coupled with already existing poor nutritional and immunological status especially among infected children not on HAART could be a prominent contributor to increased morbidity. In a study conducted in South Africa by ([Krugera et al., 2013](#_ENREF_29)), the longitudinal association between dietary iron intake, haemoglobin concentration and the dietary factors associated with anaemia and iron deficiency in HIV-infected children receiving first-line HAART were examined. The haemoglobin concentration, CD4 cell count, viral load and anthropometric parameters assessed pointed to a need for low-dose iron supplementation for iron-deficient HIV-infected children because dietary iron intake was insufficient to prevent anaemia and suggested interventions to increase animal protein intake ([Krugera et al., 2013](#_ENREF_29)).

Nutritional interventions with macronutrient supplementation in HIV- infected people is essential in meeting additional energy needs for those with BMI < 18.5 and for malnourished children as recommended by the WHO ([WHO., 2008](#_ENREF_69)) to improve their nutritional status and thus delay disease progression. Various approaches have been implemented for macronutrient supplementation such as the use of supplemental formulas that include hydrolysed or elemental protein or special protein formulas with glutamine, whey ([Jochum et al., 1995](#_ENREF_27)); lipids such as in fish oil or other (n-3) fatty acid sources; and carbohydrate supplement ([Sztam et al., 2010](#_ENREF_55)). Other studies have evaluated the effect of macronutrients in HIV care in the forms of corn and soy flour porridge ([Flax et al., 2010](#_ENREF_23); [Matilsky et al., 2009](#_ENREF_31)), and other ready- to- use foods ([Ndekha et al., 2005](#_ENREF_39)) including food supplementation ([Tofail et al., 2008](#_ENREF_61)). A study reported by ([Swaminathan et al., 2010](#_ENREF_54)) assessed the effects of a six month intervention of an energy-dense macronutrient supplement on the anthropometric, body composition and immunologic status of HIV- infected adults at different stages of disease. The study showed that there was a non-significant increase in weight among patients with moderately advanced HIV disease.

The researchers pointed to the fact that the patients most likely to benefit from the supplement were not included in the study which could be the reason for the lack of noticeable effect. These patients are those that were severely ill, those who were about to initiate ART, or who required hospitalization as stated in the exclusion criteria of the study. However, the impact of nutritional rehabilitation with *Spirulina platensis* ,a blue-green alga with very high protein content was assessed in sub Saharan Africa and an improved immune status with a consequent drop in viral load was reported ([Azabji-Kenfack et al., 2011](#_ENREF_7)).

The World Health Organization (WHO) encouraged the use of Ready to Use Therapeutic Foods (RUTF) for community-based treatment of severe under-nutrition to improve and maintain better nutritional status ([Sunguya et al., 2012](#_ENREF_53); [UNICEF., 2013](#_ENREF_66)). RUTF is an energy dense lipid paste made of peanut butter, milk powder, oil, sugar, mineral, vitamins and protein mix ([Sunguya et al., 2012](#_ENREF_53)). RUTF does not need any further preparation or the addition of water before consumption and can be kept for long periods without refrigeration ([Wiles et al., 2014](#_ENREF_70)). ([Sunguya et al., 2012](#_ENREF_53)) reported that there was a positive association of RUTF intervention with stunting, wasting and underweight statuses among ART- treated children in Dar es salaam. The study concluded that RUTF has the potential to improve under-nutrition among HIV- positive children on ART. The study therefore emphasized the popularity of RUTF in feeding program interventions and its increased successful rates for treatment of severe malnutrition. However the use of supplements such as RUTF might be of disadvantage in terms of acceptability, high price and low regional availability ([Dibari et al., 2013](#_ENREF_16); [Sztam et al., 2013](#_ENREF_56)). Therefore a product that is novel, cheaper, culturally acceptable, efficacious and regionally produced could be a solution in the management of malnutrition ([Dibari et al., 2013](#_ENREF_16)) such as adding the leaves or powder of Moringa oleifera, locally grown to supplement local diets ([Thurber and Fahey, 2009](#_ENREF_60)). Nutritional supplementation with Moringa oleifera could be more sustainable than imported supplements in developing countries like Nigeria due to lower cost of transport and processing ([Sztam et al., 2013](#_ENREF_56)).

**Moringa Oleifera Nutritional Supplement**

Moringa oleifera Lam (syn. M. ptreygosperma Gaertn.) is one of the best known and most widely distributed and naturalized species of a monogeneric family Moringaceae ([Anwar et al., 2007](#_ENREF_5)) and its currently found in many parts of the world. In Nigeria, it is found in the Northern, Southern, Western and Eastern parts of the country. Moringa oleifera is also known as the horseradish tree or drumstick tree ([Fahey, 2005](#_ENREF_18)). In Nigeria, it is known as “Zogale” in Hausa, “Gawara”, in Fulani, “Okwe Oyibo” in Igbo, and “Ewe Igbale” in Yoruba ([Williams et al., 2013](#_ENREF_71)). In many countries particularly in India, Pakistan and many parts of Africa, the leaves, fruits, flowers and immature pods of Moringa oleifera tree are used as a highly nutritious vegetable ([Anwar et al., 2007](#_ENREF_5)). Its nutrient-dense leaves are high in protein quality ([Thurber and Fahey, 2009](#_ENREF_60)). The medicinal uses of Moringa oleifera arises from the fact that the entire plant has high protein, vitamins, mineral, and carbohydrate content. It is, therefore of high nutritional value for both humans and livestock ([Asiedu-Gyekye et al., 2014](#_ENREF_6)). The leaves of Moringa oleifera have been reported to be a valuable source of both macro- and micronutrients ([Oduro et al., 2008](#_ENREF_42)) and also acts as a good source of natural antioxidant due to presence of various types of natural antioxidant compounds such as ascorbic acid, flavonoids, phenolics and carotenoids ([Anwar et al., 2007](#_ENREF_5)). ([Asiedu-Gyekye et al., 2014](#_ENREF_6)) reported a study in which X-ray fluorescence (XRF) analytical technique was used for the elemental and chemical analysis of powdered, solid, and liquid samples of Moringa oleifera dried leaves. The analyses produced a total of 35 elements (14 macroelements and 21 microelements). The macroelements in the powdered leaf samples includes S, Ca, K, Mg, Na, P, Si, Cl, Al, Fe, and Mn. The minor elements produced by the analyses in the decreasing order are V, Ba, Cr, Y, Ba, Zn, Rb, Ce, La, Cu, Cs, Sn, Co, Ni, and Zr. The concentrations of all the elements were within the recommended daily allowance (RDA) limits.

For hundreds of years, traditional healers have prescribed different parts of Moringa oleifera for treatment of skin diseases, respiratory illnesses, ear and dental infections, hypertension, diabetes, cancer treatment, water purification and as nutrient supplementation ([Anwar et al., 2007](#_ENREF_5); [Fahey, 2005](#_ENREF_18)). In a review by ([Modi et al., 2010](#_ENREF_35)) various studies on the pharmacological activities and clinical trials on Moringa oleifera plant were reported. Some of these pharmacological activities includes anti- anaphylactic activities, anti-arthritic properties, anti-atherosclerotic, antibacterial and anticancer activities.

Africa and Asia which are the regions with high prevalence of under-nutrition and where most of the world’s HIV- infected people live ([Swaminathan et al., 2010](#_ENREF_54)) all share the ability to grow and utilize Moringa oleifera ([Thurber and Fahey, 2009](#_ENREF_60)). In view of the widespread use of Moringa oleifera leaf powder as a food supplement and treatment for various disease conditions, ([Asiedu-Gyekye et al., 2014](#_ENREF_6)) set out to determine if a 14-day dosing of Moringa oleifera collected in Accra, Ghana could have any adverse effects in rats . The study reported that the consumption of Moringa oleifera is reasonably safe taking into account the elemental composition when administered to rats. The study further recommends that the consumption of Moringa oleifera leaves be limited to a maximum of 70grams per day so as to prevent excessive consumption and subsequent accumulation of some of the essential elements. A recent study was conducted at La-Bio Research cc with the aim to determine the oral toxicity of Moringa oleifera dried leaf powder in Balbc mice following the OECD guidelines for testing sub chronic toxicity in animals. In this repeated dose 90-day oral toxicity study, no adverse effects up to 1000 mg/kg/day of Moringa oleifera dried leaf powder over a prolonged chronic exposure period of 90 days ([La-Bio Research cc., 2015](#_ENREF_30)) were observed.

In a randomized study, ([Idohou-Dossou et al., 2011](#_ENREF_26)) reported the impact of daily consumption of Moringa oleifera leaf powder on iron status and weight gain in Senegalese lactating women. The study reported a low bioavailability of the iron in Moringa oleifera that could not cover the iron requirements in the lactating women. Another study on nutritional development on HIV patients ([Tété–Bénissan et al., 2013](#_ENREF_58)) evaluated the mineral composition of “Togolese ecotype” of Moringa oleifera leaves and its effect on anthropometric parameters, atherogenic lipids and glycaemia during nutritional recovery in HIV negative and HIV positive malnourished patients in Togo after daily use of the leaves powder. The study reported significant decrease of serum levels of total cholesterol, triglycerides, LDL-C correlated with significant increase in HDL-C. These results confirm Moringa oleifera leaves potential as hypolipidemic, hypocholesterolemic agent which induced the decrease of atherogenic lipids. A reduction of the glycaemia values among HIV positive and HIV negative patients was also reported . Similarly, ([Rajanandh et al., 2012](#_ENREF_46)) evaluated the leaves of Moringa oleifera for its hypolipidemic. antioxidant, anticoagulant, platelet antiaggregatory and anti-inflammatory activity in experimental animals. The results reported the therapeutic potential of the hydroalcoholic extract of Moringa oleifera against vascular damage and atherogenesis that leads to various types of cardiovascular complications. Furthermore, the study suggests that Moringa oleifera can be used by patients with coronary artery disease along with their regular medicine.

([Monera and Maponga, 2012](#_ENREF_36)) conducted a cross-sectional survey to determine the prevalence and patterns of Moringa oleifera use by HIV positive people in Harare, Zimbabwe. The study established evidence of its use as a nutritional supplementation among HIV positive patients as a large percentage of the study participants that have commenced antiretroviral therapy consume Moringa oleifera. The study also reported that friends or relatives were the most common source of a recommendation for use of Moringa oleifera to the HIV patients. Also in Oyo State in Nigeria ([Osewa et al., 2013](#_ENREF_44)) reported a study to determine the perception of rural dwellers on the nutritional and medicinal values of Moringa oleifera. The study reported that the health benefit of Moringa is significant. Majority of the respondents are aware that Moringa leaves has highly protein content and rich in vitamins and minerals. The respondents had access to information through friends or relatives on a regular basis on the utilization of Moringa. The leaves are either taken fresh, dried and cooked. The study also reported that Moringa does not have any religious taboos while its acceptability cuts across both religious and cultural beliefs.

([Monera et al., 2008](#_ENREF_37)) reported the *in vitro* CYP3A4 inhibitory activity by Moringa oleifera leaf extracts suggesting potential of interaction with antiretroviral drugs. However,no adverse effects have been reported despite the wide spread and concomitant use of Moringa oleifera with antiretroviral drugs in sub-saharan Africa especially in Nigeria.

Despite widespread knowledge and reported benefits of Moringa oleifera, few well controlled scientific studies have been reported on the benefits of Moringa oleifera in patients with nutritional deficiencies. Moreover, there is a paucity of scientifically robust studies on effects of Moringa oleifera on the immunological status of people infected with HIV and AIDS.

**Conceptual Framework**

Food insecurity, defined as having uncertain or limited availability of nutritionally adequate or safe food or the inability to acquire personally acceptable foods in socially acceptable ways, is a leading cause of morbidity and mortality worldwide and is inextricably linked to the HIV epidemic. Food insecurity and HIV and AIDS may be linked at the community, household, and individual levels and be intertwined in a vicious cycle through nutritional, mental health, and behavioural pathways. Specifically, food insecurity can lead to macronutrient and micronutrient deficiencies, which can contribute to immunologic decline and increased morbidity and mortality among those already infected ([Weiser et al., 2011](#_ENREF_67)).

**Research Hypothesis**

We hypothesize that Moringa oleifera as a food supplement for HIV-infected patients on antiretroviral therapy can improve their nutritional and immunological status.

**Aims and Objectives of Study**

The aim of the study is to evaluate the effect of six months Moringa oleifera supplementation on the nutritional and immunological development of HIV positive adults on antiretroviral therapy. The study will further assess the impact of Moringa oleifera supplementation on quality of life (QoL) in patients with HIV and AIDS on ART drugs.

.The objectives are:

1. The nutritional development will be evaluated by determining the changes in anthropometric parameters (height, weight and mid arm circumference) of HIV patients on ARV before and after Moringa oleifera supplementation.
2. The effect of Moringa oleifera supplementation on the viral load and CD4 cell counts will be observed to evaluate immunological development
3. To assess the quality of life (QoL) in patients by using the template of the WHOQoL-HIV BREF questionnaire on Moringa oleifera supplementation in comparison with a matched control group

**Study Design**

**Setting**

The location of the study will be the S. S Wali Virology centre at the Aminu Kano Teaching Hospital, Kano state (AKTH) Nigeria.

AKTH is a tertiary health institution that operates a daily HIV clinic and also serves as a referral centre for three states in North Western Nigeria. It also serves as a centre for clinical examinations, laboratory tests, HIV screening, counselling, treatment and distribution of free antiretroviral drugs provided by the Federal Government and the Institute of Human Virology, Nigeria (IHVN) in partnership with its global partners which includes., the Centres for Disease Control and prevention (CDC) and the Global Fund to Fight AIDS, Tuberculosis and Malaria. The centre attends to all patients with HIV infection diagnosed within the hospital or referred from outside the health facility.

**Subjects/ Participants**

The study is a randomized controlled intervention study.

The study population will be all HIV and AIDS patients in Kano and the neighbouring states attending the HIV clinic at AKTH.

The study sample will be male and female adults 18 yrs and above who presented at the centre with a previous or new diagnosis of HIV infection and had commenced ARV therapy for at least three months.

The target population is adults and will be able to give informed consent to participate in the study.

Participants will be recruited for the study using convenience sampling technique. As participants present themselves to the clinic for HIV services offered, they will be approached for participation in this study. Following written consent, participants who fulfil the inclusion criteria will be enrolled consecutively until the required sample size is reached. Recruitment will be done by HIV clinic staff responsible for booking patients’ appointments either for doctor’s consultation, drug refills at the pharmacy, laboratory services or any HIV related services offered.

**Randomization procedure**

Block randomization will be used to balance the groups throughout the enrolment period. Blocked randomization offers a simple means to achieve balance between study arms and to reduce the opportunity for bias and confounding. PASS 12.0 software will be used to develop the randomisation list using Wei's Urn algorithm. Furthermore we will check that randomization was successful at completion of enrolment i.e. that no significant differences in the socio-demographic and/or other characteristics were found between the two arms. According to the statistitian, a sample of 86 patients in each arm will be required i.e. total sample size of 172 which will be rounded up to 200 i.e 100 patients in each arm.

Inclusion criteria for the study are:

1. Being HIV sero-positive
2. Patients ≥ 18 years old
3. Patients with CD4 counts ≤ 500 cells/mm^3^
4. Commenced ART at least for three months (Tenofovir + Lamivudine + Efavirenz combination).
5. Be willing to give informed consent and comply with study protocol

Exclusion criteria for the study are:

1. Known allergy or intolerance to Moringa oleifera or placebo (starch powder)
2. Pregnant women
3. Patients with CD4 counts > 500 cells/mm^3^
4. Patients with active opportunistic infection
5. Patients taking micronutrient or natural health product supplements within 30days of randomization
6. Participation in a clinical study of any investigational product 1 month prior to visit 1 or during the study

**Sample size calculation (difference in mean weight or CD4 count by RCT arm using standard t-test)**

According to the statistician, to be able to detect a medium effect size (Cohen’s d =0.5) ([Cohen, 1988](#_ENREF_12)) or 0.5 standard deviation in mean weight or CD4 by RCT arm with 90% power (1-β [type 2 error probability]) and 95% confidence (or 5% α error probability [type 1]) assuming a balanced 1:1 study design, a sample of 86 in each group will be required i.e. total sample size of 172 which will be rounded up to 200 i.e 100 patients in each arm. The sample size was calculated using G*Power version 3.1.9.2 ([Faul et al., 2009](#_ENREF_21)).

**Study Variables**

Dependent variables:

• Nutritional status ( anthropometric parameters [ weight, height, waist circumference, BMI] )

• Immunological status (CD4, viral load)

• Occurrence and frequency of opportunistic infections

• Quality of life (QoL)

Independent variable:

• Moringa oleifera supplementation

**Data Collection Tool**

Interviewer- administered questionnaire will be used to obtain complete socio-demographic history from the patients. The anthropometric, immunological and opportunistic infection data will be obtained from patients’ hospital record. FAO/Nutrition and Consumer Protection Division recommended questionnaire for data collection on individual Dietary Diversity Score (IDDS)([FAO/Nutrition and Consumer Protection Division., version of May, 2007](#_ENREF_19)) will be used to determine nutritional status of patients. The dietary diversity questionnaire is a tool that provides a more rapid, user-friendly and cost-effective approach to measure changes in dietary quality at the household and individual level and is also a proxy of the nutrient adequacy of the diet for individuals by providing a 24 hours recall of all foods eaten.

The WHOQoL-HIV BREF questionnaire ([WHO., 2002](#_ENREF_68)) will be used to assess the quality of life of patients.

**Time Line**

|  | Last 10 days of May ‘16 | June  ‘16 | July  ‘16 | August  ‘16 | Sept  ‘16 | Oct  ‘16 | Nov  ‘16 |
| --- | --- | --- | --- | --- | --- | --- | --- |
| Capture baseline information and screening of participants |  |  |  |  |  |  |  |
| Capture data on nutritional status |  |  |  |  |  |  |  |
| Capture data on immunological status |  |  |  |  |  |  |  |
| Capture data on opportunistic infections |  |  |  |  |  |  |  |
| Capture data on Quality of Life (QoL) |  |  |  |  |  |  |  |

Gantt chart illustrating timeline for different data collection

**Data Collection Process**

1. Permission to conduct the study at AKTH Kano has been obtained but study will commence after approval is granted by the ethics committees of the hospital and UKZN.
2. Socio-demographic information: At the first visit, the researcher will interview the patients to obtain demographic, patient history and other relevant information, particularly dietary information.
3. Objective 1 will be achieved as follows: Trained clinic staffs will be responsible for all anthropometric measurements and data collection under supervision of the research team. The weight will be taken with the participants wearing light clothing and without shoes using a digital scale, height using a stadiometer and mid upper arm circumference using a steel tape. The BMI will be calculated as the weight in kilograms divided by the square of height in meters
4. Objective 2 will be achieved by: The viral load and CD4 measurements will be taken from patients records.
5. Objective 3 will be achieved as follows: The occurrence of opportunistic infections during the cause of the intervention will be obtained from patient records and the frequency noted.

To obtain the nutritional status of patients, the dietary diversity questionnaire will be used to determine the type of foods eaten in the previous 24 hours. Dietary Diversity is defined as the number of different foods or food groups consumed over a given reference period ([Ruel, 2003](#_ENREF_49)). The dietary diversity questionnaire is a tool that provides a more rapid, user-friendly and cost-effective approach to measure changes in dietary quality at the household and individual level and is also a proxy of the nutrient adequacy of the diet for individuals by providing a 24 hours recall of all foods eaten. The recall typically is conducted by interview, in person or by telephone ([Thompson and Subar, 2001](#_ENREF_59)). The FAO/Nutrition and Consumer Protection Division recommended questionnaire for data collection on individual Dietary Diversity Score, May 2007 version is a validated tool that has been used in studies in Nigeria ([Ajani, 2010](#_ENREF_1)). The use of 24-hour recall is advantageous because the tool is administered by an interviewer who records the responses therefore literacy of the respondent is not required ([Thompson and Subar, 2001](#_ENREF_59)). This factor is important in this study because of the low literacy level of participants. Also the 24-hour recall is preferred for this study compared with other dietary assessment methods e.g. food frequency questionnaire, food dairies etc that will require the participant to fill in the questionnaire at home and may compromise confidentiality. Because most individuals’ diets vary greatly from day to day, it is not appropriate to use data from a single 24-hour recall to characterize an individual’s usual diet therefore in this study we propose to use multiple 24-hour recalls. Multiple days of recalls can better assess the individual’s usual intake ([Thompson and Subar, 2001](#_ENREF_59)). Face-to-face interview will be conducted by the study nutritionist and researcher and the participants at baseline and at each monthly visit to the clinic and telephone interviews will be scheduled two times a month. For participants that do not possess telephones, alternative ways will be sought like using the telephone of a support group member. At each interview, the participants will be asked what they had eaten or drunk at specific time periods to represent the different meal types such as breakfast, lunch, dinner and snacks times. The foods and drinks mentioned will then be recorded on a 24 hour dietary recall tool in the respective time periods of the day and will be classified into the 12 food groups, namely; Cereals, Vitamin A rich vegetables and tubers, white tubers and roots, dark green leafy vegetables, other vegetables, Vitamin A rich fruits, other fruits, organ meat (iron rich), flesh meats, eggs, fish, legumes, nuts and seeds, milk and milk products, oils and fats. Each food group eaten by the participants will be given a score of 1 and the total individual scores will be computed. The total individual food score will be categorized into terciles, namely Low IDDS terciles is equivalent to low dietary diversity (1 to 3 food groups); Medium IDDS terciles equivalent 4 to 5 food groups and High IDDS terciles means 6 or more food groups.

In addition to the multiple 24-hour recalls, using the same diet diversity questionnaire, a modified 30-days food history questionnaire will also be used to record participants’ typical daily diet and the frequency of the diet. The diet diversity questionnaire scoring format will be used to allocate the scores of each food item eaten on the food history questionnaire and a comparison will be made. This will be conducted at each monthly visit to the clinic.

The patient will answer the questions on the WHOQoL- HIV BREF to determine their quality of life. The WHOQOL-HIV BREF is one of the World Health Organization’s QoL instruments for use with HIV-infected populations. It is an instrument developed to address specific aspects of the QOL for patients living with HIV and AIDS ([Hsiung et al., 2011](#_ENREF_25)).

The instrument comprises six different domains: physical, psychological, level of independence, social relationships, environment, and spirituality/religion/personal beliefs. According to the WHOQoL Group (1995), QoL assessment in different domains allows checking the dimensions in which treatments are effective, with potential to help in the decision-making about the most appropriate therapeutic measures and possibly reduce health care costs ([da Silva et al., 2013](#_ENREF_15)). The WHOQOL-HIV BREF is a validated tool that has been used in studies conducted in various countries including Nigeria ([Olisah et al., 2011](#_ENREF_43); [Peltzer et al., 2010](#_ENREF_45)). The interviewer will assist those patients not capable of filling the questionnaire themselves. At the end of the interview the total scores will be obtained to determine the patients’ quality of life.

The two data collection tools namely FAO/Nutrition and Consumer Protection Division recommended questionnaire for data collection on individual Dietary Diversity Score and the WHOQOL-HIV BREF will be translated to Hausa language which is the local language spoken in the northern region of Nigeria. For participants that understand English the English version of the questionnaire will be used. The translation of the tools will be undertaken by experts at the Department of languages, Bayero university Kano. The same institution will be responsible for the back translation of the filled questionnaires to English language.

The socio-demographic information will be obtained at baseline. All measurements of anthropometric parameters, CD4 cell counts and occurrence and frequency of opportunistic infection and nutritional history will be recorded at baseline before supplementation, during the course of study and at the end of study. The Viral load test will be done and recorded at beginning of the study and at the end after six months. Information on quality of life will be obtained at baseline and end of study at six months to determine the impact of Moringa oleifera on the different parameters.

Community consultation will be conducted throughout the life-cycle of the study following the UNAIDS/AVAC Good Preparatory Practice (GPP) guidelines for biomedical HIV prevention trials. In Aminu Kano Teaching Hospital (AKTH) as in all tertiary hospitals that render HIV and AIDS services in the nation, there is a “support group” that comprises of individuals and groups of people living with HIV and AIDS or receiving HIV care of any sort from the hospital. This support group have executive members that ultimately represent the interests of the people who would be recruited to or participate in the trial, and others locally affected by the trial. Therefore based on the GPP guidelines, the support group representative will serve as the “community stakeholder group” that will work together with the research team to build transparent, meaningful, collaborative, and mutually beneficial relationships with the ultimate goal of shaping the research collectively ([Allman et al., 2014](#_ENREF_2); [UNAIDS/AVAC, 2011](#_ENREF_65)). Because appropriate and meaningful stakeholder engagement should occur at all stages of the research life-cycle –from trial design to results dissemination, this study will be planned and conducted following GPP through the entire research life-cycle. This is expected to help facilitate local ownership of research. It also enables more equitable relationships and increases the likelihood of successful research conduct, trial completion and application of research results ([UNAIDS/AVAC, 2011](#_ENREF_65)).

The research dietician will counsel and monitor all participants on the appropriate diet to consume. Participants in the intervention group will receive Moringa oleifera supplementation together with their ART drugs while participants in the control group will receive placebo with their ART drugs. The Moringa oleifera supplement or placebo which will be coloured (green) cornstarch powder ([Cosgrove and Black, 2013](#_ENREF_14); [Seok-Jae et al., 2013](#_ENREF_50)), to be similar in look as Moringa oleifera. The placebo cornstarch powder will be coloured using food grade colour by Dala Foods Nigeria Limited. Dala Foods Nigeria Limited is a Nigerian based food processing company located in Kano state. Both the intervention and the placebo will be provided as a powder in a sealed packaged. The Moringa oleifera supplementation or placebo ( 5g or one teaspoonful) will be consumed three times daily at mealtimes by adding it to sauces or to porridges just before serving them ([Idohou-Dossou et al., 2011](#_ENREF_26); [Tété–Bénissan et al., 2013](#_ENREF_58)). Due to opportunistic infections associated with HIV infection, the intervention can also be sipped slowly as shake when patients experience difficulty in swallowing. In Kano, home visits of participants by research members to ensure compliance may not be convenient to the participants as questions may be asked by family members. Compliance will be monitored by questioning patients during their monthly visits to evaluate adherence. The diet of participants will also be monitored. Schedule monitoring will also include weekly telephone calls to the patients in the first month and biweekly thereafter. In Nigeria the use of Moringa oleifera is promoted, based on the recommendation of its nutraceutical benefits. The Nigerian Federal Government Raw Materials Research and Development Council (RMRDC) has been actively encouraging the uptake of Moringa oleifera farming and its consumption. A study by ([Williams et al., 2013](#_ENREF_71)) conducted in Kwara state, Nigeria reported an awareness of the nutritional benefits of Moringa oleifera by approximately half of the study participants and that there was a willingness to use Moringa oleifera by non-users after receiving information on its nutritional benefits. Also the studies reported by ([Animashaun et al., 2013](#_ENREF_4); [Farinola et al., 2014](#_ENREF_20)) suggested that there is an awareness of the perceived benefits of Moringa oleifera and an acceptance of its nutritional value in Nigeria. On this basis it is anticipated that adherence by the patients will be relatively high. Adherence will be monitored at each visit to the clinic when participants will be asked if they have been taking the supplement as prescribed and if there were any challenges they encountered including any adverse reactions that is different from the known adverse reactions usually experienced by HIV patients on antiretroviral therapy ([Eluwa et al., 2012](#_ENREF_17); [Max and Sherer, 2000](#_ENREF_32)) if the physician sees it fit, the participants will be terminated from the study. This study will be limited to evaluating the effect of Moringa oleifera supplementation on the nutritional development (anthropometric parameters) and immunological development (viral load and CD4 counts) of HIV positive adults on antiretroviral therapy. The study will further assess the impact of Moringa oleifera supplementation on quality of life (QoL) in patients with HIV and AIDS on ART drugs. The study will not explore drug interactions with Moringa oleifera. Although ([Monera et al., 2008](#_ENREF_37)) reported invitro CYP3A4 inhibitory activity exhibited by the leaf extracts of Moringa oleifera and the potential of Moringa oleifera interaction with antiretroviral drugs, the wide use and concomitant administration of Moringa oleifera with antiretroviral drugs in sub-saharan Africa and especially in Nigeria warrants these present study so as to scientifically prove the perceived nutraceutical benefits of Moringa oleifera to HIV patients.

Participants will be asked if family members share their supplements so that enough of the supplement can be given to ensure that participants consumed the quantity requested for the study.

The possible confounders in the study could be:

1. The different types of ART drugs combination the patients are taking: To overcome this factor the study will be conducted on HIV patients on the same ART drugs combination (Tenofovir + Lamivudine + Efavirenz). This ART combination is chosen because it is the most preferred regimen by WHO and thus more patients are on it.
2. Nature of individual normal diet: This factor will be considered by monitoring the diet of the participants during the course of the study by the study dietician and also offering dietary counselling.
3. Differences in HIV staging: Participants with CD4 counts ≤ 500 cells/mm^3^ will be used for the study to minimize variation in HIV staging.

**Study Outcomes**

The outcomes to be assessed will be changes in anthropometric parameters ( weight; body mass index [ BMI ], to be calculated as the weight in kilograms divided by the square of height in meters; and midarm circumference), immune status (CD4 cell count and viral load), occurrence and frequency of opportunistic infections and patients’ quality of life (QoL).

**Data Processing And Analysis**

Data will be processed and analysed using SPSS statistical package. Significance differences in mean CD4 count or weight (i.e. primary hypothesis) at follow-up in the two arms will be assessed using the standard t-test. If the data are not normal then the Wilcoxon rank sum test will be used instead. A repeated measure ANOVA may also be applied using the pre-post measurement design. Bivariate and multivariable adjusted mixed effects linear regression may also be used to assess the primary hypothesis. Equivalence testing may also be performed based on a clinically meaningful equivalence zone (Δ).

**Data Management**

Data entry clerks in the HIV clinic will be used to enter the data to ensure accuracy and consistency of data. Data will be captured electronically using a specialised data management software such as Epi-Info using a constraints and validation checks to reduce data entry errors or pick up errors. The data will be stored on the principal researchers’ computer while all data on hard- copies will be kept under lock. The data will be accessible only to the members of study team, the study sponsors or AKTH HIV management team. Any queries arising from missing data or data anomalies will be resolved.

**Limitations Of Study**

Patients may feel reluctant to participate in the study if there is no monetary benefits involved because most people believe such studies conducted on HIV patients are sponsored by international organisations.

**Novelty of the Study**

The study is expected to:

1. Lead to the development of a clinically evaluated affordable nutritional supplement for improved outcomes in malnourished patients with HIV and AIDS. It will increase the use of commercially available moringa nutritional supplements by HIV and AIDS patients thereby providing employment in this sector of economy of Nigeria.
2. To address the millennium development goals which have now been transformed to Sustainable Development Goals (SDGs) pertaining to malnutrition that is prevalent in sub Saharan Africa especially Nigeria thereby improving growth and development of children and adults infected with HIV and AIDS which invariably improves the strength and capacity of the nation.
3. Agro-processing industries can benefit from the study by scaling up moringa production for commercial purpose.
4. Due to its novelty, it will improve the pool of new knowledge in nutritional supplementation and HIV and AIDS therapy.
5. Because Moringa oleifera is regarded as an indigenous plant in Nigeria, the study will lead to increased cultivation of the plant by people infected with HIV and AIDS and the general population thereby improving their income status.

**Ethical Considerations**

This research protocol is compliance with the principles enunciated in the Declaration of Helsinki.

Ethical clearance: Ethical clearance and approval will be obtained from the UKZN institutional Human Research Ethics Committee and the Aminu Kano teaching hospital (AKTH) ethical committee.

To ensure confidentiality, all forms to be used for data capturing will refer only to record numbers, with no reference to the personal information of the participants.

All participants will have to provide oral or written informed consent to take part in the study and to adhere to the study protocol (see Appendix).

Participants will be compensated by reimbursing for travel to study site when they are needed for the purpose of the study on days other than their normal clinic days. No other incentives or gifts will be given for participation. Participants will be assigned codes in order to maintain their anonymity. No information regarding the participants’ involvement in this study will be shared at any point.

.

**Dissemination of the Study Results**

The study findings will be disseminated to the University of Kwazulu-Natal, Discipline of Public Health Medicine and the Aminu Kano teaching hospital HIV management team. Information generated by this study will be presented at national and international conferences and published in peer-reviewed journals. The information will also be used by the national HIV and AIDS nutritional care programs to plan appropriately for improvement of nutritional care in HIV and AID’S patients.

**Time Table**

|  | Nov  ’15- Mar ’16 | Mar  ‘16 | Apr  ‘16 | Apr ’16 | May  ‘16 | Jun  ‘16 | Jul  ‘16 | Aug  ‘16 | Sep  ‘16 | Oct  ‘16 | Nov  ‘16 | Dec  ‘16 | Jan  ‘17 | Feb ‘17 | Mar ‘17 |
| --- | --- | --- | --- | --- | --- | --- | --- | --- | --- | --- | --- | --- | --- | --- | --- |
| Application for Ethics |  |  |  |  |  |  |  |  |  |  |  |  |  |  |  |
| Literature review |  |  |  |  |  |  |  |  |  |  |  |  |  |  |  |
| Recruitment of subjects |  |  |  |  |  |  |  |  |  |  |  |  |  |  |  |
| Data capture |  |  |  |  |  |  |  |  |  |  |  |  |  |  |  |
| Data analysis |  |  |  |  |  |  |  |  |  |  |  |  |  |  |  |
| Report writing |  |  |  |  |  |  |  |  |  |  |  |  |  |  |  |
| Finalizing and dissemination of product |  |  |  |  |  |  |  |  |  |  |  |  |  |  |  |

**Budget**

| ACTIVITY | REASON | AMOUNT | TOTAL |
| --- | --- | --- | --- |
| Transport | Transport of PI to study site throughout study period | R30 to and from AKTH for 36 weeks  R 30 x 4dys x 36 wks | R 4 320.00 |
| Reimbursement | Transport of the research participants to and from study site throughout study period | R R50 x max of 6 visits x 200 participants | R 60 000.00 |
|  | Transport of members of research team (4) to and from study site throughout study period | R 30 x 4dys x 36 wks x 4 members | R 17 280.00 |
| Cost of Moringa oleifera and processing | Moringa oleifera  (investigational product) | 5g x 3 doses x 180 days x 100 participants  = 300 kg approx | R 2 800.00 |
|  | Processing of Moringa oleifera suitable for research | 300 kg approx | R 1 000.00 |
| Cost of Corn flour and food colour and cost of processing | Corn flour powder as placebo ( control) | 5g x 3 doses x 180 days x 100 participants  = 300 kg approx | R 2 200.00 |
|  | Processing of corn starch using food colour suitable for research | 300 kg approx | R 4 000.00 |
| Laboratory Investigations for participants | CD 4 cell count | R 40 x 4 investigations x 200 participants | R32 000.00 |
|  | Viral load test | R 200 x 1 investigation x 200 participants | R40 000.00 |
| Printing | Consent forms and tools | R 1 500 | R 1 500.00 |
| Binding | Projects | R 300 | R 300.00 |
| Telephone calls | To call participants | R 2 000 | R 2 000.00 |
| Computer | For on-site data capturing | R 5 500 | R 5 500.00 |
| Travel | From Nigeria to South Africa to further process and submit research findings | R 4 800 | R 4 800 |
| Miscellaneous |  | | R 2 300.00 |
| TOTAL |  | | R 180 000.00 |

**References**

Ajani, S.R., 2010. An Assessment of Dietary Diversity in Six Nigerian States. Afr J Biomed Res 13, 161-167.

Allman, D., Ditmore, M.H., Kaplan, k., 2014. Improving Ethical and Participatory Practice for Marginalized Population in Biomedical HIV Prevention Trials: Lessons from Thailand. PLOS ONE 9, 1-7.

Anabwani, G., Navario, P., 2005. Nutrition and HIV/AIDS in sub-Saharan Africa: an overview. Nutrition 21, 96-99. DOI:10.1016/j.nut.2004.1009.1013.

Animashaun, J.O., Williams, F.E., Toye, A.A., 2013. Towards Validating Moringa’s Nutraceutical Benefits: An Examination of Consumers’ Perspectives vis-à-vis Health Benefits Efficacy and Willingness to Pay. Agris on-line Papers in Economics and Informatics V, 11-21.

Anwar, F., Latif, S., Ashraf, M., Gilani, A.H., 2007. Moringa oleifera: A Food Plant with Multiple Medicinal Uses. Phytother Res 21, 17-25. DOI: 10.1002/ptr.2023.

Asiedu-Gyekye, I.J., Frimpong-Manso, S., Awortwe, C., Antwi, D.A., Nyarko, A.K., 2014. Micro- and Macroelemental Composition and Safety Evaluation of the Nutraceutical Moringa oleifera Leaves. J Toxicol. 2014, 1-13. <http://dx.doi.org/10.1155/2014/786979>.

Azabji-Kenfack, M., Dikosso, S.E., Loni, E.G., Onana, E.A., Sobngwi, E., Gbaguidi, E., Kana, A.L.N., Nguefack-Tsague, G., der Weid, D.V., Njoya, O., Ngogang, J., 2011. Potential of Spirulina Platensis as a Nutritional Supplement in Malnourished HIV-Infected Adults in Sub-Saharan Africa: A Randomised, Single-Blind Study. Nutr Metabol Insights 4, 29-37.

Biswas, M.H.A., 2012. AIDS epidemic worldwide and the millennium development strategies: A light for lives. HIV & AIDS Rev. 11, 87-94.

Black, R.E., Victora, C.G., Walker, S.P., Bhutta, Z.A., Christian, P., Onis, M., Ezzati, M., Grantham-McGregor, S., Katz, J., Martorell, R., Uauy, R., 2013. Maternal and child undernutrition and overweight in low-income and middle-income countries. Lancet 382, 427-451. <http://dx.doi.org/410.1016/S0140-6736(1013)60937-X>.

Blaise Savadogo, L.G., Donnen, P., Kouéta, F., Kafando, E., Hennart, P., Dramaix, M., 2013. Impact of multivitamin and mineral supplements on mortality and nutritional status of hospitalized severely malnourished HIV/AIDS infected children. Open J Epidemiol 3, 213-219. <http://dx.doi.org/210.4236/ojepi.2013.34031>.

Bunupuradah, T., Ubolyam, S., Hansudewechakul, R., Kosalaraksa, P., Ngampiyaskul, C., Kanjanavanit, S., Wongsawat, J., Luesomboon, W., Pinyakorn, S., Kerr, S., Ananworanich, J., Chomtho, S., van der Lugt, J., Luplertlop, N., Ruxrungtham, K., Puthanakit, T., 2012. Correlation of Selenium and Zinc Levels to Antiretroviral Treatment Outcomes in Thai HIV-infected Children without Severe HIV Symptoms. Eur J Clin Nutr 66, 900-905. DOI:910.1038/ejcn.2012.1057.

Cohen, J., 1988. Statistical power analysis for the behavioral sciences (2nd ed.). Hillsdale, N J: Lawrence Earlbaum Associates.

Contri, P.V., Berchielli, E.M., Tremeschin, M.H., Negrini, B.V., Saloma˜o, R.G., Monteiro, J.P., 2011. Nutritional status and lipid profile of HIV-positive children and adolescents using antiretroviral therapy. Clinics 66, 997-1002. DOI:1010.1590/S1807-59322011000600013.

Cosgrove, S.D., Black, K.E., 2013. Sodium supplementation has no effect on endurance performance during a cycling time-trial in cool conditions: a randomised cross-over trial. J. Int. Soc. Sports Nutr. 10, 1-7.

da Silva, J., Bunn, K., Bertoni, R.F., Neves, O.A., Traebert, J., 2013. Quality of life of people living with HIV. AIDS Care 25, 71-76.

Dibari, F., Bahwere, P., Huerga, H., Irena, A.H., Owino, V., Collins, S., Seal, A., 2013. Development of a cross-over randomized trial method to determine the acceptability and safety of novel ready-to-use therapeutic foods. Nutrition 29, 107-112. DOI:110.1016/j.nut.2012.1004.1016.

Eluwa, G.I., Badru, T., Akpoigbe, K.J., 2012. Adverse drug reactions to antiretroviral therapy (ARVs): incidence, type and risk factors in Nigeria. BMC Clin Pharmacol 12, 1-9.

Fahey, J.W., 2005. Moringa oleifera: A Review of the Medical Evidence for Its Nutritional,Therapeutic, and Prophylactic Properties. Trees for Life Journal 1, 1-24. <http://www.tfljournal.org/article.php/20051201124931586>.

FAO/Nutrition and Consumer Protection Division., v.o.M., 2007., version of May, 2007. Dietary diversity questionnaire.

Farinola, L.A., Famuyide, O.O., Awe, F., Adio, A.F., Ewolor, A.S., 2014. Households’ perception, awareness and willingness to pay for Moringa oleifera Lam powder in Oyo State. J Agric Crop Res 2, 94-103.

Faul, F., Erdfelder, E., Buchner, A., Lang, A.-G., 2009. Statistical power analyses using G*Power 3.1: Tests for correlation and regression analyses. . Behav 41, 1149-1160. DOI: 1110.3758/BRM.1141.1144.1149.

Fawzi, W.W., Msamanga, G.I., Spiegelman, D., Wei, R., Kapiga, S., Villamor, E., Mwakagile, D., Mugusi, F., Hertzmark, E., Essex, M., Hunter, D.J., 2004. A Randomized Trial of Multivitamin Supplements and HIV Disease Progression and Mortality. N Engl J Med 351, 23-32.

Flax, V.L., Phuka, J., Cheung, Y.B., Ashorn, U., Maleta, K., Ashorn, P., 2010. Feeding patterns and behaviors during home supplementation of underweight Malawian children with lipid-based nutrient supplements or corn-soy blend. Appetite 54, 504-511.

Frank, L.B., Schall, J.I., Samuel, J., Zemel, B.S., Dougherty, K.A., Tuluc, F., Rutstein, R.M., Stallings, V.A., 2014. Dietary and Supplement Intake of HIV Infected Children and Young Adults. ICAN: Infant, Child Adolescent Nutrition 6, 221-232.

Hsiung, P., Fang, C., Wu, C., Sheng, W., Chen, S., Wang, J., Yao, G., 2011. Validation of the WHOQOL-HIV BREF among HIVinfected patients in Taiwan. AIDS Care 23, 1035-1042.

Idohou-Dossou, N., Diouf, A., Gueye, A.L., Guiro, A.T., Wade, S., 2011. Impact of daily consumption of Moringa (Moringa Oleifera) dry leaf powder on iron status of senegalese lactating women. AJFAND 11 4985-4999.

Jochum, F., Fuchs, A., Cser, A., Menzel, H., Lombeck, I., 1995. Trace Mineral Status of Full-term Infants Fed Human Milk, Milk-based Formula or Partially Hydrolysed Whey Protein Formula. Analyst, 120, 905-909.

Kimani-Murage, E.W., Norris, S.A., Pettifor, J.M., Tollman, S.M., Klipstein-Grobusch, K., Gómez-Olivé, X.F., Dunger, D.B., Kahn, K., 2011. Nutritional status and HIV in rural South African children. BMC Pediatr 11, 1-13. <http://www.biomedcentral.com/1471-2431/1411/1423>.

Krugera, H.S., Balk, L.J., Viljoen, M., Meyers, T.M., 2013. Positive association between dietary iron intake and iron status in HIV-infected children in Johannesburg, South Africa. Nutri Res 33, 50-58.

La-Bio Research cc., 2015. Repeated Dose 90-day Oral Toxicity Study of Moringa oleifera Dried Leaf Powder. La-Bio Research cc, Eland Technopark 33 Eland Street Pretoria South Africa STUDY NO: IM-0003-2013, 1-32.

Matilsky, D.K., Maleta, K., Castleman, T., Manary, M.J., 2009. Supplementary Feeding with Fortified Spreads Results in Higher Recovery Rates Than with a Corn/Soy Blend in Moderately Wasted Children. J. Nutr. 139, 773-778.

Max, B., Sherer, R., 2000. Management of the Adverse Effects of Antiretroviral Therapy and Medication Adherence. Clin Infect Dis 30, 96–116.

McHenry, M.S., Dixit, A., Vreeman, R.C., 2014. A Systematic Review of Nutritional Supplementation in HIV-Infected Children in Resource-Limited Settings. JIAPAC, 1-11. DOI: 10.1177/2325957414539044.

Mda, S., van Raaij, J.M.A., de Villiers, F.P.R., MacIntyre, U.E., Kok, F.J., 2010. Short-Term Micronutrient Supplementation Reduces the Duration of Pneumonia and Diarrheal Episodes in HIV-Infected Children. J. Nutr. 140, 969-974.

Modi, D.C., Patel, J.K., Shah, B.N., Nayak, B.S., 2010. Phytopharmacology of Moringa Oleifera – an edible plant. Pharmacologyonline 2, 692-705.

Monera, T.G., Maponga, C.C., 2012. Prevalence and patterns of Moringa oleifera use among HIV positive patients in Zimbabwe: a cross-sectional survey. JPHiA 3:e6, 22-24. DOI:10.4081/jphia.2012.e4086.

Monera, T.G., Wolfe, A.R., Maponga, C.C., Benet, L.Z., Guglielmo, J., 2008. Moringa oleifera leaf extracts inhibit 6β-hydroxylation of testosterone by CYP3A4. J Infect Dev Ctries 2, 379-383. DOI:310.3855/jidc.3201.

Ndeezi, G., Tylleskär, T., Ndugwa, C.M., Tumwine, J.K., 2010. Effect of multiple micronutrient supplementation on survival of HIV-infected children in Uganda: a randomized, controlled trial. JIAS 13, 1-9. <http://www.jiasociety.org/content/13/11/18>.

Ndekha, M.J., Manary, M.J., Ashorn, P., Briend, A., 2005. Home-based therapy with ready-to-use therapeutic food is of benefit to malnourished, HIV-infected Malawian children. Acta Pædiatr 94, 222-225.

Nigeria GARPR, 2014. Global AIDS Response Progress Report.

Nunnari, G., Coco, C., Pinzone, M.R., Pavone, P., Berretta, M., Di Rosa, M., Schnell, M., Calabrese, G., Cacopardo, B., 2012. The role of micronutrients in the diet of HIV-1-infected individuals. Front (Elite edition) 4, 2442-2456.

Oduro, I., Ellis, W.O., Owusu, D., 2008. Nutritional potential of two leafy vegetables: Moringa oleifera and Ipomoea batatas leaves. Scientific Res Essay 3, 57-60.

Olisah, V.O., Baiyewu, O., Sheikh, T.L., 2011. Depression underdiagnosis and the effects on quality of life in outpatients with HIV at a Nigerian university teaching hospital. AJAR 10, 247-254. DOI: 210.2989/16085906.16082011.16626294.

Osewa, S.O., Adeniran, A.A., Alamu, O., Olubiyi, M.R., Adeloju, A., 2013. Perception of Rural Dwellers on the Nutritional and Medicinal Values of Moringa oleifera in Ido Local Government of Oyo State. Greener J Agricul Scien 3 829-835.

Peltzer, K., Preez, N.F., Ramlagan, S., Anderson, J., 2010. Antiretroviral treatment adherence among HIV patients in KwaZulu-Natal, South Africa. BMC Public Health 10, 1-10. <http://www.biomedcentral.com/1471-2458/1410/1111>.

Rajanandh, M.G., Satishkumar, M.N., Elango, K., Suresh, B., 2012. Moringa oleifera Lam. A herbal medicine for hyperlipidemia: A preclinical report. Asian Pac J Trop Dis. , S790-S795.

Reddy, S.P., Resnicow, K., James, S., Kambaran, N., Omardien, R., MBewu, A.D., 2008. Underweight, overweight and obesity among South African adolescents: results of the 2002 National Youth Risk Behaviour Survey. Public Health Nutr 12, 203-207.

Rotimi, C., Okosun, I., Johnson, L., Owoaje, E., Lawoyin, T., Asuzu, M., Kaufman, J., Adeyemo, A., Cooper, R., 1999. The distribution and mortality impact of chronic energy deficiency among adult Nigerian men and women. Eur J Clin Nutr 53, 734-739.

Ruel, M.T., 2003. Operationalizing Dietary Diversity: A Review of Measurement Issues and Research Priorities. J. Nutr. 133, 3911S-3926S.

Seok-Jae, K., Gajin, H., Seul-Ki, K., Jae-Gu, S., Won-Seok, C., Bongha, R., Jinsung, K., Inkwon, Y., Beom-Joon, L., Jin-Moo, L., Jae-Woo, P., 2013. Effect of Korean Herbal Medicine Combined with a Probiotic Mixture on Diarrhea-Dominant Irritable Bowel Syndrome: A Double-Blind, Randomized, Placebo-Controlled Trial. Evidence-Based Complement. Altern. Med. 2013, 1-10.

Steenkamp, L., Dannhauser, A., Walsh, D., Joubert, G., Veldman, F.J., Van der Walt, E., Cox, C., Hendricks, M.K., Dippenaar, H., 2009. Nutritional, immune, micronutrient and health status of HIV-infected children in care centres in Mangaung. S Afr J Clin Nutr 22, 131-136.

Sudawa, A., Ahmad, A.A., Adeleke, S., Umar, L., Rogo, L.D., 2013. HIV Infection among Under-Five Malnourished Children in Kano State. WJA 3, 350-356.

Sunguya, B.F., Poudel, K.C., Mlunde, L.B., Otsuka, K., Yasuoka, J., Urassa, D.P., Mkopi, N.P., Jimba, M., 2012. Ready to Use Therapeutic Foods (RUTF) improves undernutrition among ART-treated, HIV-positive children in Dar es Salaam, Tanzania. Nutr 11, 1-8. <http://www.nutritionj.com/content/11/11/60>.

Swaminathan, S., Padmapriyadarsini, C., Yoojin, L., Sukumar, B., Iliayas, S., Karthipriya, J., Sakthivel, R., Gomathy, P., Thomas, B.E., Mathew, M., Wanke, C.A., Narayanan, P.R., 2010. Nutritional Supplementation in HIV-Infected Individuals in South India: A Prospective Interventional Study. Clin Infect Dis 51, 51-57. DOI: 10.1086/653111.

Sztam, K.A., Fawzi, W.W., Duggan, C., 2010. Macronutrient Supplementation and Food Prices in HIV Treatment. J. Nutr. 140, 213S-223S.

Sztam, K.A., Ndirangu, M., Sheriff, M., Arpadi, S.M., Hawken, M., Rashid, J., Deckelbaum, R.J., El Sadr, W.M., 2013. Rationale and design of a study using a standardized locally procured macronutrient supplement as adjunctive therapy to HIV treatment in Kenya. AIDS Care 25, 1138-1144. DOI: 1110.1080/09540121.09542012.09752564.

Tathiah, N., Moodley, I., Mubaiwa, V., Denny, L., Taylor, M., 2013. South Africa’s nutritional transition: Overweight, obesity, underweight and stunting in female primary school learners in rural KwaZulu-Natal, South Africa. S Afr Med J 103, 718-723.

Tété–Bénissan, A., Quashie, M.I.A., Lawson–Evi, K., Gnandi, K., Kokou, K., Gbéassor, M., 2013. Influence of Moringa oleifera leaves on atherogenic lipids and glycaemia evolution in HIV-infected and uninfected malnourished patients. J Appl Biosci 62, 4610-4619. DOI: 4610.4314/jab.v4662i4610.86072.

Thompson, F.E., Subar, A.F., 2001. Dietary Assessment Methodology. Nutr Prev Treatm Diseas, 2nd ed, 1-38.

Thurber, M.D., Fahey, J.W., 2009. Adoption of Moringa oleifera to combat under-nutrition viewed through the lens of the “Diffusion of Innovations” theory. Ecol Food Nutr 48, 212-225. DOI: 210.1080/03670240902794598.

Tofail, F., Persson, L.Å., El Arifeen, S., Hamadani, J.D., Mehrin, F., Ridout, D., Ekström, E., Huda, S.N., Grantham-McGregor, S.M., 2008. Effects of prenatal food and micronutrient supplementation on infant development: a randomized trial from the Maternal and Infant Nutrition Interventions, Matlab (MINIMat) study. Am J Clin Nutr 87, 704-711.

UNAIDS, 2013. UNAIDS report on the global AIDS epidemic.

UNAIDS, 2015. UNAIDS report on the global AIDS epidemic.

UNAIDS Global Report., 2013. UNAIDS Global Report.

UNAIDS/AVAC, 2011. Good participatory practice Guidelines for biomedical HIV prevention trials 2011. UNAIDS, 1-88.

UNICEF., 2013. Ready-to-Use Therapeutic Food: Current Outlook. UNICEF Supply Division.

Weiser, S.D., Young, S.L., Cohen, C.R., B Kushel, M.B., Tsai, A.C., Tien, P.C., Hatcher, A.M., Frongillo, E.A., Bangsberg, D.R., 2011. Conceptual framework for understanding the bidirectional links between food insecurity and HIV/AIDS. Am J Clin Nutr 94 (suppl), 1729S-1739S.

WHO., 2002. WHOQOL-HIV BREF.

WHO., 2008. Essential Prevention and care interventions for adults and adolescents living with HIV in resource-limited settings WHO.

Wiles, N.L., Mahlangu, Z.N., Siwela, M., Veldman, F.J., 2014. Nutritional quality of a ready-to-use food, and its acceptability to healthy and HIV-infected children receiving antiretroviral treatment. S Afr J Clin Nutr 27, 222-227.

Williams, F.E., Animashaun, J.O., Ibrahim, H., Toye, A.A., 2013. A preliminary survey on consumption of moringa products for nutraceutical benefits in ilorin, kwara state, nigeria. Agrosearch 13, 165-175. DOI: 110.4314/agrosh.v4313i4311.4315.

**APPENDIX 1: PATIENT INFORMED CONSENT FORM**

**UKZN BIOMEDICAL RESEARCH ETHICS COMMITTEE**

## Information Sheet and Consent to Participate in Research

Date:

Dear Sir/ Madam,

My name is Aisha Gambo, a Ph.D student in the Discipline of Public Health Medicine, University of Kwazulu-Natal, Durban, South Africa. My contact number is +234035392408 and my email address is [gamboaishatu@yahoo.com](mailto:gamboaishatu@yahoo.com)

You are being invited to consider participating in a study titled

**“Evaluation of the addition of Moringa oleifera as a nutritional supplement on the anthropometric, viral load and CD4 counts of patients on antiretroviral therapy”**

The purpose of this research is to find out if Moringa oleifera as a dietary supplement will have any effect on the body measurements (weight and BMI) and HIV status (CD4 and viral load) of people living with HIV and AIDS and on anti-retroviral therapy (ART). The viral load of the participants will be monitored only if and when it is done in the clinic.

The study is important because we know that people living with HIV and AIDS have better health outcomes if they have good nutrition. We also know that the leaves of Moringa oleifera is packed with nutrients that includes all amino acids (proteins), many vitamins, minerals and anti-oxidants. All of these nutrients are essential for daily functioning. We need to evaluate and determine if adding Moringa oleifera supplement to your normal meals will make a difference to you in terms of your weight and also your immune status (i.e. CD4 counts and viral loads). To do this in a scientific manner, we will put people taking part in this study into two groups. These groups are selected purely by chance to eliminate any bias. One group will be given Moringa oleifera and the other group, the control will be given cornstarch which will be similar in look and presentation (placebo). If you choose to participate in this study, you should not have any known allergy or intolerance to Moringa oleifera or cornstarch. You should be on Tenofovir + Lamivudine + Efavirenz drug combination. You should not be pregnant. Your CD4 counts should be below 500 cells/mm^3^. Also patients with active opportunistic infection and also those taking micronutrient or natural health product supplements within 30days of randomization cannot participate in the study. The study is expected to have a total number of 200 people.

Participants will be given Moringa oleifera or the placebo supplement to take together with their meals three times a day. All participants will be observed for the same measures of weight and immune status. The research team will be looking after all participants closely during the study.

During the research you are expected to make seven visits to the clinic.

- In the first visit, we will ask you a few questions about yourself, your general health and types of food eaten. We will measure your height and weight. Then a small amount of blood, equal to about a teaspoon, will be taken from your arm with a syringe. This blood will be tested for the number of cells that help your body to fight the HIV virus infections (CD4 cells). If and when the test that tells the amount of the HIV virus in your body (viral load) is done in the clinic it will also be conducted.
- At the next visit, which will be two weeks later, you will again be asked some questions about your health and then you will be divided into two groups and be given either the Moringa oleifera supplement or placebo with your usual prescription of ART drugs.
- At all subsequent visits to the clinic thereafter your height, weight and other body measurements will be taken. A blood sample will be taken as explained earlier and the participants will be examined by the clinician if you have any infections associated with HIV and AIDS and how serious the infection is.

The duration of your participation if you choose to enroll and remain in the study is expected to be six months. During this period if you develop injury or become sick as a result of taking Moringa oleifera or the placebo, you will be medically treated free of charge.

The safety of Moringa oleifera has been evaluated and to our knowledge there are no notable toxic effects. Patients on anti-coagulants and pregnant women will be excluded as a precautionary measure.

If you participate in the study, you will benefit from free CD4 and viral load tests (if it is done in the clinic). We hope that the study will enhance your knowledge about the benefits of good nutrition and also about the nutrients benefits of Moringa oleifera and whether it could have any additional benefits to improve the health status of people living with HIV and AIDS. Also society will benefit from the study with the scientific information on the use of Moringa oleifera as nutritional supplements in HIV infection.

This study has been ethically reviewed and approved by the UKZN Biomedical research Ethics Committee (approval number…….) and AKTH Ethics Committee. In the event of any problem or concerns/questions you may contact the researcher at +234035392408 or the research team at HIV clinic, AKTH

Please note that your participation in this study is entirely voluntary. It is your choice whether to participate or not. Whether you participate or not, all the services you receive at this clinic will continue and nothing will change. If you agreed earlier and you later change your mind to stop participating in the study, you will need to inform the researcher on this number +234035392408 or inform the research team at HIV clinic, AKTH. Also if you chose to participate, the researcher can terminate you from participating in the study if you refuse to adhere to the basic protocols of the study.

We will give you [R 15] to pay for your travel to the clinic for the purpose of the study on days other than your normal clinic days. You will not be given any other money or gifts to take part in this study.

With this study, it is possible if people are aware that you are participating, they may ask you questions. We will not be sharing the identity of those participating in the study. The information that we collect from this research project will be kept confidential. Information about you that will be collected during the research will be put away and no-one but the researchers will be able to see it. Any information about you will have a number on it instead of your name. Only the researchers will know what your number is and we will lock that information up under lock and key. It will not be shared with or given to anyone except the researcher, the AKTH HIV management team and your doctors.

At the end of the study, in six months, any leftover blood samples taken during the course of the study will be destroyed. The result of the study will be made known to you at the clinic and thereafter it will be published in local and international journals without disclosing your identity.

---------------------------------------------------------------------------------------------------------------

**CONSENT**

I (Name)……………………have been informed about the study entitled (provide details) ……………………………….. by (provide name of researcher/fieldworker) …………………………………...

I understand the purpose and procedures of the study (add these again if appropriate).

I have been given an opportunity to answer questions about the study and have had answers to my satisfaction.

I declare that my participation in this study is entirely voluntary and that I may withdraw at any time without affecting any treatment or care that I would usually be entitled to.

I have been informed about any available compensation or medical treatment if injury occurs to me as a result of study-related procedures.

If I have any further questions/concerns or queries related to the study I understand that I may contact the researcher at +234035392408

If I have any questions or concerns about my rights as a study participant, or if I am concerned about an aspect of the study or the researchers then I may contact:

BIOMEDICAL RESEARCH ETHICS ADMINISTRATION

Research Office, Westville Campus

Govan Mbeki Building

University of KwaZulu-Natal

Private Bag X 54001, Durban, 4000

KwaZulu-Natal, SOUTH AFRICA

Tel: 27 31 2602486 - Fax: 27 31 2604609

Email: [BREC@ukzn.ac.za](mailto:ngwenyap@ukzn.ac.za)

OR

Adult HIV Clinic,

S. S. Wali virology clinic

Aminu Kano Teaching Hospital kano, Nigeria

Mobile No: 08035392408

**____________________ ____________________**

**Signature of Participant Date**

**____________________ _____________________**

**Signature of Witness Date**

**(Where applicable)**

**____________________ _____________________**

**Signature of Translator Date**

**(Where applicable)**

**APPENDIX 2 : FIRST VISIT DATA COLLECTION SHEET**

**FIRST VISIT DATA COLLECTION SHEET**

**Section 1**: **Socio-demographic information of participant**

Before you begin we would like to ask you to answer a few general questions about yourself by ticking the correct answer.

Gender

Male □

Female □

Age

18 – 25 years □

26 – 35 years □

36 – 45 years □

46 – 55 years □

Above 60 years □

Religion

Islam □

Christianity □

Others □

Ethnicity

Hausa / Fulani □

Yoruba □

Igbo □

Others □

Marital status

Single □

Married □

Divorced □

Widowed □

Education

Primary □

Secondary □

Tertiary □

Quranic □

None □

Occupation

Farmer □

Trader □

Civil servant □

Artisan □

Unemployed □

Others □

Members in your family

2 – 5 □

6 – 10 □

Above 10 □

Monthly income (R)

Less than 1000 □

1001 – 5000 □

5001 – 10000 □

10001 – 30000 □

30001 – 50000 □

Above 50000 □

**Section 2**: **Diet History of participant**

This section is going to ask you what you eat and how often you eat it monthly.

You are going to write down your answer in the space provided.

What do you often eat for breakfast?

|  |
| --- |

How many times do you eat it in a month?

|  |
| --- |

What do you often eat for lunch?

|  |
| --- |

How many times do you eat it in a month?

|  |
| --- |

What do you often eat for supper?

|  |
| --- |

How many times do you eat it in a month?

|  |
| --- |

**APPENDIX 3: SUBSEQUENT VISIT DATA COLLECTION SHEET**

**SUBSEQUENT VISIT DATA COLLECTION SHEET**

**Diet History of participant**

This section is going to ask you what you eat and how often you eat it monthly.

You are going to write down your answer in the space provided.

What do you often eat for breakfast?

|  |
| --- |

How many times do you eat it in a month?

|  |
| --- |

What do you often eat for lunch?

|  |
| --- |

How many times do you eat it in a month?

|  |
| --- |

What do you often eat for supper?

|  |
| --- |

How many times do you eat it in a month?

|  |
| --- |

**APPENDIX 4:**

***DIETARY DIVERSITY QUESTIONNAIRE ^1^***

**Please describe the foods (meals and snacks) that you ate yesterday during the day and night, whether at home or outside the home. Start with the first food eaten in the morning.**

*Write down all food and drinks mentioned by the respondent. When the respondent has finished, probe for meals and snacks not mentioned.*

| **Breakfast** | **Snack** | **Lunch** | **Snack** | **Dinner** | **Snack** |
| --- | --- | --- | --- | --- | --- |
|  |  |  |  |  |  |

***[Household level: consider foods eaten by any member of the household, and exclude foods purchased and eaten outside of the home]***

*When the respondent recall is complete, fill in the food groups based on the information recorded above. For any food groups not mentioned, ask the respondent if a food item from this group was consumed.*

| Question | Food group | Examples | YES=1 |  |
| --- | --- | --- | --- | --- |
| number |  |  | NO=0 |  |
|  |  |  |  |  |
|  |  |  |  |  |
| 1 | CEREALS | corn/maize, rice, wheat, sorghum, millet or any other |  |  |
|  |  | grains or foods made from these (e.g. bread, noodles, |  |  |
|  |  | porridge or other grain products) + *insert local foods e.g.* |  |  |
|  |  | *ugali, nshima, porridge or pastes or other locally* |  |  |
|  |  | *available grains* |  |  |
|  |  |  |  |  |
| 2 | VITAMIN A RICH | pumpkin, carrots, squash, or sweet potatoes that are |  |  |
|  | VEGETABLES AND | orange inside + *other locally available vitamin-A rich* |  |  |
|  | TUBERS | *vegetables (e.g. red sweet pepper)* |  |  |
|  |  |  |  |  |
| 3 | WHITE TUBERS AND | white potatoes, white yams, white cassava, or other |  |  |
|  | ROOTS | foods made from roots |  |  |
|  |  |  |  |  |
| 4 | DARK GREEN LEAFY | dark green/leafy vegetables, including wild ones + *locally* |  |  |
|  | VEGETABLES | *available vitamin-A rich leaves such as amaranth,* |  |  |
|  |  | *cassava leaves, kale, spinach etc.* |  |  |
|  |  |  |  |  |
| 5 | OTHER VEGETABLES | other vegetables (e.g. tomato, onion, eggplant) , |  |  |
|  |  | including wild vegetables |  |  |
|  |  |  |  |  |
| 6 | VITAMIN A RICH | ripe mangoes, cantaloupe, apricots (fresh or dried), ripe |  |  |
|  | FRUITS | papaya, dried peaches + *other locally available vitamin* |  |  |
|  |  | *A-rich fruits* |  |  |
| 7 | OTHER FRUITS | other fruits, including wild fruits |  |  |
|  |  |  |  |  |
| 8 | ORGAN MEAT (IRON- | liver, kidney, heart or other organ meats or blood-based |  |  |
|  | RICH) | foods |  |  |
|  |  |  |  |  |
| 9 | FLESH MEATS | beef, pork, lamb, goat, rabbit, wild game, chicken, duck, |  |  |
|  |  | or other birds |  |  |
|  |  |  |  |  |
| 10 | EGGS | chicken, duck, guinea hen or any other egg |  |  |
|  |  |  |  |  |
| 11 | FISH | fresh or dried fish or shellfish |  |  |
|  |  |  |  |  |

| 12 | |  | LEGUMES, NUTS AND | beans, peas, lentils, nuts, seeds or foods made from |  |  |
| --- | --- | --- | --- | --- | --- | --- |
|  |  |  | SEEDS | these |  |  |
|  |  |  |  |  |  |  |
| 13 | |  | MILK AND MILK | milk, cheese, yogurt or other milk products |  |  |
|  |  |  | PRODUCTS |  |  |  |
|  |  |  |  |  |  |  |
| 14 | |  | OILS AND FATS | oil, fats or butter added to food or used for cooking |  |  |
|  |  |  |  |  |  |  |
| 15 | |  | RED PALM PRODUCTS | Red palm oil, palm nut or palm nut pulp sauce |  |  |
|  |  |  |  |  |  |  |
| 16 | |  | SWEETS | sugar, honey, sweetened soda or sugary foods such as |  |  |
|  |  |  |  | chocolates, candies, cookies and cakes |  |  |
|  |  |  |  |  |  |  |
| 17 | |  | SPICES, CONDIMENTS, | spices(black pepper, salt), condiments (soy sauce, hot |  |  |
|  |  |  | BEVERAGES | sauce), coffee, tea, alcoholic beverages OR *local* |  |  |
|  |  |  |  | *examples* |  |  |
|  |  |  |  |  |  |  |
|  |  |  |  |  | YES=1 |  |
|  |  |  |  |  | NO=0 |  |
|  |  |  |  |  |  |  |
|  | Individual |  | Did you eat anything (meal or snack) OUTSIDE of the home yesterday? | |  |  |
|  | level only |  |  |  |  |  |
|  |  |  |  |  |  |  |
|  |  |  |  | |  |  |
|  | Household |  | Did you or anyone in your household eat anything (meal or snack) OUTSIDE of the | |  |  |
|  |  |  | home yesterday? |  |  |  |
|  | level only |  |  |  |  |  |
|  |  |  |  |  |  |  |
|  |  |  |  |  |  |  |

^1^ FAO/Nutrition and Consumer Protection Division, version of May, 2007. Please acknowledge FAO in any documents pertaining to use of this questionnaire.

^2^ This questionnaire may be used for any individual above the age of three years. For children under three, the dietary diversity questionnaire used in DHS surveys for young children is more appropriate.

**APPENDIX 5: WORLD HEALTH ORGANISATION QUALITY OF LIFE- HIV BREF**

WHO/MSD/MER/02.2

English only

Distr.: General

WHOQOL-HIV BREF

MENTAL HEALTH: EVIDENCE AND RESEARCH DEPARTMENT OF MENTAL HEALTH

AND SUBSTANCE DEPENDENCE

WORLD HEALTH ORGANIZATION

GENEVA

Raw Transformed

Score Score

| Domain 1 | (6-Q3) + (6-Q4) + Q14 + Q21 |  |  |
| --- | --- | --- | --- |
|  |  |  |  |
|  | □ + □ + □ + □ |  |  |
| Domain 2 | Q6 + Q11 + Q15 + Q24 + (6-Q31) |  |  |
|  |  |  |  |
|  | □ + □ + □ + □ + □ |  |  |
| Domain 3 | (6-Q5) + Q20 + Q22 + Q23 |  |  |
|  |  |  |  |
|  | □ + □ + □ + □ |  |  |
| Domain 4 | Q17 + Q25 + Q26 + Q27 |  |  |
|  |  |  |  |
|  | □ + □ + □ + □ |  |  |
| Domain 5 | Q12 + Q13 + Q16 + Q18 + Q19 + Q28 + Q29 + Q30 |  |  |
|  |  |  |  |
|  | □ + □ + □ + □ + □ + □+ □ + □ |  |  |
| Domain 6 | Q7 + (6–Q8) + (6-Q9) + (6-Q10) |  |  |
|  |  |  |  |
|  | □ + □ + □ + □ |  |  |

Further copies of this document may be obtained from

**Department of Mental Health and Substance Dependence**

World Health Organization

CH-1211 Geneva 27

Switzerland

2

**ABOUT YOU**

Before you begin we would like to ask you to answer a few general questions about yourself: by circling the correct answer or by filling in the space provided.

What is your **gender**? Male / Female

How old are you? ______________ (age in years)

What is the highest level **education** you received? None at all / Primary / Secondary / Tertiary / Quranic

What is your **Ethnicity**? Hausa:Fulani / Yoruba / Igbo / Others

What is your **Marital status**? Single / Married / Divorced / Separated / Widowed

What is your **Occupation**? Farmer / Trader / Civil servant / Artisan / Unemployed / Others

How many members are in your family? ________________

What is your **monthly income**? ________________

If there is something wrong with you, what do you think it is?

***Please respond to the following questions if they are applicable to you:***

What is your **HIV serostatus**? Asymptomatic / Symptomatic / AIDS converted

In what year did you first **test positive** for HIV?

In what year do you think you were infected?

How do you believe you were **infected with HIV**? (circle one only):

Sex with a man / Sex with a woman / Injecting drugs / Blood products / Other (specify)_________________

***Instructions***

This assessment asks how you feel about your quality of life, health, or other areas of your life. **Please answer all the** **questions.** If you are unsure about which response to give to a question, **please choose the one** that appears most appropriate. This can often be your first response. Please keep in mind your standards, hopes, pleasures and concerns. We ask that you think about your life **in the last two weeks.** For example, thinking about the last two weeks, a question might ask:

|  |  |  |  |  |  |  |  |  |
| --- | --- | --- | --- | --- | --- | --- | --- | --- |
|  |  |  | Not at all | A little | A moderate | Very much | Extremely |  |
|  |  |  |  |  | amount |  |  |  |
|  |  |  |  |  |  |  |  |  |
|  | 11 (F5.3) | How well are you able to concentrate? | 1 | 2 | 3 | 4 | 5 |  |
|  |  |  |  |  |  |  |  |  |
|  |  |  |  |  |  |  |  |  |

You should circle the number that best fits how well are you able to concentrate over the last two weeks. So you would circle the number 4 if you were able to concentrate very much. You would circle number 1 if you were not able to concentrate at all in the last two weeks.

3

**Please read each question, assess your feelings, and circle the number on the scale for each question that gives the best answer for you.**

|  |  |  |  |  |  |  |  |  |
| --- | --- | --- | --- | --- | --- | --- | --- | --- |
|  |  |  |  | Very poor | Poor | Neither poor | Good | Very good |
|  |  |  |  |  |  | nor good |  |  |
|  |  |  |  |  |  |  |  |  |
|  |  | 1(G1) | How would you rate your quality of life? | 1 | 2 | 3 | 4 | 5 |
|  |  |  |  |  |  |  |  |  |
|  |  |  |  |  |  |  |  |  |
|  |  |  |  |  |  |  |  |  |
|  |  |  |  | Very | Dissatisfied | Neither | Satisfied | Very |
|  |  |  |  | dissatisfied |  | satisfied nor |  | satisfied |
|  |  |  |  |  |  | dissatisfied |  |  |
|  |  |  |  |  |  |  |  |  |
|  |  | 2 (G4) | How satisfied are you with your health? | 1 | 2 | 3 | 4 | 5 |
|  |  |  |  |  |  |  |  |  |
|  |  |  |  |  |  |  |  |  |

The following questions ask about **how much** you have experienced certain things in the last two weeks.

|  |  |  |  |  |  |  |  |  |
| --- | --- | --- | --- | --- | --- | --- | --- | --- |
|  |  |  |  | Not at all | A little | A moderate | Very much | An extreme |
|  |  |  |  |  |  | amount |  | amount |
|  |  |  |  |  |  |  |  |  |
| 3 | (F1.4) | To what extent do you feel that physical pain | 1 | | 2 | 3 | 4 | 5 |
|  |  | prevents you from doing what you need to |  |  |  |  |  |  |
|  |  | do? |  |  |  |  |  |  |
|  |  |  |  |  |  |  |  |  |
| 4 | (F50.1) | How much are you bothered by any physical | 1 | | 2 | 3 | 4 | 5 |
|  |  | problems related to your HIV infection? |  |  |  |  |  |  |
|  |  |  |  |  |  |  |  |  |
| 5 | (F11.3) | How much do you need any medical | 1 | | 2 | 3 | 4 | 5 |
|  |  | treatment to function in your daily life? |  |  |  |  |  |  |
|  |  |  |  |  |  |  |  |  |
| 6 | (F4.1) | How much do you enjoy life? | 1 | | 2 | 3 | 4 | 5 |
|  |  |  |  |  |  |  |  |  |
| 7 | (F24.2) | To what extent do you feel your life to be | 1 | | 2 | 3 | 4 | 5 |
|  |  | meaningful? |  |  |  |  |  |  |
|  |  |  |  |  |  |  |  |  |
| 8 | (F52.2) | To what extent are you bothered by people | 1 | | 2 | 3 | 4 | 5 |
|  |  | blaming you for your HIV status |  |  |  |  |  |  |
|  |  |  |  |  |  |  |  |  |
| 9 | (F53.4) | How much do you fear the future? | 1 | | 2 | 3 | 4 | 5 |
|  | |  |  |  |  |  |  |  |
| 10 (F54.1) | | How much do you worry about death? | 1 | | 2 | 3 | 4 | 5 |
|  |  |  |  |  |  |  |  |  |
|  |  |  |  |  |  |  |  |  |
|  |  |  |  |  |  |  |  |  |
|  |  |  |  | Not at all | A little | A moderate | Very much | Extremely |
|  |  |  |  |  |  | amount |  |  |
|  | |  |  |  |  |  |  |  |
| 11 (F5.3) | | How well are you able to concentrate? |  | 1 | 2 | 3 | 4 | 5 |
|  | |  |  |  |  |  |  |  |
| 12 (F16.1) | | How safe do you feel in your daily life? |  | 1 | 2 | 3 | 4 | 5 |
|  | |  |  |  |  |  |  |  |
| 13 (F22.1) | | How healthy is your physical environment? |  | 1 | 2 | 3 | 4 | 5 |
|  |  |  |  |  |  |  |  |  |
|  |  |  |  |  |  |  |  |  |

The following questions ask about **how completely** you experience or were able to do certain things in the last two weeks.

|  |  |  |  |  |  |  |  |
| --- | --- | --- | --- | --- | --- | --- | --- |
|  |  |  | Not at all | A little | Moderately | Mostly | Completely |
|  |  |  |  |  |  |  |  |
| 14 | (F2.1) | Do you have enough energy for everyday | 1 | 2 | 3 | 4 | 5 |
|  |  | life? |  |  |  |  |  |
|  |  |  |  |  |  |  |  |
| 15 | (F7.1) | Are you able to accept your bodily | 1 | 2 | 3 | 4 | 5 |
|  |  | appearance? |  |  |  |  |  |
|  |  |  |  |  |  |  |  |
| 16 | (F18.1) | Have you enough money to meet your needs? | 1 | 2 | 3 | 4 | 5 |
|  |  |  |  |  |  |  |  |
| 17 | (F51.1) | To what extent do you feel accepted by the | 1 | 2 | 3 | 4 | 5 |
|  |  | people you know? |  |  |  |  |  |
|  |  |  |  |  |  |  |  |
| 18 | (F20.1) | How available to you is the information that | 1 | 2 | 3 | 4 | 5 |
|  |  | you need in your day-to-day life? |  |  |  |  |  |
|  |  |  |  |  |  |  |  |
|  |  |  |  |  |  |  |  |
|  |  |  |  |  |  |  |  |

4

|  |  | 19 (F21.1) | | To what extent do you have the opportunity | | 1 | 2 | 3 | 4 | 5 |
| --- | --- | --- | --- | --- | --- | --- | --- | --- | --- | --- |
|  |  |  |  | for leisure activities? | |  |  |  |  |  |
|  |  |  |  |  |  |  |  |  |  |  |
|  |  |  |  |  |  |  |  |  |  |  |
|  |  |  |  |  |  |  |  |  |  |  |
|  |  |  |  |  |  | Very poor | Poor | Neither poor | Good | Very good |
|  |  |  |  |  |  |  |  | nor good |  |  |
|  |  |  |  |  |  |  |  |  |  |  |
|  | 20 | | (F9.1) |  | How well are you able to get around? | 1 | 2 | 3 | 4 | 5 |
|  |  |  | | |  |  |  |  |  |  |
|  |  |  |  |  |  |  |  |  |  |  |
|  |  | The following questions ask you how **good or satisfied** you have felt about various aspects of your life over the last two | | | | | | | | |
|  |  | weeks. | |  |  |  |  |  |  |  |
|  |  |  |  |  |  |  |  |  |  |  |
|  |  |  |  |  |  | Very | Dissatisfied | Neither | Satisfied | Very |
|  |  |  |  |  |  | dissatisfied |  | satisfied nor |  | satisfied |
|  |  |  |  |  |  |  |  | dissatisfied |  |  |
|  |  |  |  |  |  |  |  |  |  |  |
|  |  | 21 | (F3.3) |  | How satisfied are you with your sleep? | 1 | 2 | 3 | 4 | 5 |
|  |  |  |  |  |  |  |  |  |  |  |
|  |  | 22 | (F10.3) |  | How satisfied are you with your ability to | 1 | 2 | 3 | 4 | 5 |
|  |  |  |  |  | perform your daily living activities? |  |  |  |  |  |
|  |  |  |  |  |  |  |  |  |  |  |
|  |  | 23 | (F12.4) |  | How satisfied are you with your capacity for | 1 | 2 | 3 | 4 | 5 |
|  |  |  |  |  | work? |  |  |  |  |  |
|  |  |  |  |  |  |  |  |  |  |  |
|  |  | 24 | (F6.3) |  | How satisfied are you with yourself? | 1 | 2 | 3 | 4 | 5 |
|  |  |  |  |  |  |  |  |  |  |  |
|  |  | 25 | (F13.3) |  | How satisfied are you with your personal | 1 | 2 | 3 | 4 | 5 |
|  |  |  |  |  | relationships? |  |  |  |  |  |
|  |  |  |  |  |  |  |  |  |  |  |
|  |  | 26 | (F15.3) |  | How satisfied are you with your sex life? | 1 | 2 | 3 | 4 | 5 |
|  |  |  |  |  |  |  |  |  |  |  |
|  |  | 27 | (F14.4) |  | How satisfied are you with the support you | 1 | 2 | 3 | 4 | 5 |
|  |  |  |  |  | get from your friends? |  |  |  |  |  |
|  |  |  |  |  |  |  |  |  |  |  |
|  |  | 28 | (F17.3) |  | How satisfied are you with the conditions of | 1 | 2 | 3 | 4 | 5 |
|  |  |  |  |  | your living place? |  |  |  |  |  |
|  |  |  |  |  |  |  |  |  |  |  |
|  |  | 29 | (F19.3) |  | How satisfied are you with your access to | 1 | 2 | 3 | 4 | 5 |
|  |  |  |  |  | health services? |  |  |  |  |  |
|  |  |  |  |  |  |  |  |  |  |  |
|  |  | 30 | (F23.3) |  | How satisfied are you with your transport? | 1 | 2 | 3 | 4 | 5 |
|  |  |  | | |  |  |  |  |  |  |
|  |  |  |  |  |  |  |  |  |  |  |
|  |  | The following question refers to **how often** you have felt or experienced certain things in the last two weeks. | | | | | | | |  |
|  |  |  |  |  |  |  |  |  |  |  |
|  |  |  |  |  |  | Never | Seldom | Quite often | Very often | Always |
|  |  |  | |  |  |  |  |  |  |  |
|  |  | 31 (F8.1) | |  | How often do you have negative feelings | 1 | 2 | 3 | 4 | 5 |
|  |  |  |  |  | such as blue mood, despair, anxiety, |  |  |  |  |  |
|  |  |  |  |  | depression? |  |  |  |  |  |
|  |  |  |  |  |  |  |  |  |  |  |
|  |  |  |  |  |  |  |  |  |  |  |

Did someone help you to fill out this form?

How long did it take to fill this form out?

Do you have any comments about the assessment?

**THANK YOU FOR YOUR HELP**
