# Supplementary material for: A double-blind, randomized controlled trial to examine the effect of Moringa oleifera leaf powder supplementation on the immune status and anthropometric parameters of adult HIV patients on antiretroviral therapy in a resource-limited setting
Source: PLoS One. 2021 Dec 31;16(12):e0261935. doi: 10.1371/journal.pone.0261935 (PMC8722362; doi:10.1371/journal.pone.0261935)
Supplement: Attachment — Submitted filename: Answers to Reviewers comments_ PLOS ONE.docx [file pone.0261935.s006.docx]

Reviewer #1: *** General comments: ***

The manuscript presents interesting data from what appears to have been a well-run study.

However, the statistical analysis is quite idiosyncratic and fails to control for multiple comparisons. It should be redone.

In addition, the manuscript suffers from a variety of English syntactic and usage errors. These should be corrected. There are relatively few in the results section, they are more frequent in the discussion section, and they are numerous in the remaining sections. There are too many to list.

*** Specific comments: ***

1) The protocol is quite weak in its description of the statistical methods to be used for the data, as well as the hypotheses of interest and the specific tests or contrasts that were to be employed. This weakness appears to have carried over to the actual statistical analysis. The study design is fairly straightforward, so it is unclear why a better framework for evaluation could not be specified.

Having said that, the most important test specified in the protocol is the t-test of the two treatments at the last time point, so this should be presented in any case.

**Reviewer #1**

1) The current layout of the statistical analysis is quite idiosyncratic and simplistic. The following approach or equivalent should be used instead:

Test baseline differences via the t-test as already performed. This is analogous to a two-factor analysis of variance (ANOVA) with one fixed effect --- that is, treatment.

Assess the differences between groups in changes over time by using a linear mixed effect model framework. The fixed effects are treatment, time, and the treatment by time interaction. Include a correlation structure to account for the dependence structure caused by observing the same patient over time. Do not use a compound symmetry structure (equivalent to "classical" repeated measures) --- this is an unrealistic assumption here. Instead, either use an autoregressive correlation structure such as AR(1) (since time points are equally spaced) or use a completely unstructured correlation matrix.

The test of the interaction of treatment by time provides the answer to the question of whether the response profile over time differs between treatments. If there is no interaction effect, that is not necessarily the end of the road, but it is arguably of primary interest.

Next, perform post hoc comparisons using an adjustment for multiple comparisons such as the Tukey-Kramer adjustment. It is up to the authors, they can perform an analysis of either simple effects (comparison between treatments at each time point or comparison among time points within each treatment) or an analysis of all pairwise comparisons. Note that the test of the interaction effect is not technically required to be statistically significant for these tests to be performed, though this is the usual procedure.

All linear models should be assessed for fit using graphical analysis of the residuals to check for symmetry, homoscedasticity, and independence. The Shapiro-Wilk or Kolmogorov-Smirnov test can be used, but is probably of less importance.

If transformations are required, please report all transformations attempted. Even if fit is marginal on the original scale, if the results are qualitatively similar to a fit on a transformed scale then it may be best to report analysis on the original scale for interpretation.

For completeness's sake, since the protocol specifies it, also include the t-test of the last endpoints. However, this is probably less powerful than the result that will be obtained from the linear mixed effects model approach.

2) The evaluation of socio-demographic characteristics (Line 270 forward) should be redone. This should be done in the framework described above, as an exploratory analysis. These covariates can be entered as main effects in linear mixed effects model described above. This will yield answers to the question of whether the treatment effect remains if the effect of the covariate is statistically corrected, which is probably the question that the authors have in mind.

**Response of Reviewer One comments on statistical analysis**

- Linear mixed effect model has been constructed to determine difference in treatment protocol between the two groups. Some interactions observed and other findings from the model have been added to the write-up (Pages 13-14; Lines 261 – 279). [Details included as supplementary information S1]

- The Posthoc test was conducted using Tukey-Kramer adjustment and findings from the Posthoc were similar to the repeated Anova test. [Details included as supplementary information S2]

- The repeated dependent variables such as CD4 counts were restructured into vertical cases to prepare the data for linear mixed model analysis

- The socio-demographic characteristics were included in the mixed model as fixed variables. The effect of the socio-demographic characteristics were identified and discussed. Findings have been included in the manuscript (Pages 14-15; Lines 280 – 293). [Details included as supplementary information S1]

4) In some cases, it is difficult to understand what exactly is being presented. For example, what does Table 5 present? What are the "differences" here? Each table and figure should have a caption that clearly describes its contents.
**ANSWER**: Each table have been captioned with clear description of its contents.

5) Figure 2 is really terrible and should be replaced with a standard figure that shows the behavior of the expected marginal means over time for each group.

**ANSWER**: Figure 2 has been removed

6) **Lines 317-318**: This conclusion seems far too strong for this paper. Has it really been proved to this degree?
**ANSWER**: Those lines have been removed

7) **Lines 338-344**: The authors do not disclose whether similar claims were made by COG patients. This paragraph seems really close to misinformation as it is currently worded. If patients from both treatments reported these claims, then how can they be attributed to Moringa oleifera? All such claims in the discussion need to be backed up by data analysis in the results. Where is the presentation of these results?

**ANSWER**: Those paragraphs have been removed.

8) The methods section lacks critical details. As an example, on Line 175 the authors state that viral loads were measured "by the Laboratory scientist using Real-time PCR machine" (sic). Which machine? What was the protocol? Which laboratory? The same issue pertains to the CD4 counts.

**ANSWER**: Details have been provided. (Pages 7-8; Lines 169-177)

9) Lines 403-405 overstates the results.
**ANSWER**: Those lines have been removed.

10) The conclusion and recommendation in Lines 417-121 do not seem to be fully supported by the manuscript.

**ANSWER**: A new conclusion and recommendation that supports the manuscript have been provided (Page 17; Line 342 [recommendation]); (Page 18; Lines 367-372 [conclusion])

**Reviewer #2**:

 1. Moringa leaf powder packaged in sachets as used in the MOG potentially had a taste distinguishable from COG. This most likely violated the blinding process especially in a community where there was a “high awareness” level of Moringa’s usefulness as a nutraceutical. This should be considered as significant source of bias enough to dispute the conclusion of a causal relationship between the MOG intervention and the observed increase in CD4 count. A more firm conclusion could have been justified if the powder had been packaged in gelatin capsules which could mask the taste and also ensure more accurate and consistent dosing of the product.

**ANSWER**: This factor has been included as a limitation of the study (Page 18; Lines 360 – 365).

Also a conclusion limited to an association between *Moringa oleifera* and increased CD4 count/ anthropometric parameters, citing all the observed shortcomings of the study design has been provided. (Page 18; Lines 367-372 )

2. While literature references were made, the claims that Moringa leaf samples contained essential and non-essential amino acids, wide range of vitamins, minerals, carotenoids, polyphenols, phenolic acids, flavonoids, alkaloids, glucosinolates, isothiocyanates, tannins and saponins were unsubstantiated in this particular study. Perhaps a description of whether the manufacturer Prime Global Agricultural Industries Limited routinely analyzed the samples for the claimed ingredients would have been useful.

**ANSWER**: The paragraphs stating the claims have been removed. The nutritional content of *Moringa oleifera* leaf powder (Nigerian ecotype) analysed by a South African National Accreditation System (SANAS) (SANAS., 2017) accredited laboratory ASPIRATA Food and Beverage Laboratory (ASPIRATA., 2017) has been provided. (Page 9; Lines 217 – 223 and page 16; lines 316 – 319). [Certificates of analysis are attached as supplementary material S3 & S4]

3. Addition of the discussion of the potential of interactions between Moringa and antiretroviral therapy, particularly with efavirenz via alteration of liver metabolism would enrich the manuscript.

**ANSWER**: Discussion on the potential of interaction between Moringa and antiretroviral drugs have been added in the manuscript. (Page 4; Lines 80-86)

4. It is more scientifically acceptable to use the terms such as “study participants” or “HIV infected individuals” instead of “HIV patients”.

**ANSWER**: The scientifically acceptable terms have been used.

5. Sentences on line 174 and line 176 need reconstruction.

**ANSWER**: Details have been provided and the sentences have been reconstructed. (Pages 7-8; Lines 169-177)

6. There may still be more work before a causal relationship can be claimed between the consumption of Moringa leaf powder and observation of increased CD4 count. Review recommends that the conclusion should limited to an association between the intervention and increased CD4 count, siting the observed shortcomings of the study design.

**ANSWER**: A conclusion limited to an association between *Moringa oleifera* and increased CD4 count/ anthropometric parameters have been provided (Page 18; Lines 367-372).

Also all the observed shortcomings of the study design have been included as limitations of the study (Page 18; Lines 360 – 365).

**Reviewer #3:** 

**p11 Selection criteria**
**L105** - Inclusion of patients on only one regimen limits generalisability of the effect on CD4 counts to patients on other regimen. Why was this necessary?

**ANSWER**: During the study conceptualization and design, the concept of limiting the use to only one regimen was to limit sources of bias in the study. The impact of this limitation on the generalizability of the study findings was not envisaged. This factor have been included as limitation in the study. (Page 18; Lines 360 – 365).

Also a large percentage of patients were taking this regimen at the study centre as it was the preferred option at that time (WHO., 2013).

**L113** - Typically, randomisation follows screening and indicates a participant is enrolled into the study. However, under exclusion criteria authors state that patients taking micronutrient or natural health product supplements within 30 days of randomisation were excluded. Please clarify.

The above was an error which has been corrected.

Patients taking micronutrient or natural health product supplements within 30 days of screening were excluded. (Page 5; Lines 109 -110)

**p12 Sample size**
**L122** - In the abstract the authors talk about significance of the mean increase of CD4 count from baseline at the 99% CI which is inconsistent with sample size justification based on a 5% margin of error, i.e. 95% CI.

**ANSWER**: The 99% Cl written in the abstract was an error which has been corrected. (Page 2; Line 34)

**p12 - 13 Intervention**
**L135** - How were the MO leaves positively identified? Was a botanist consulted?

**ANSWER**: Detailed information have been provided. (Page 6; Lines 129 – 132)

**L156** - What seive size was used to prepare the intervention? 5g weight seems too heavy for 1 teaspoon of MO leaf powder.

**ANSWER**: The intervention was prepared using a 0.500mm standard sieve (No 35 mesh size) to obtain fine Moringa oleifera leaves powder. (Page 6; Line 136)

Because of lack of uniformity in spoon sizes, the dosage instruction written on the pack of the intervention was to divide each sachet into three and consume with meals. (Page 7; Line 149- 150)

**L158** - Specify that MO from other sources is what was disallowed.

**ANSWER**: The participants were instructed not to take *Moringa oleifera* from other sources during the study period, has been added (Page 7; Lines 151 – 152)

**p14
L174, L177** - specify the type and volume of blood samples collected and a brief explanation or reference to the details of the laboratory tests.

**ANSWER**: Details have been provided. (Pages 7-8; Lines 169-177)

**p15
L202** - It is the participants who provide consent to participant in the study, not the researchers. This presents a significant ethics issue for this study.

**ANSWER**: The above sentence was a typographical error which has been corrected. (Page 9; Lines 203 – 204)

**p18-19**
Why was the change in anthropometric and immmunological parameters within group important to analyse statistically? Given the objectives of the study, it is the difference between the intervention and control groups that is relevant. The results section is crowded with results that may not be important. In addition, the change from baseline at the end of each month may be more useful than the change between each month. Consider revising the results section and highlighting only the difference in change from baseline between the intervention and control group. One figure and one table, with legends, and focusing on the key results will further enhance the section.

**Response of Reviewer three comments on statistical analysis**

Tables three and four have been merged to generate a new single table as advised by the reviewer. (Page 12-13; Lines 251 – 259)

**p24
L334** - There is no data on which the discussion on 'good adherence' is based. The strategy the authors describe on p13 L161 was to monitor adherence, rather than enhance or optimise it. No results are presented on the level of adherence. In fact a large portion of the discussion refers to perceptions from participants and other operational and implementation aspects that are core to the study and not presented in the results and are appearing for the first time as discussion.

**ANSWER**: The paragraphs stating ‘good adherence’ have been removed.

**p21
L407** - From the power calculation, the study was adequately powered, perhaps a more diverse population of PLWH rather than a larger sample size could be recommended? The recommendations should be based on the effect on MO on CD4 and not extend to issues of food security and nutrition which have not been demonstrated by the current study.
Several challenges and limitations are cited. To what extend were participants reluctant to keep clinic appointments? What was the effect on study outcomes and how was that accounted for in the analysis?

**ANSWERS:**

A more diverse population of PLHIV has been recommended for future studies instead of a larger sample size. (Page 17; Line 342)

The previous paragraphs on recommendation based on food security have been removed.

The effect of participants reluctance to keep clinic appointments did not affect study outcomes as this challenge was envisaged and included in the study design. (Pages 17- 18; Lines 351 – 359).

Several limitations of the study have been cited. (Page 18; Lines 360 – 365).

The write up also has some grammatical errors and could benefit from revision by an English editor. Some comments that may be helpful

**ANSWER**: The services of an English editor have been engaged. A letter from the English editor (Editage) has been attached.
Page 1
L1 - 'oleifera' is always written in lower case and itilised. Correct throughout the manuscript.

**ANSWER**: It has been corrected throughout the manuscript.
L2 - Be consistent with the term used for study population. Ideally, use people first language such as 'people living with HIV (PLWH)' rather than HIV patients or HIV infected patients.

**ANSWER**: The appropriate term have been used.
Page 9
L46 - Change accountable to accounts

**ANSWER**: (Page 3; Line 47)
L48 - revise grammar

ANSWER: The grammar has been revised. (Page 3; Line 48- 50)
L71 - 'leaf' powder & L72 - Sub-Saharan

**ANSWER**: It has been corrected throughout the manuscript.
